# Tautomerism‐Coupled Self‐Assembly and Transformations of Iminopyrrole Metallacages

**DOI:** 10.1002/chem.202502714

**Published:** 2025-09-24

**Authors:** Jakub Sukiennik, Aleksandra Sarwa, Jędrzej P. Perdek, Miłosz Siczek, Bartosz Szyszko

**Affiliations:** ^1^ Faculty of Chemistry University of Wrocław 14 F. Joliot‐Curie St. Wrocław 50–383 Poland

**Keywords:** cage compounds, self‐assembly, supramolecular chemistry, tautomerism, zinc

## Abstract

Four discrete metallacages were obtained from the condensation of 2,5‐diformylpyrrole and *tren* in the presence of Zn(II). The structures differed in nuclearity, size, and symmetry, with the number of templating cations playing a key role in defining the properties of the architecture. The diiminopyrrole coordination motif, a fundamental structural feature of assemblies, undergoes tautomerization in certain cases, yielding an iminoaminoazafulvene, which alters the assembly. The controlled interconversion between bi‐, tetra‐, and dodecanuclear cages was achieved through simple stimuli, highlighting the dynamic nature of these assemblies.

## Graphical abstract

Four metallacages with varying size, symmetry, and nuclearity were formed via the self‐assembly of 2,5‐diformylpyrrole, *tren*, and zinc(II) acetate. Tautomerization of the key diiminopyrrole motif to iminoaminoazafulvene altered the assemblies’ properties. Stimuli‐induced, partially reversible interconversions between bi‐, tetra‐, and dodecanuclear cages revealed their dynamic nature.

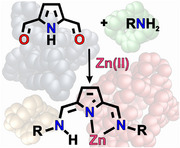

## 1 Introduction

Cages belong to a distinct class of supramolecular systems characterized by an internal cavity capable of accommodating smaller species.^[^ ^1^, ^2^, ^3^ ^]^ This defining feature has led to their diverse applications, including guest binding,^[^ ^4^, ^5^ ^]^ catalysis,^[^ ^6^, ^7^ ^]^ stabilization of reactive species,^[^ ^8^, ^9^ ^]^ drug and bioactive molecule transport,^[^ ^10^, ^11^, ^12^ ^]^ and more.^[^ ^13^, ^14^, ^15^, ^16^ ^]^ Among the various synthetic strategies,^[^ ^17^ ^]^ self‐assembly has emerged as a widely utilized approach toward metallacages.^[^ ^18^, ^19^, ^20^, ^21^ ^]^ The latter enables the integration of small components into intricate structures, provided that the starting materials possess sufficient information.^[^ ^22^ ^]^

The subcomponent self‐assembly, a subclass of coordination‐driven self‐organization processes, involves the in‐situ formation of an organic ligand followed by its metalation.^[^ ^23^, ^24^ ^]^ The method enables the integration of small components into intricate architectures, provided that the starting materials possess sufficient structural information. Multiple covalent (e.g., imine C = N) and coordinative (N→M) bonds are formed simultaneously, resulting in the formation of often high‐symmetry species stabilized by several metal cations.^[^ ^25^, ^26^, ^27^, ^28^, ^29^, ^30^ ^]^ The presence of specific functional groups within the subcomponents, such as carbonyl functionality, enables the cage to undergo further transformations, significantly altering the host‐guest chemistry. In particular, the keto‐enol tautomerism was demonstrated to affect cage properties and reactivity.^[^ ^31^, ^32^, ^33^, ^34^, ^35^ ^]^

**Figure 1:**
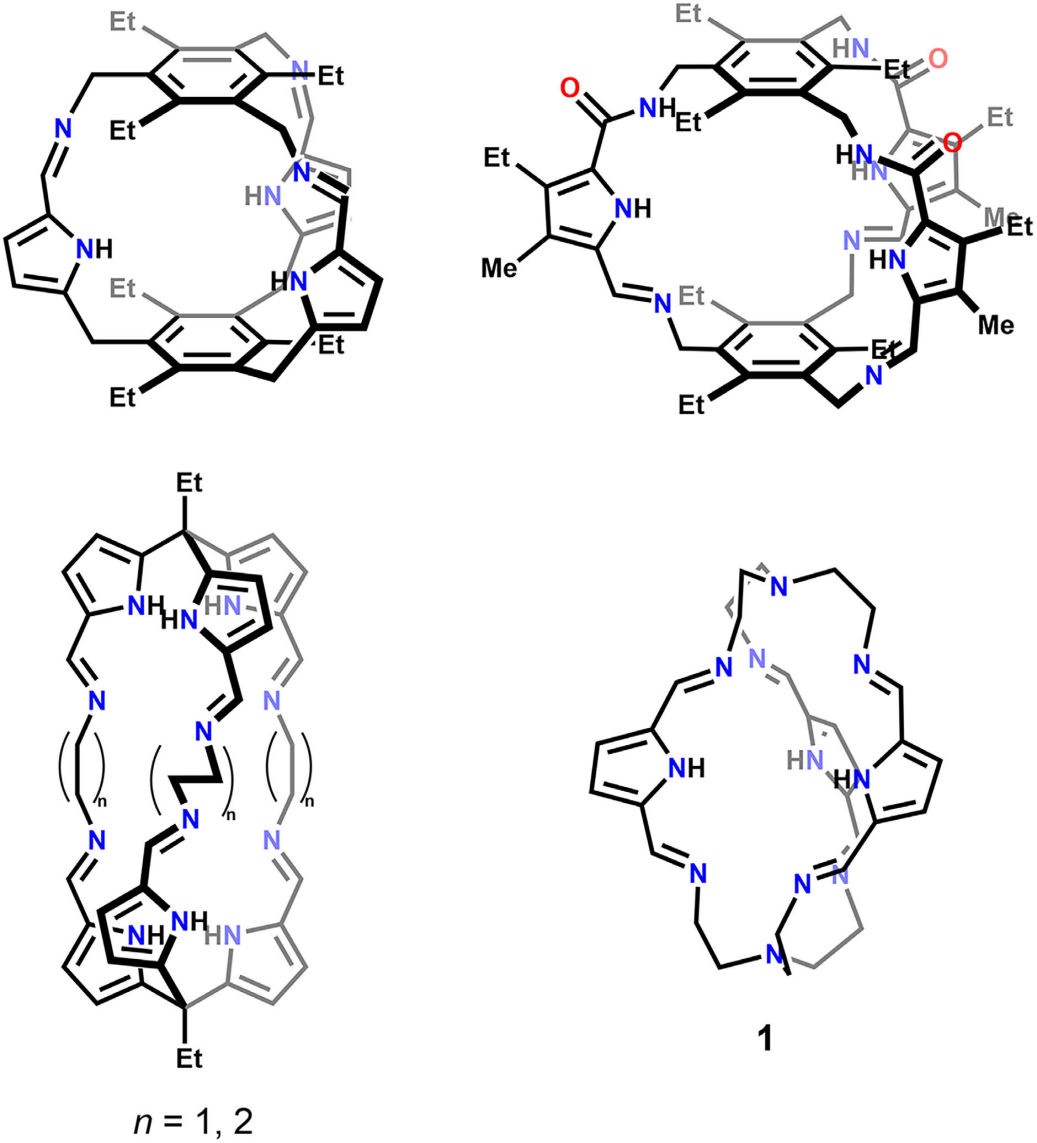
Selected examples of iminopyrrole cages.^[^ ^50^, ^51^, ^52^, ^53^ ^].^

Among the various structural motifs suitable for constructing metallacages using a self‐assembly approach, the iminopyridine scaffold is the most commonly employed, due to the versatile character of the formed ligand toward the transition metals.^[^ ^36^, ^37^, ^38^, ^39^, ^40^ ^]^ Iminopyrrole synthons have been used less frequently in the construction of cages, although, at least in theory, they were expected to perform as well as iminopyridines, while offering the additional advantage of an acidic site, which may enable diverse coordination modes and intriguingly dynamic behavior of the resulting complexes (Figure 1).^[^ ^41^, ^42^, ^43^, ^44^, ^45^, ^46^, ^47^, ^48^, ^49^ ^]^

Several acyclic,^[^ ^54^, ^55^, ^56^, ^57^ ^]^ and cage architectures featuring iminopyrrole motifs have been reported,^[^ ^41^, ^42^, ^50^, ^51^, ^52^, ^53^, ^58^, ^59^, ^60^ ^]^ and have exhibited remarkable capabilities in binding anions,^[^ ^51^, ^52^ ^]^ and organic molecules.^[^ ^53^ ^]^ The intriguing feature of pyrrole‐based metallacapsules is their behavior toward metal cations. Namely, depending on the conditions, they can act as neutral ligands, utilizing the NH groups of pyrrole to stabilize a guest through hydrogen bonding (HB). However, under certain conditions, they may undergo deprotonation on one or more pyrrolic rings, thereby adjusting their properties to accommodate the guest or metal cation. Although these properties are expected to occur in all iminopyrrole cages, redox‐driven deprotonation has, to date, been demonstrated only for the system **1** reported by Nelson.^[^ ^58^, ^59^ ^]^ Our group has shown that **1** can form cascade cryptates, wherein the anion is surrounded by two silver(I) cations located at the poles, or so‐called plenates, with the cavity fully occupied by a cluster of silver(I) ions.^[^ ^41^, ^42^ ^]^

Although iminopyrrole cages have exhibited several intriguing properties, the presence of tautomeric equilibria in their solutions has, to the best of our knowledge, not been reported. In principle, these systems could undergo iminopyrrole–aminoazafulvene tautomeric transformations that may drive their structural rearrangements or affect host–guest chemistry.^[^ ^43^, ^61^, ^62^, ^63^, ^64^, ^65^, ^66^, ^67^, ^68^, ^69^, ^70^, ^71^, ^72^, ^73^, ^74^, ^75^, ^76^, ^77^ ^]^

Our current work demonstrates the interplay between tautomerism and subcomponent self‐assembly. The careful selection of assembly building blocks allows tautomerization to act as a key factor in controlling both the nuclearity and the dimensions of the resulting architectures. It is believed that the selective stabilization of in‐situ‐forming components in a given tautomeric form is driven by the choice of reaction conditions and therefore cannot be decoupled from the self‐assembly process itself. It is believed that the tautomeric state of the building block — likely influenced by the choice of solvent and the anions introduced with the metal cation — appears to play a decisive role in directing the system toward a specific supramolecular architecture.

Herein, we demonstrate the self‐assembly of iminopyrrole cages from 2,5‐diformylpyrrole, tris(2‐aminoethyl)amine (*tren)*, and zinc(II). Depending on the conditions, the metal‐stabilized structures varied in nuclearity, size, and geometry, with the pyrrole‐based building block facilitating a new reaction pathway, referred to as *tautomerism‐coupled subcomponent self‐assembly*. The latter enabled the formation of an unexpected dodecanuclear cage, which could be further transformed into bi‐ or tetranuclear counterparts.

## 2 Results and Discussion

**Scheme 1:**
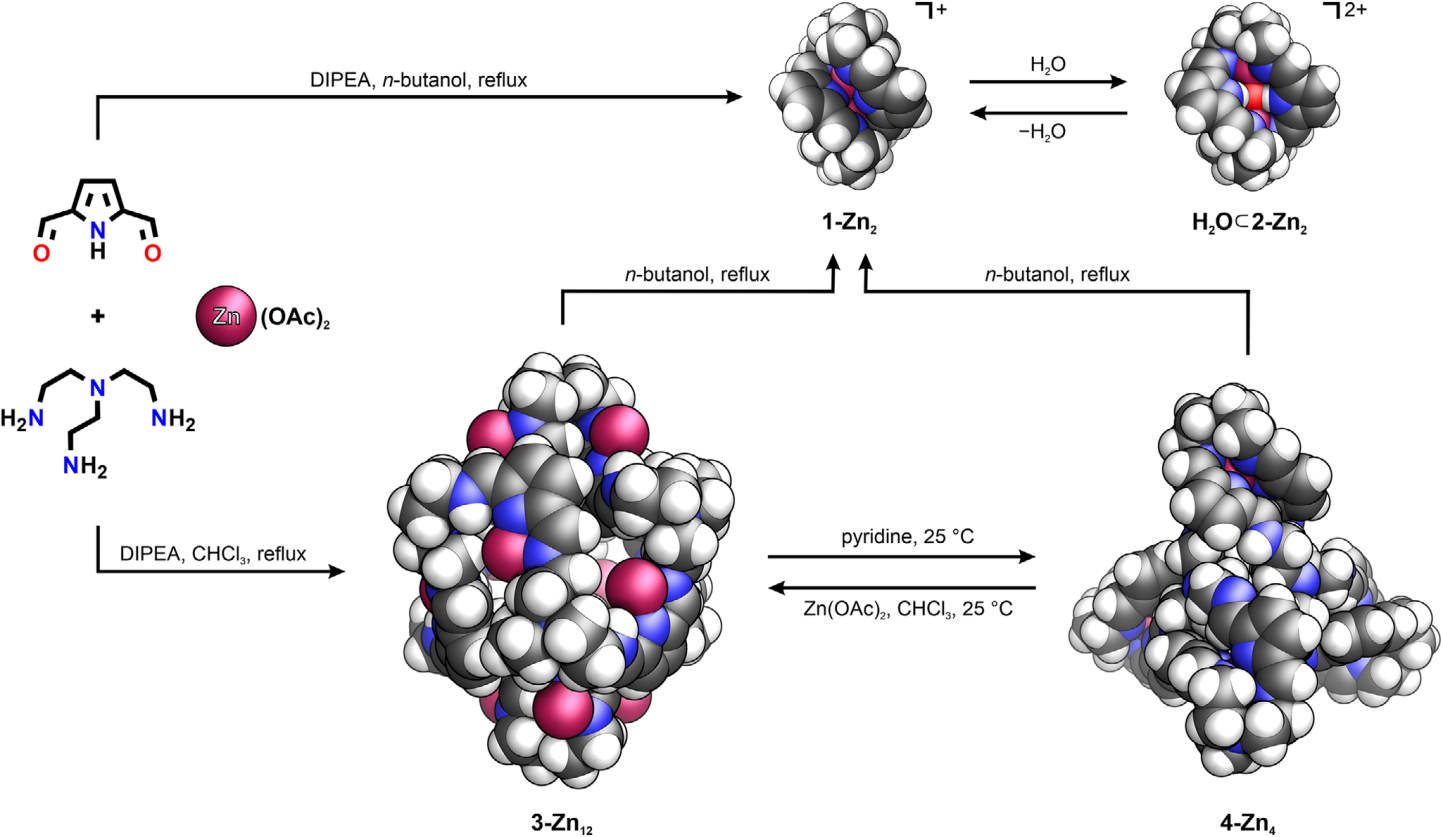
Synthesis and reactivity of iminopyrrole cages.

The reaction of 2,5‐diformylpyrrole, *tren*, and zinc(II) acetate was carried out in *n*‐butanol in the presence of diisopropylethylamine (DIPEA), producing a red‐colored solution (Scheme 1). Upon precipitation with diethyl ether, the [3 + 2] cage **[1‐Zn_2_]OAc** (hereafter simplified as **1‐Zn_2_**), incorporating two zinc(II) cations, was obtained in 95% yield. To obtain a pure product, it was necessary to use an excess of the zinc(II) source during the self‐assembly process. When stoichiometric or near‐stoichiometric amounts of the metal salt were used, the reaction yielded mixtures, from which the isolation of a single, well‐defined product proved challenging.

**Figure 2:**
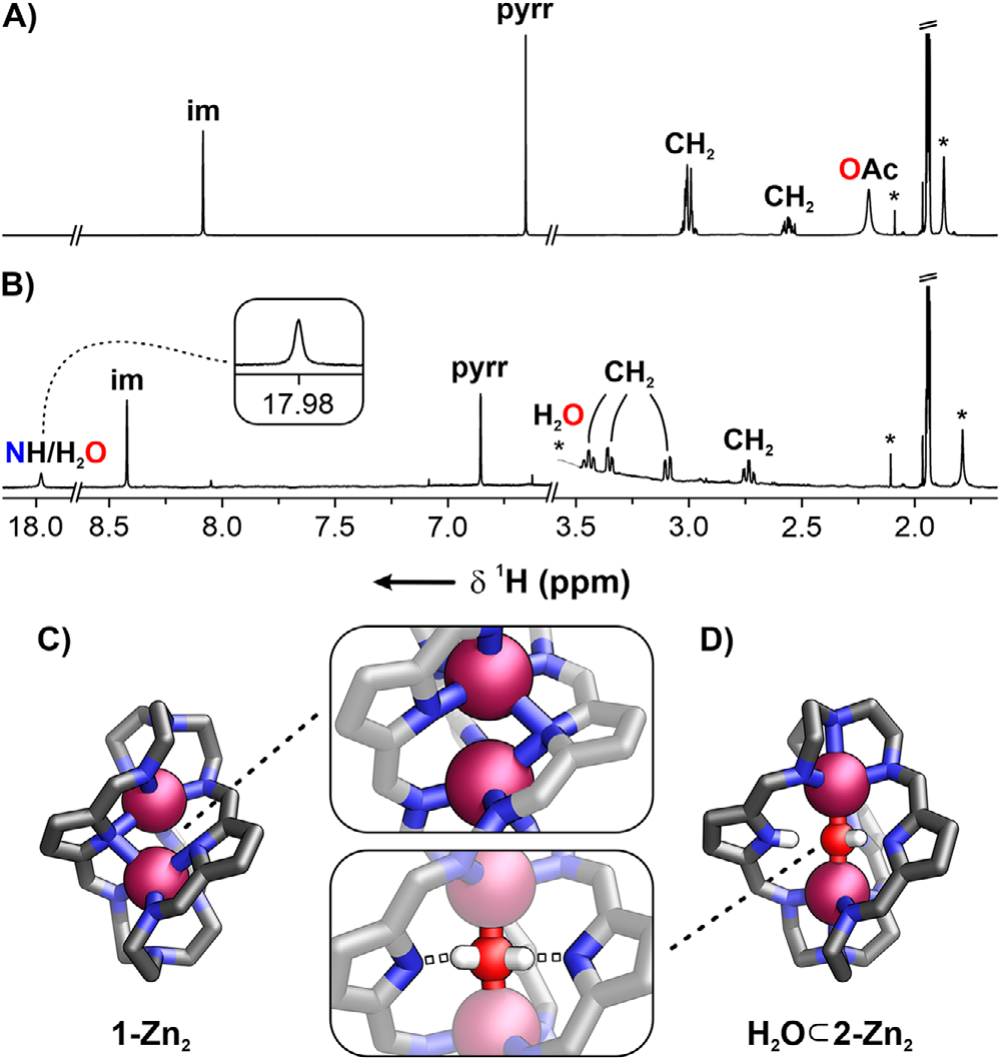
The ^1^H NMR (300 K, CD_3_CN, 600 MHz) spectra of A) **1‐Zn_2_** and B) **H_2_O⊂2‐Zn_2_**; the X‐ray molecular structures of C) **1‐Zn_2_** and D) **H_2_O⊂2‐Zn_2_**.

The electrospray ionization mass spectrometry (ESI‐MS) of **1‐Zn_2_** revealed a signal at 680.1721, corresponding to the C_30_H_36_N_11_Zn_2_ elemental composition (see Supporting Information, Figure S1). The ESI‐MS suggested that the incorporation of two zinc(II) cations required deprotonation of three pyrrole rings, which was further supported by the ^1^H NMR spectrum (Figure 2A, CD_3_CN, 300 K). Aside from the expected single imine resonance at 8.08 ppm, the *β*‐pyrrolic signal appeared at 6.65 ppm, accompanied by methylene group resonances originating from *tren* in the 3.05–2.50 ppm region. Variable‐temperature NMR measurements in CDCl_3_, carried out over the 320–220 K range, did not enable the detection of the NH signal(s) (see Supporting Information, Figure S6).

Single crystals of a binuclear species were obtained through the slow evaporation of **[1‐Zn_2_]OAc** solution in pyridine (Figure 2C).^[^ ^78^ ^]^ Upon crystallization, the anion compensating for the charge of the cationic cryptate was found to have changed. While NMR studies of the product obtained from the self‐assembly reaction indicated that acetate served as the counterion for **[1‐Zn_2_]^+^** (see Supporting Information, Figure S3), the X‐ray molecular structure revealed that the positive charge of the binuclear cryptate was instead neutralized by a mononuclear species, in which one Zn(II) ion was coordinated to three pyrrolide units of the cage (see Supporting Information for detailed analysis, Figure S56). This transformation is likely the result of pyridine acting as a competing ligand, leading to the removal of one Zn(II) cation from the original complex **1‐Zn_2_**, thereby transforming it into **[1‐Zn_2_][1‐Zn]** during crystallization. The transformation illustrated the coordination plasticity of the cage, underscoring that it is highly responsive to external stimuli.

Intriguingly, **1‐Zn_2_** exhibited a coordination motif rarely observed in binuclear cryptates (Figure 2C).^[^ ^59^ ^]^ Both pentacoordinated zinc(II) centers adopted a distorted square pyramidal geometry. Each Zn(II) was bound to three imine nitrogens and two pyrrolides, with one of them displaying bridging *μ*_2_ coordination.^[^ ^79^, ^80^ ^]^ The Zn─N_im_ bond lengths ranged from 2.027(4) to 2.088(4) Å, while the distance between Zn(II) and the bridgehead nitrogens exceeded 2.9 Å. The Zn─N_pyrr_ bonds were slightly longer, ranging from 2.286(4) to 2.426(4) Å.

Upon the crystallization of **1‐Zn_2_** obtained from zinc(II) trifluoroacetate, the unexpected species **H_2_O⊂2‐Zn_2_** was formed (Figure 2D). While **1‐Zn_2_** initially seemed as if it could not accommodate any guest within the cavity due to the limited size, its ability to undergo transformations at the pyrrolide moieties resulted in the formation of **H_2_O⊂2‐Zn_2_**, resembling cascade cryptates.^[^ ^41^, ^81^, ^82^ ^]^ The latter likely formed upon prolonged exposure of **1‐Zn_2_** to trace water in the organic solvent. **H_2_O⊂2‐Zn_2_** was also obtained by a controlled addition of H_2_O to **1‐Zn_2_** under NMR spectroscopy control (see Supporting Information, Figure S48). The reverse transformation was achieved by adding anhydrous Na_2_SO_4_ to **H_2_O⊂2‐Zn_2_** or by evaporating it under a vacuum (see Supporting Information, Figure S49). The transformation preserved the symmetry of the cage, though it resulted in a slight, downfield shift of imine and *β*‐pyrrolic signals to 8.42 and 6.85 ppm, respectively, with minor changes in the methylene region (Figure 2B, CD_3_CN, 300 K). A broad resonance at 17.98 ppm, integrating to three protons, indicated that those within the cavity were highly deshielded, presumably due to the involvement in exceptionally strong hydrogen bonding.

**Figure 3:**
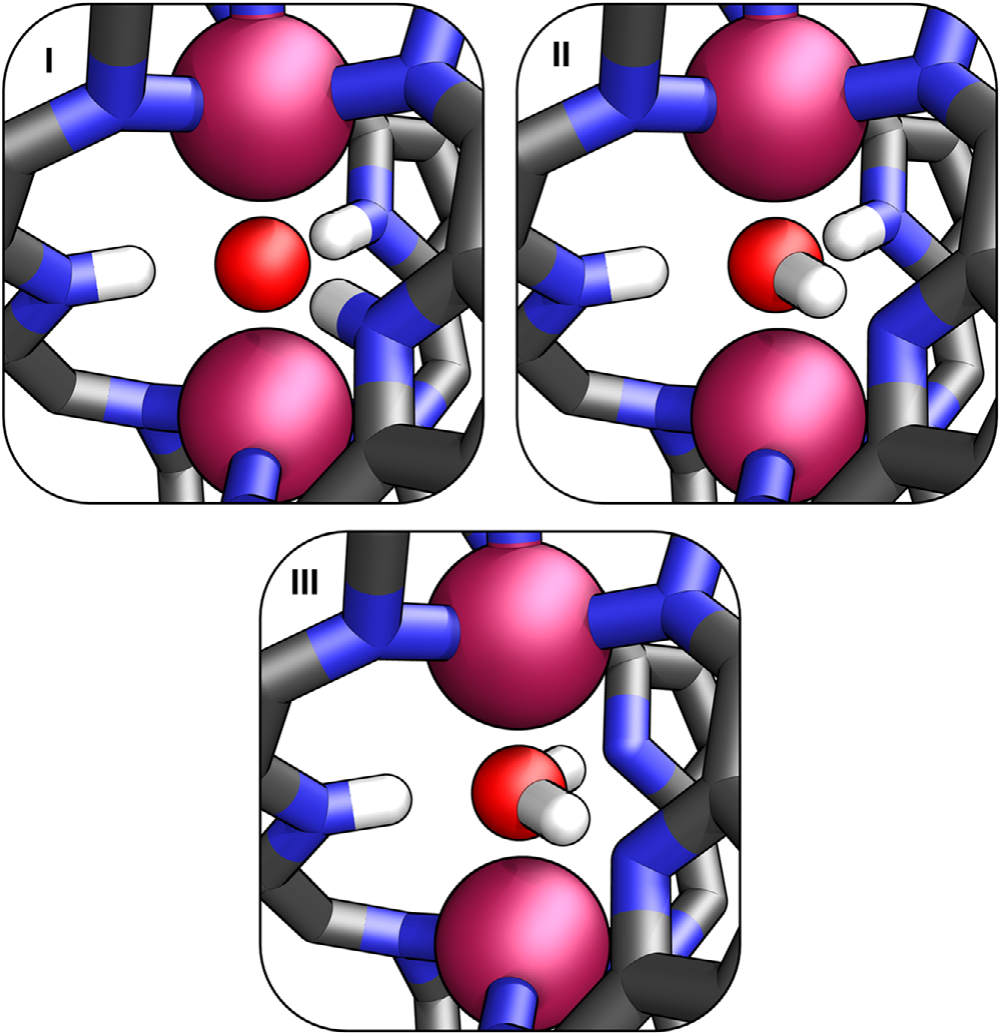
Plausible modes of host‐guest interactions in **H_2_O⊂2‐Zn_2_**.

X‐ray diffraction studies confirmed the high symmetry of **H_2_O⊂2‐Zn_2_** (Figure 2D).^[^ ^78^ ^]^ The cage incorporated two Zn(II) cations at the poles, bound through three imine (2.056(4)–2.116(4) Å) and bridgehead nitrogen each (2.316(3)–2.414(4) Å). The electron density within the cavity has been interpreted as an oxygen‐based species, interacting with the N^−^/NH entities in the meridional region of the cryptate.^[^ ^41^ ^]^ Upon the reaction of **1‐Zn_2_** with water, the conversion of pyrrolide (N^−^ – negatively charged unit) into pyrrole (NH – neutral ligand) functionalities in **2‐Zn_2_** is conceivable, thereby altering the overall charge of the organic ligand (Figure 3). Hence, three structurally varying scenarios can be envisioned: (I) a cage incorporating three neutral pyrrole rings that coordinate a *μ*_2_‐oxo zinc cluster; (II) a cage featuring one pyrrolide and two NH groups binding a hydroxide ion; or (III) two pyrrolides and one pyrrolic NH group stabilizing a neutral water molecule. Although the short intracavity N···H and O···H contacts observed in the solid state hinder precise structural assignment of the host and guest based solely on X‐ray data, the formation of the **H_2_O⊂2‐Zn_2_** complex from **1‐Zn_2_** upon water addition ─ and its facile reversal upon treatment with a drying agent ─ suggests that structure III in Figure 3 most accurately represents its nature.^[^ ^83^, ^84^, ^85^ ^]^

**Figure 4:**
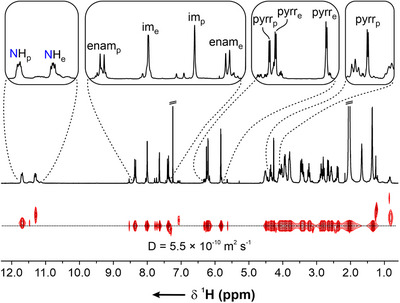
^1^H NMR and DOSY spectra of **3‐Zn_12_** (300 K, CDCl_3_, 600 MHz). The letters “p” and “e” in the subscript correspond to *poles* and the *equator*.

Remarkably, the reaction of 2,5‐diformylpyrrole, *tren*, and Zn(OAc)_2_ in the presence of DIPEA carried out in boiling CHCl_3_ yielded a different product, **3‐Zn_12_**, in 67% yield (Scheme 1). The DOSY NMR confirmed that the multiple signals observed in the spectrum recorded at 300 K in CDCl_3_ originated from the same architecture (Figure 4, see Supporting Information, S31). Due to the inconclusive ESI‐MS of **3‐Zn_12_** (see Supporting Information, Figure S21), the structure elucidation relied on the extensive NMR characterization (see Supporting Information, Figures S26‐31).

The ^1^H NMR spectrum of this new species was entirely distinct from that of **1‐Zn_2_** and **H_2_O⊂2‐Zn_2_**, indicating lower symmetry and a more complex structure (Figure 4). A minor species accompanying the major product could not be identified due to significant spectral overlap with the dominant resonances. The careful analysis of the ^1^H NMR spectrum (CDCl_3_, 300 K) revealed that the assembly consisted of two sets of pyrrole‐based coordination entities. One side of each pyrrole ring incorporated an imine group, while the other featured an enamine functionality. As a result, two amine NH multiplets were observed in the downfield region at 11.71 and 11.31 ppm. The ^1^H‐^1^H COSY spectrum enabled the identification of the corresponding methine CH doublets at 8.35 and 7.38 ppm, respectively (see Supporting Information, Figure S26). The other side of each pyrrole contained an imine group, producing singlets at 7.99 and 7.64 ppm, which exhibited the one‐bond coupling with carbon nuclei at 159.3 and 160.6 ppm, as evidenced by ^1^H‐^13^C HSQC (see Supporting Information, Figure S29). Three *β*‐pyrrolic doublets were observed at 6.24, 6.19, and 5.81 ppm, whereas the fourth one displayed a peculiar chemical shift of 4.25 ppm, suggesting that the proton experienced shielding from the nearby aromatic ring. Furthermore, numerous methylene resonances appeared in the 4.60–2.30 ppm range, underscoring the complex nature of the assembly.

The ^1^H NMR spectrum of **3‐Zn_12_** exhibited significant solvent dependence (see Supporting Information, Figure S23). A similar resonance pattern, characterized by sharp lines, was observed in CDCl_3_, CD_2_Cl_2_, toluene‐*d*_8_, C_6_D_6_, and THF‐*d*_8_.

**Scheme 2:**
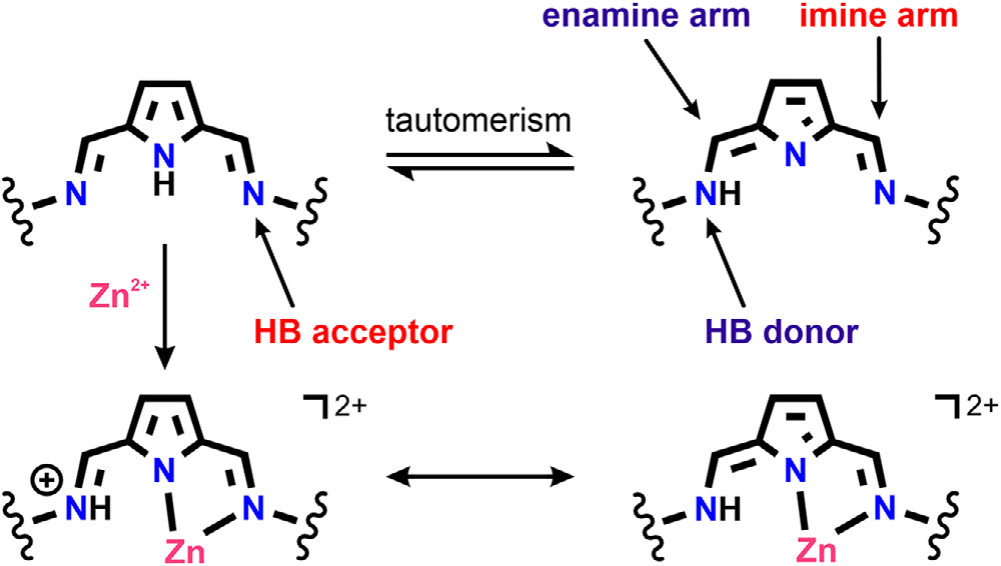
Tautomerism in diiminopyrrole. The remaining ligands on Zn(II) were omitted for clarity.

In contrast, in CD_3_OD, DMSO‐*d*_6_, and DMF‐*d*_7_, **3‐Zn_12_** displayed broad and featureless spectra. This behavior can be explained by considering the tautomerization of the diiminopyrrole to the corresponding iminoaminoazafulvene, which had previously been observed only in acyclic receptors^[^ ^62^, ^63^, ^64^, ^65^, ^66^, ^67^, ^68^ ^]^ and macrocycles.^[^ ^43^, ^69^, ^70^ ^]^ The proton transfer process has profound consequences as it significantly alters the coordination sphere of the transition metal, leading to the conversion of the imine nitrogen into an amine, which serves as a hydrogen bond donor (Scheme 2).^[^ ^61^ ^]^

The solid‐state IR spectrum of **3‐Zn_12_** was recorded to further confirm the tautomerization of the ligand (see Supporting Information, Figure S32). It was anticipated that the stretching frequencies (ν_str_) of imine (C = N) and enamine (C − N) bonds could serve as indicators of the individual tautomers.^[^ ^61^ ^]^ In fact, two bands at 1662 and 1616 cm^−1^ were identified for **3‐Zn_12_**. On the contrary, only one absorption at 1605 cm^−1^ was observed in the IR spectrum of **1‐Zn_2_**, corroborating the bis‐iminopyrrole nature of the coordination motif (see Supporting Information, Figure S13).

**Figure 5:**
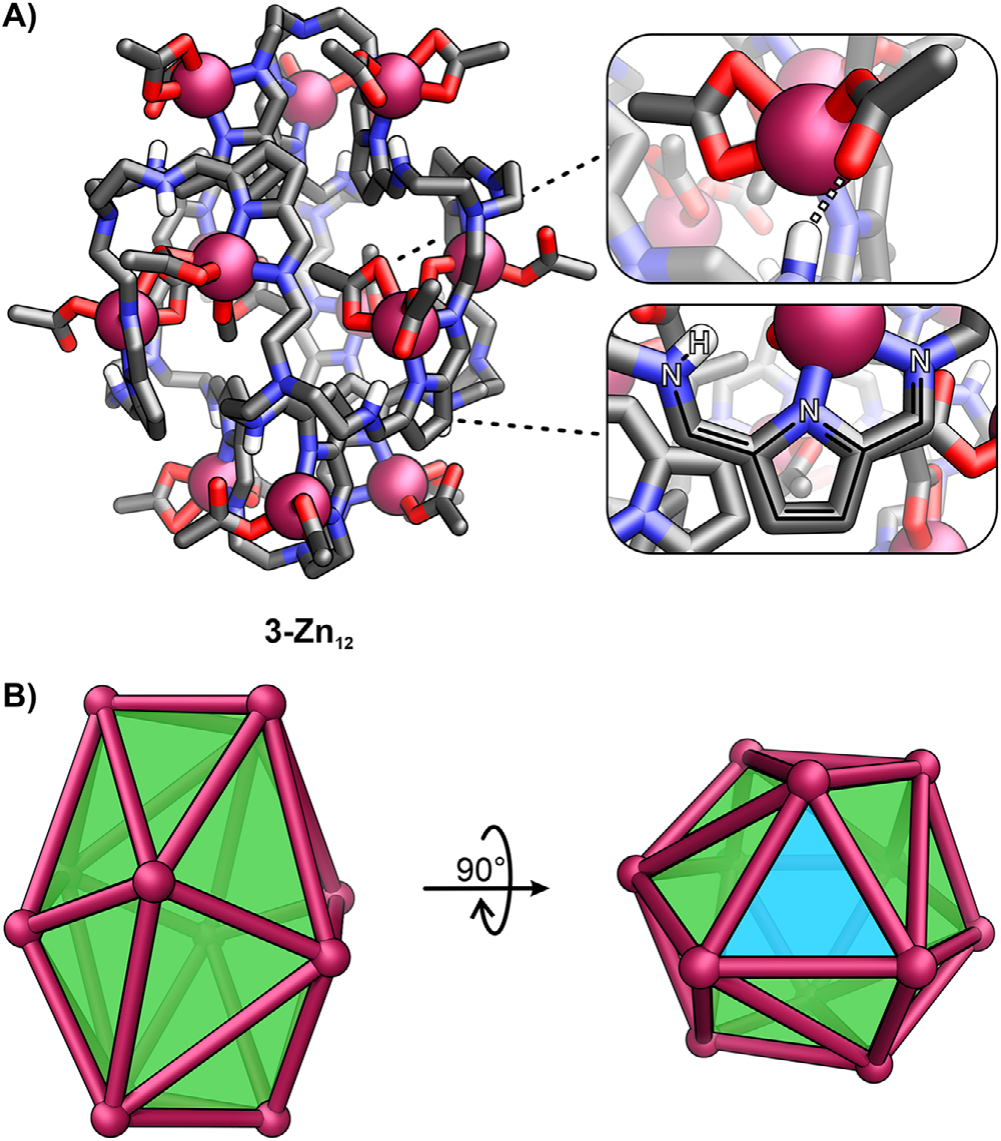
A) The X‐ray molecular structure of **3‐Zn_12_**, and B) its simplified structure comprising twelve Zn(II) cations. The polar region Zn_3_ face was shown in blue.

Single crystals of **3‐Zn_12_** were obtained through slow evaporation from a CHCl_3_ solution.^[^ ^78^ ^]^ The tubular assembly exhibited two polar regions, each comprising three Zn(II) cations and an equatorial part containing six metal centers (Figure 5A). *Tren*‐based caps at opposite ends were linked to pyrrole rings through imine bonds. Each Zn(II) within both triads coordinated two acetate ligands. The amine group proton of the iminoaminoazafulvene was engaged in an HB to the oxygen atom of zinc(II)‐bound acetate. The equatorial region comprised six Zn(II) cations, each bound to six iminoaminoazafulvenes, forming two distinct series – three in an upper belt and another three near the opposite side of the cage. Each tetrahedral Zn(II) coordinated two acetate anions; however, only one participated in HB with the NH group, while the other pointed toward the cavity. With the simplification of the cage structure to twelve Zn(II) cations, it was revealed that they formed the vertices of an irregular icosahedron (Figure 5B). Among its twenty triangular faces, the two associated with the “polar region” Zn(II) cations were equilateral. Though the cage core was potentially spacious enough to accommodate guest molecules, six acetate ligands significantly restricted the available internal volume. The iminoaminoazafulvene coordination motif, a defining feature of the cage, was evident in its bond‐length pattern. As expected, one of the two C*_α_* ─C bonds exhibited a consistent shortening (1.392(7)–1.419(7) Å versus 1.437(6)–1.460(7) Å), stemming from the increased double‐bond character on one side of the pyrrole ring. Similarly, variations in C─N bond distances reflected the two pyrrolic sidearms' differing character, i.e., 1.282(6)–1.302(6) Å versus 1.274(6)–1.287(6) Å, respectively.

**Figure 6:**
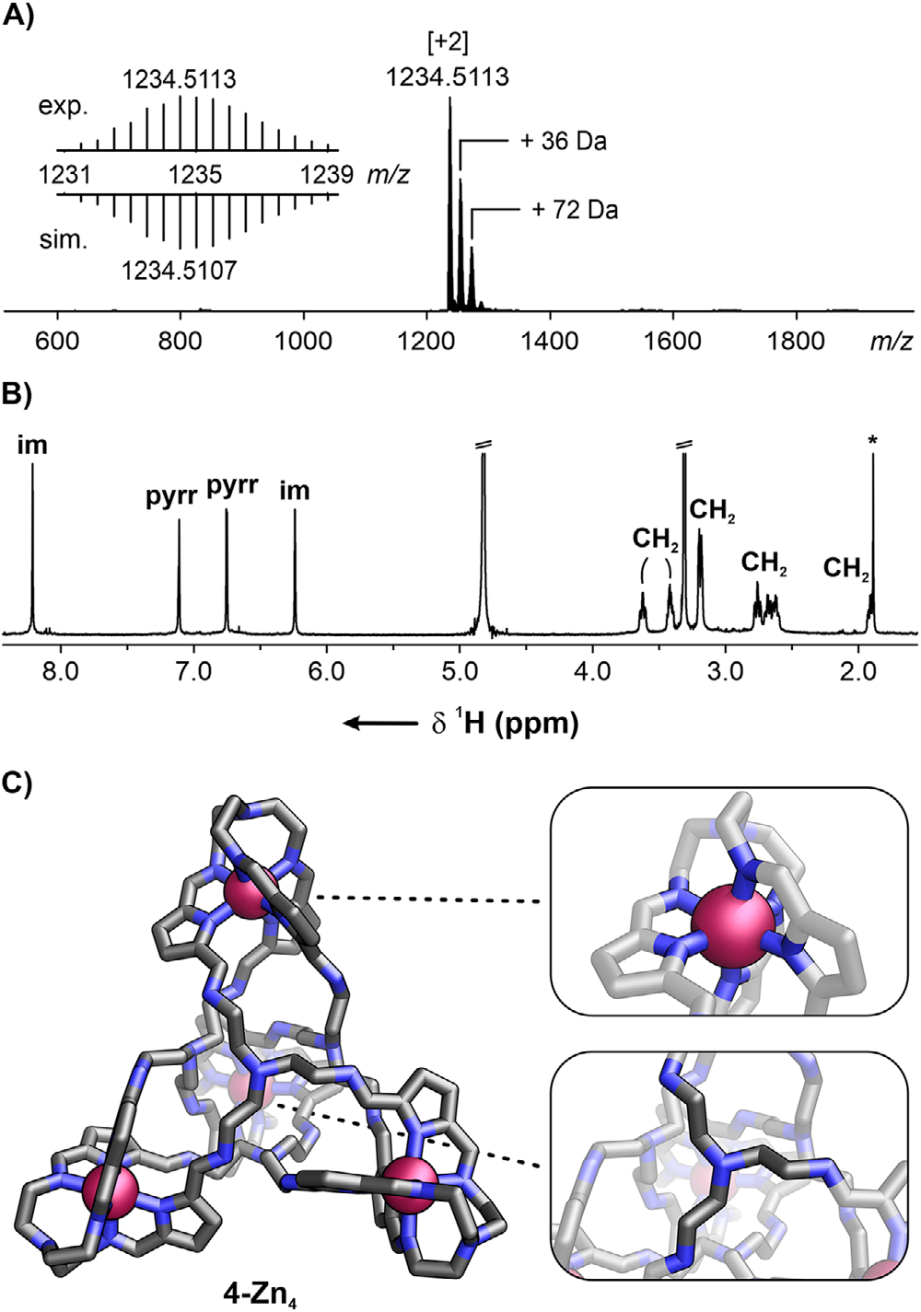
A) The ESI‐MS, B) the ^1^H NMR spectrum (300 K, CD_3_OD, 600 MHz), and C) the X‐ray molecular structure of **4‐Zn_4_**.

Yellow, teardrop‐shaped crystals of **4‐Zn_4_** were obtained in 72% yield upon standing a pyridine solution of **3‐Zn_12_** for 20 hours (Scheme 1).^[^ ^78^ ^]^ The ESI‐MS displayed a +2 ion signal at 1234.5113, corroborating that the cage incorporated four zinc(II) cations (Figure 6A). Given that the alteration in elemental composition did not affect the size of the organic component but solely reduced the number of stabilizing zinc(II) centers, the role of pyridine can be interpreted as that of a competing ligand, capable of displacing eight out of twelve metal ions from the cage. Interestingly, all attempts to obtain the desired product through self‐assembly using a 4:8:12 molar ratio of zinc(II), *tren*, and aldehyde proved unsuccessful. This outcome was consistent regardless of whether CHCl_3_ or *n*‐butanol was used as the solvent, and despite employing zinc(II) acetate as the metal source. In all cases, the reaction mixtures consisted predominantly of **1‐Zn_2_**, with no indication of the formation of the targeted **4‐Zn_4_** species.

The ^1^H NMR spectrum of **4‐Zn_4_**, recorded in CD_3_OD, displayed a simple resonance pattern compared to that of **3‐Zn_12_** (Figure 6B). Two *β*‐pyrrolic signals were observed at 7.11 and 6.75 ppm, accompanied by two imine lines at 8.21 and 6.24 ppm. The latter resulted from the shielding of the methine proton by a nearby aromatic pyrrole ring. Both singlets exhibited a one‐bond correlation to ^13^C resonances at 157.6 and 162.0 ppm, respectively, as identified in the ^1^H‐^13^C HSQC spectrum (see Supporting Information, Figure S44). Methylene protons resonated in the 3.7–1.8 ppm range.

The X‐ray molecular structure of **4‐Zn_4_** revealed a tetrahedral assembly stabilized by four Zn(II) cations at the corners, with the *tren* moieties forming both the vertices and faces of the cage. While the Zn···Zn distances ranged from 12 to 14 Å, the edge lengths — measured between *tren* bridgehead nitrogens — spanned from 17 to 20 Å. Each octahedral Zn(II) was bound to six nitrogen atoms — three from pyrrole rings and three from imine. As a result, at each vertex of the tetrahedron, three nitrogens were not involved in metal coordination. To rationalize the structure of cage **4‐Zn_4_**, several structural scenarios involving tautomerization of the iminopyrrole units to iminoaminoazafulvene‐type moieties were considered.^[^ ^65^ ^]^ Such a tautomeric transformation would alter the overall charge of the cage, necessitating the presence of counterions to maintain charge neutrality. However, the ^1^H NMR spectrum recorded in CD_3_OH displayed no significant deviations from that obtained in CD_3_OD, and the multiplicity of the imine resonances remained unchanged (see Supporting Information, Figure S38). Furthermore, the solid‐state IR spectrum of **4‐Zn_4_** featured a band at 1610 cm^−1^, comparable to that observed for **1‐Zn_2_** (see Supporting Information, Figure S47). Significantly, it was clear that the structure of the cage can vary between the solid state and solution, as well as across solvents of different polarity and proticity.

The transformation of **3‐Zn_12_** into **4‐Zn_4_** upon treatment with pyridine prompted further investigation into the reactivity of assemblies (Scheme 1). The role of pyridine seemed crucial in the reaction, as analogous transformations with EDTA and [2.2.2]cryptand were not observed (see Supporting Information, Figures S53‐S55). However, a small amount of [3 + 2] cage could be identified, suggesting that the contraction is feasible. Hence, **4‐Zn_4_** and **3‐Zn_12_** were subjected to reactions in boiling *n*‐butanol to mimic the self‐assembly conditions for **1‐Zn_2_**. Consequently, the reconfiguration of both complexes to **1‐Zn_2_** was observed (see Supporting Information, Figures S50, S51). To investigate the reverse transformation, namely the expansion of the cages, the addition of Zn(OAc)_2_ to the solution of **4‐Zn_4_** in CHCl_3_ was carried out, resulting in the immediate formation of **3‐Zn_12_** (see Supporting Information, Figure S52). On the other hand, the transformation of **1‐Zn_2_** to **3‐Zn_12_** or **4‐Zn_4_** could not be detected.

## 3 Conclusion

The self‐assembly of 2,5‐diformylpyrrole, tris(2‐aminoethyl)amine, and Zn(II) afforded cage architectures, stabilized by two, four, or twelve templating cations. The diiminopyrrole unit, a key structural motif, engaged in tautomeric equilibria that enabled dynamic structural reconfiguration. Interconversions of the assemblies were triggered by simple stimuli, i.e., the removal of zinc(II) in the presence of a competing ligand altered the symmetry, while refluxing the larger cages in alcoholic solution induced contraction to binuclear species. The coordination flexibility of the binuclear cage allowed for reversible host‐guest complexation with water. It was also demonstrated that the large cage **3‐Zn_12_**, containing clearly defined aminoazafulvene moieties — thus favoring a specific tautomer — forms as a self‐assembly product in chloroform, with its formation requiring the presence of acetate anions coordinated to the metal center. Conversely, alcoholic solvents appear to promote the formation of smaller assemblies, i.e., **1‐Zn_2_**, which is susceptible to protonation — a process that profoundly alters its properties and their interactions with simple guest molecules, such as water. Both cages of this type incorporated iminopyrrole units. The combination of two factors — the coordination plasticity of the employed ligand motif and the (de)protonation capability of the pyrrole rings within the coordination core — proved to be crucial for the formation of the tetrahedral cage **4‐Zn_4_** upon demetalation of **3‐Zn_12_**.

Although this study may represent an initial step toward constructing complex, functional metallacage architectures based on iminopyrrole motifs, we envision that expanding our methodology will pave the way for a broad range of cage‐like structures. Incorporating the iminopyrrole motif into scaffolds amenable to self‐assembly will allow their reactivity to be precisely tuned through careful control of reaction conditions. Particularly, considering that the assemblies reported herein constitute the largest iminopyrrole‐based cages obtained to date, our findings may also encourage further investigations into the design of large‐volume capsules capable of encapsulating guest molecules. The intrinsic adaptability of iminopyrrole‐based components is expected to generate a responsive coordination environment, in which the metal‐stabilized organic framework can reorganize to accommodate encapsulated species. Ongoing studies in our laboratory aim to extend this strategy to other systems.

## Supporting Information

The authors have cited additional references within the Supporting Information.[^86^, ^87^, ^88^, ^89^, ^90^, ^91^, ^92^]

## Conflict of Interest

The authors declare no conflict of interest.

## Supporting information



Supporting Information

Supporting Information

## Data Availability

The data that support the findings of this study are available in the supplementary material of this article.

## References

[1] P. Ballester , M. Fujita , J. Rebek Jr. , Chem. Soc. Rev. 2015, 44, 392. 25504113 10.1039/c4cs90101k

[2] G. Montà‐González , F. Sancenón , R. Martínez‐Máñez , V. Martí‐Centelles , Chem. Rev. 2022, 122, 13636. 35867555 10.1021/acs.chemrev.2c00198 PMC9413269

[3] C. J. T. Cox , J. Hale , P. Molinska , J. E. M. Lewis , Chem. Soc. Rev. 2024, 53, 10380. 39351690 10.1039/d4cs00761a

[4] K. Iizuka , H. Takezawa , M. Fujita , Angew. Chem. Int. Ed. 2025, 64, e202422143. 10.1002/anie.202422143 39635831

[5] F. J. Rizzuto , L. K. S. Von Krbek , J. R. Nitschke , Nat. Rev. Chem. 2019, 3, 204.

[6] Y. Fang , J. A. Powell , E. Li , Q. Wang , Z. Perry , A. Kirchon , X. Yang , Z. Xiao , C. Zhu , L. Zhang , F. Huang , H.‐C. Zhou , Chem. Soc. Rev. 2019, 48, 4707. 31339148 10.1039/c9cs00091g

[7] D. Liu , H. Ma , C. Zhu , F. Qiu , W. Yu , L.‐L. Ma , X.‐W. Wei , Y.‐F. Han , G. Yuan , J. Am. Chem. Soc. 2024, 146, 2275. 38215226 10.1021/jacs.3c14254

[8] D. J. Cram , M. E. Tanner , R. Thomas , Angew. Chem. Int. Ed. Engl. 1991, 30, 1024.

[9] A. Galan , P. Ballester , Chem. Soc. Rev. 2016, 45, 1720. 26797259 10.1039/c5cs00861a

[10] Y.‐H. Huang , Y.‐L. Lu , Z.‐M. Cao , X.‐D. Zhang , C.‐H. Liu , H.‐S. Xu , C.‐Y. Su , J. Am. Chem. Soc. 2024, 146, 21677. 39042557 10.1021/jacs.4c05758

[11] A. T. Veetil , K. Chakraborty , K. Xiao , M. R. Minter , S. S. Sisodia , Y. Krishnan , Nat. Nanotechnol. 2017, 12, 1183. 28825714 10.1038/nnano.2017.159

[12] Q.‐H. Ling , Z.‐C. Lou , L. Zhang , T. Jin , W.‐T. Dou , H.‐B. Yang , L. Xu , Chem. Soc. Rev. 2024, 53, 6042. 38770558 10.1039/d3cs01081c

[13] D. Zhang , T. K. Ronson , Y.‐Q. Zou , J. R. Nitschke , Nat. Rev. Chem. 2021, 5, 168. 37117530 10.1038/s41570-020-00246-1

[14] M. Wilms , L. V. Melendez , R. J. Hudson , C. R. Hall , S. P. Ratnayake , T. Smith , E. D. Gaspera , G. Bryant , T. U. Connell , D. E. Gómez , Angew. Chem. Int. Ed. 2023, 62, e202303501. 10.1002/anie.202303501 37186332

[15] E. G. Percástegui , T. K. Ronson , J. R. Nitschke , Chem. Rev. 2020, 120, 13480. 33238092 10.1021/acs.chemrev.0c00672 PMC7760102

[16] N. Ahmad , H. A. Younus , A. H. Chughtai , F. Verpoort , Chem. Soc. Rev. 2015, 44, 9. 25319756 10.1039/c4cs00222a

[17] G. Zhang , M. Mastalerz , Chem. Soc. Rev. 2014, 43, 1934. 24336604 10.1039/c3cs60358j

[18] S. Pullen , J. Tessarolo , G. H. Clever , Chem. Sci. 2021, 12, 7269. 34163819 10.1039/d1sc01226f PMC8171321

[19] M. M. J. Smulders , I. A. Riddell , C. Browne , J. R. Nitschke , Chem. Soc. Rev. 2013, 42, 1728. 23032789 10.1039/c2cs35254k

[20] L. Chen , Q. Chen , M. Wu , F. Jiang , M. Hong , Acc. Chem. Res. 2015, 48, 201. 25517043 10.1021/ar5003076

[21] B. H. Northrop , Y.‐R. Zheng , K.‐W. Chi , P. J. Stang , Acc. Chem. Res. 2009, 42, 1554. 19555073 10.1021/ar900077c PMC2764814

[22] R. J. Sarma , J. R. Nitschke , Angew. Chem. Int. Ed. 2008, 47, 377. 10.1002/anie.200703877 18022884

[23] T. K. Ronson , S. Zarra , S. P. Black , J. R. Nitschke , Chem. Commun. 2013, 49, 2476. 10.1039/c2cc36363a 23289097

[24] D. Zhang , T. K. Ronson , J. R. Nitschke , Acc. Chem. Res. 2018, 51, 2423. 30207688 10.1021/acs.accounts.8b00303

[25] H. Zhu , N. M. A. Speakman , T. K. Ronson , J. R. Nitschke , Acc. Chem. Res. 2025, 58, 1296. 40132056 10.1021/acs.accounts.5c00081 PMC12004452

[26] C. F. Espinosa , T. K. Ronson , J. R. Nitschke , J. Am. Chem. Soc. 2023, 145, 9965. 37115100 10.1021/jacs.3c00661 PMC10176475

[27] H. Zhu , L. Pesce , R. Chowdhury , W. Xue , K. Wu , T. K. Ronson , R. H. Friend , G. M. Pavan , J. R. Nitschke , J. Am. Chem. Soc. 2024, 146, 2379. 38251985 10.1021/jacs.3c11321 PMC10835658

[28] J. P. Carpenter , T. K. Ronson , F. J. Rizzuto , T. Héliot , P. Grice , J. R. Nitschke , J. Am. Chem. Soc. 2022, 144, 8467. 35511929 10.1021/jacs.2c02261 PMC9121369

[29] M. Xu , X. Jing , B. Sun , C. He , J. N. H. Reek , C. Duan , Angew. Chem. Int. Ed. 2023, 62, e202310420. 10.1002/anie.202310420 37661189

[30] Y. Qin , Q. Ling , Y. Wang , Y. Hu , L. Hu , X. Zhao , D. Wang , H. Yang , L. Xu , B. Z. Tang , Angew. Chem. Int. Ed. 2023, 62, e202308210. 10.1002/anie.202308210 37452485

[31] S. Bera , A. Basu , S. Tothadi , B. Garai , S. Banerjee , K. Vanka , R. Banerjee , Angew. Chem. Int. Ed. 2017, 56, 2123. 10.1002/anie.201611260 28097801

[32] Q. Shi , X. Zhou , W. Yuan , X. Su , A. Neniškis , X. Wei , L. Taujenis , G. Snarskis , J. S. Ward , K. Rissanen , J. De Mendoza , E. Orentas , J. Am. Chem. Soc. 2020, 142, 3658. 31983204 10.1021/jacs.0c00722

[33] D. Al Kelabi , A. Dey , L. O. Alimi , H. Piwoński , S. Habuchi , N. M. Khashab , Chem. Sci. 2022, 13, 7341. 35799823 10.1039/d2sc00836j PMC9214840

[34] D.‐X. Cui , Y. Geng , J.‐N. Kou , G.‐G. Shan , C.‐Y. Sun , K.‐H. Zhang , X.‐L. Wang , Z.‐M. Su , Nat. Commun. 2022, 13, 4011/1–8. 35817768 10.1038/s41467-022-31785-4 PMC9273608

[35] L.‐P. Zhou , X.‐S. Feng , S.‐J. Hu , Q.‐F. Sun , J. Am. Chem. Soc. 2023, 145, 17845. 37545096 10.1021/jacs.3c04921

[36] M. Zenka , J. Preinl , E. Pertermann , A. Lützen , K. Tiefenbacher , Eur. J. Inorg. Chem. 2023, 26, e202300110.

[37] L. R. Holloway , P. M. Bogie , Y. Lyon , C. Ngai , T. F. Miller , R. R. Julian , R. J. Hooley , J. Am. Chem. Soc. 2018, 140, 8078. 29913069 10.1021/jacs.8b03984

[38] F. J. Rizzuto , J. R. Nitschke , J. Am. Chem. Soc. 2020, 142, 7749. 32275828 10.1021/jacs.0c02444 PMC7304868

[39] H. Xu , T. K. Ronson , A. W. Heard , P. C. P. Teeuwen , L. Schneider , P. Pracht , J. D. Thoburn , D. J. Wales , J. R. Nitschke , Nat. Chem. 2025, 17, 289. 39779971 10.1038/s41557-024-01708-5 PMC11794150

[40] R. Lavendomme , T. K. Ronson , J. R. Nitschke , J. Am. Chem. Soc. 2019, 141, 12147. 31287669 10.1021/jacs.9b06182 PMC6756589

[41] A. Sarwa , A. Białońska , M. Garbicz , B. Szyszko , Chem. Eur. J. 2023, 29, e202203850. 36594926 10.1002/chem.202203850

[42] B. Trzaskowski , J. P. Martínez , A. Sarwa , B. Szyszko , W. A. Goddard , J. Phys. Chem. A 2024, 128, 3339. 38651289 10.1021/acs.jpca.4c01464 PMC11077489

[43] A. Sarwa , A. Białońska , M. Sobieraj , J. P. Martínez , B. Trzaskowski , B. Szyszko , Angew. Chem. Int. Ed. 2024, 63, e202316489. 10.1002/anie.202316489 38032333

[44] V. I. Minkin , M. S. Korobov , L. E. Nivororozhkin , O. E. Kompan , R. Ya Olekhnovich , G. S. Borodkin , Y. T. Struchkov , Mendeleev Commun. 1993, 3, 2.

[45] P. Daneshmand , I. Michalsky , P. M. Aguiar , F. Schaper , Dalton Trans. 2018, 47, 16279. 30398252 10.1039/c8dt02562b

[46] Y. Chen , W. Chiu , T. Hu , C. Lin , J. Huang , J. Chin. Chem. Soc. 2015, 62, 133.

[47] S. A. Carabineiro , L. C. Silva , P. T. Gomes , L. C. J. Pereira , L. F. Veiros , S. I. Pascu , M. T. Duarte , S. Namorado , R. T. Henriques , Inorg. Chem. 2007, 46, 6880. 17658870 10.1021/ic062125w

[48] T. Li , J. Jenter , P. W. Roesky , Z. Anorg. Allg. Chem. 2010, 636, 2148.

[49] K. Wu , T. K. Ronson , P. Su , Z. Chen , L. Goh , A. W. Heard , X. Li , F. Klautzsch , C. A. Schalley , M. Vinković , J. R. Nitschke , Nat. Synth. 2023, 2, 789.

[50] D. Chen , A. E. Martell , Tetrahedron 1991, 47, 6895.

[51] H. J. Han , J. H. Oh , J. L. Sessler , S. K. Kim , Chem. Commun. 2019, 55, 10876. 10.1039/c9cc05613k 31433411

[52] J. H. Oh , J. H. Kim , D. S. Kim , H. J. Han , V. M. Lynch , J. L. Sessler , S. K. Kim , Org. Lett. 2019, 21, 4336. 31125242 10.1021/acs.orglett.9b01515

[53] O. D. Fox , T. D. Rolls , P. D. Beer , M. G. B. Drew , Chem. Commun. 2001, 1632. 10.1039/b104077b 12240417

[54] H. Hao , S. Bhandari , Y. Ding , H. W. Roesky , J. Magull , H.‐G. Schmidt , M. Noltemeyer , C. Cui , Eur. J. Inorg. Chem. 2002, 2002, 1060.

[55] J. Lewiński , M. Dranka , I. Kraszewska , W. Śliwiński , I. Justyniak , Chem. Commun. 2005, 4935. 10.1039/b509669c 16205805

[56] J. Lewiński , K. Suwała , T. Kaczorowski , M. Gałęzowski , D. T. Gryko , I. Justyniak , J. Lipkowski , Chem. Commun. 2009, 215. 10.1039/b813315h 19099073

[57] J. Lewiński , M. Kościelski , K. Suwała , I. Justyniak , Angew. Chem. Int. Ed. 2009, 48, 7017. 10.1002/anie.200902716 19691076

[58] L. Qin , J. Nelson , M. McCann , J. Inorg. Biochem. 1993, 51, 633.

[59] Q. Lu , V. McKee , J. Nelson , J. Chem. Soc., Chem. Commun. 1994, 649.

[60] F. Wang , E. Sikma , Z. Duan , T. Sarma , C. Lei , Z. Zhang , S. M. Humphrey , J. L. Sessler , Chem. Commun. 2019, 55, 6185. 10.1039/c9cc02490e 31080980

[61] M. Giri , D. Sahoo , B. P. Samantray , P. P. Sahoo , S. Mishra , T. Guchhait , J. Mol. Struct. 2024, 1308, 138029/1.

[62] A. J. McNeece , M.‐C. Chang , A. S. Filatov , J. S. Anderson , Inorg. Chem. 2018, 57, 7044. 29798666 10.1021/acs.inorgchem.8b00737

[63] S. K. Loke , E. Pagadala , S. Devaraju , V. Srinivasadesikan , R. K. Kottalanka , RSC Adv. 2020, 10, 36275. 35517922 10.1039/d0ra07837a PMC9057005

[64] S. D. Reid , C. Wilson , A. J. Blake , J. B. Love , Dalton Trans. 2010, 39, 418. 10.1039/b909842a 20023977

[65] J. S. Hart , F. J. White , J. B. Love , Chem. Commun. 2011, 47, 5711. 10.1039/c1cc11378j 21499619

[66] C. A. Leahy , M. J. Drummond , J. Vura‐Weis , A. R. Fout , Dalton Trans. 2021, 50, 12088. 34519757 10.1039/d1dt02585f

[67] E. M. Matson , Z. Gordon , B. Lin , M. J. Nilges , A. R. Fout , Dalton Trans. 2014, 43, 16992. 25325404 10.1039/c4dt02327g

[68] M. J. Drummond , C. L. Ford , D. L. Gray , C. V. Popescu , A. R. Fout , J. Am. Chem. Soc. 2019, 141, 6639. 30969766 10.1021/jacs.9b01516

[69] S. Ali Naghi Taheri , R. A. Jones , S. S. Badesha , M. M. Hania , Tetrahedron 1989, 45, 7717.

[70] J. L. Sessler , E. Tomat , V. M. Lynch , Chem. Commun. 2006, 4486. 10.1039/b608143f 17283793

[71] R. Kumar , G. Mani , Dalton Trans. 2015, 44, 6896. 25771810 10.1039/c5dt00438a

[72] M. Matviyishyn , K. A. Konieczny , B. Trzaskowski , B. Szyszko , Chem. Eur. J. 2024, 30, e202402932. 39196848 10.1002/chem.202402932

[73] R. Kumar , T. Guchhait , V. Subramaniyan , C. Schulzke , G. Mani , Dalton Trans. 2020, 49, 13840. 33006344 10.1039/d0dt02964e

[74] P. Eswar , L. S. Krishna , V. Srinivasadesikan , R. K. Kottalanka , J. Mol. Struct. 1244, 2021, 131030/1.

[75] H.‐L. Cho , K. L. Gullett , A. R. Fout , Chem. Commun. 2024, 60, 10564. 10.1039/d4cc03186e 39229921

[76] E. M. Matson , J. A. Bertke , A. R. Fout , Inorg. Chem. 2014, 53, 4450. 24758308 10.1021/ic500102c

[77] E. M. Matson , Y. J. Park , J. A. Bertke , A. R. Fout , Dalton Trans. 2015, 44, 10377. 25970267 10.1039/c5dt00985e

[78] D. numbers , 2452187 (for **1‐Zn_2_ **), 2452188 (for **H_2_O⊂2‐Zn_2_ **), 2452189 (for **3‐Zn_12_ **), and 2452190 (for **4‐Zn_4_ **) contain the supplementary crystallographic data for this paper. These data are provided free of charge by the joint Cambridge Crystallographic Data Centre and Fachinformationszentrum Karlsruhe Access Structures service.

[79] S. Anga , I. Banerjee , H. P. Nayek , T. K. Panda , RSC Adv. 2016, 6, 80916.

[80] J. Jenter , P. W. Roesky , New J. Chem. 2010, 34, 1541.

[81] R. J. Motekaitis , A. E. Martell , J. M. Lehn , E. Watanabe , Inorg. Chem. 1982, 21, 4253.

[82] J. M. Lehn , Acc. Chem. Res. 1978, 11, 49.

[83] T. Cadenbach , J. R. Pankhurst , T. A. Hofmann , M. Curcio , P. L. Arnold , J. B. Love , Organometallics 2015, 34, 2608.

[84] R. Kumar , S. C. Sahoo , P. K. Nanda , Eur. J. Inorg. Chem. 2021, 2021, 1057.

[85] D. Prochowicz , K. Sokołowski , J. Lewiński , Coord. Chem. Rev. 2014, 270–271, 112.

[86] Rigaku Oxford Diffraction , CrysAlisPro Software system 2023, version 171.43.104a, Rigaku Corporation, Wrocław, Poland.

[87] G. M. Sheldrick , Acta Crystallogr. Sect. A: Found. Adv. 2015, 71, 3. 25537383 10.1107/S2053273314026370 PMC4283466

[88] G. M. Sheldrick , Acta Crystallogr. Sect. C: Struct. Chem. 2015, 71, 3. 25567568 10.1107/S2053229614024218 PMC4294323

[89] O. V. Dolomanov , L. J. Bourhis , R. J. Gildea , J. A. K. Howard , H. Puschmann , J. Appl. Crystallogr. 2009, 42, 339. 10.1107/S0021889811041161 PMC3236671 22199401

[90] S. Hammes‐Schiffer , A. A. Stuchebrukhov , Chem. Rev. 2010, 110, 6939. 21049940 10.1021/cr1001436 PMC3005854

[91] K. S. Peters , Acc. Chem. Res. 2009, 42, 89. 18781778 10.1021/ar8001156

[92] G. M. Peters, J. B. Winegrad, M. R. Gau, G. H. Imler, B. Xu, S. Ren, B. B. Wayland, M. J. Zdilla, Inorg. Chem. 2017, 56, 3377. 28240905 10.1021/acs.inorgchem.6b02898

