# Supplementary material for: Tautomerism‐Coupled Self‐Assembly and Transformations of Iminopyrrole Metallacages
Source: Chemistry. 2025 Sep 24;31(63):e02714. doi: 10.1002/chem.202502714 (PMC12683226; doi:10.1002/chem.202502714)
Supplement: Supplementary file 1 — Supporting Information [file CHEM-31-e02714-s001.pdf]

Supporting Information

Tautomerism-Coupled Self-Assembly and Transformations of Iminopyrrole Metallacages

Jakub Sukiennik, Aleksandra Sarwa, Jędrzej P. Perdek, Miłosz Siczek, Bartosz Szyszko*

ABSTRACT: Four discrete metallacages were obtained from the condensation of 2,5-diformylpyrrole and tren in the presence of Zn(II). The structures differed in nuclearity, size, and symmetry, with the number of templating cations playing a key role in defining the properties of the architecture. The diiminopyrrole coordination motif, a fundamental structural feature of assemblies, undergoes tautomerisation in certain cases, yielding an iminoaminoazafulvene, which alters the assembly. The controlled interconversion between bi-, tetra-, and dodecanuclear cages was achieved through simple stimuli, highlighting the dynamic nature of these assemblies.

Table of contents

Experimental Procedures ... 3

NMR spectroscopy ... 3

Mass spectrometry ... 3

FT-IR spectroscopy ... 3

X-ray diffraction data ... 3

Synthesis ... 5

Cage [1-Zn_2]OAc ... 5

Cage [1-Zn_2]CF_3COO ... 6

Cage $H_2O \subset 2\text{-}Zn_2$ ... 6

Cage 3-Zn_{12} ... 7

Cage 4-Zn_4 ... 8

Analytical data for [1-Zn_2]OAc ... 9

Analytical data for [1-Zn_2]CF_3COO ... 18

Analytical data for $H_2O \subset 2\text{-}Zn_2$ ... 20

Analytical data for 3-Zn_{12} ... 22

Analytical data for 4-Zn_4 ... 31

Transformations of cages ... 41

Transformation of [1-Zn_2]OAc to $H_2O \subset 2\text{-}Zn_2$ ... 41

Contraction of 3-Zn_{12} to [1-Zn_2]OAc ... 43

Contraction of 4-Zn_4 to [1-Zn_2]X ... 45

Transformation of 4-Zn_4 to 3-Zn_{12} ... 47

X-ray crystallography ... 50

References ... 59

Experimental Procedures

NMR spectroscopy

The NMR spectra were recorded on high-field spectrometers: a 600.15 MHz spectrometer equipped with broadband inverse and observe gradient probes, and a 500.16 MHz spectrometer equipped with a broadband observe gradient probe. The ^{1}H and ^{13}C NMR spectra were referenced to the residual solvent signal of $CDCl_3$ (^{1}H NMR: δ = 7.24 ppm, ^{13}C NMR: δ = 77.06 ppm), CD_3OD (^{1}H NMR: δ = 3.31 ppm, ^{13}C NMR: δ = 49.0 ppm), CD_3OH (^{1}H NMR: δ = 3.31 ppm), CD_2Cl_2 (^{1}H NMR: δ = 5.32 ppm), toluene-d_8 (^{1}H NMR: δ = 7.09, 7.01, 6.97, 2.08 ppm), C_6D_6 (^{1}H NMR: δ = 7.16 ppm), THF-d_8 (^{1}H NMR: δ = 3.58, 1.72 ppm), DMSO-d_6 (^{1}H NMR: δ = 2.50 ppm), DMF-d_7 (^{1}H NMR: δ = 8.01, 2.91, 2.74 ppm), CD_3CN (^{1}H NMR: δ = 1.94 ppm, ^{13}C NMR: δ = 118.3 ppm), acetone-d_6 (^{1}H NMR: δ = 2.05 ppm), D_2O (^{1}H NMR: δ = 4.81 ppm).

Mass spectrometry

The mass spectra were recorded on a Bruker qTOF compact spectrometer.

FT-IR spectroscopy

The FT-IR spectra were recorded on a Shimadzu IRSpirit-T.

X-ray diffraction data

Single-crystal X-ray diffraction data for all crystals were collected at 100 K on a Rigaku XtalLAB Synergy R diffractometer equipped with a HyPix-Arc 150 HPAD detector and Cu-Kα rotating anode. The diffraction images were processed using CrysAlisPro software.[86] All structures were solved by ShelXT[87] and refined by a ShelXL full matrix least-squares method on F2[88] using the Olex2 software suite.[89] All crystal structures include some disorder. Detailed information about disorder treatment is available in the CIF files.

Cage 1-Zn_2

Crystals suitable for SCXRD analysis were grown *via* slow evaporation of a pyridine solution of **[1-Zn_2]OAc**. The charge of the binuclear cationic cryptate **[1-Zn_2]$^+$** was compensated by an anion comprising **[1-Zn]** species. **[1-Zn]$^-$** constituted 67% of anions, whereas the remaining 33% can be described as a **[1-ZnHOAc]$^-$**, where a single imine group was protonated, interacting with CH_3COO^- through a hydrogen bond. In **[1-Zn]$^-$**, the position of the acetate was occupied by pyridine. The acetate's C–C distance was restrained to 1.54(2) Å. Additionally, the position of its carboxylic carbon atom was strongly overlapped with a carbon atom of the disordered pyridine and thus their atomic displacement parameters were constrained and refined together using the EADP instruction. The structure was deposited in CCDC with deposition number #2452187.

Cage $H_2O\subset$2-Zn_2

Crystals suitable for SCXRD analysis were grown *via* slow evaporation of $CHCl_3$ solution. The exact positions of the pyrrolic protons cannot be determined reliably by X-ray diffraction data. Experimental data suggest a proton transfer process[90,91] between pyrrolic nitrogen and the central oxygen atom: the cage **$H_2O\subset$2-Zn_2** can be formed from **1-Zn_2** through the addition of water, and then transformed back under vacuum or by drying over anhydrous Na_2SO_4. It was thus decided to restrain two out of three O–H distances to 0.96(2) Å; the third proton was attributed to pyrrole, with the N–H distance fixed at 0.88(2) Å. Such a presentation of the crystal structure gives the best

explanation of the chemical properties demonstrated by the aforementioned empirical results. The double positive charge of **$H_2O{\subset}$2-Zn$_2$** is equalised by tetrakis(trifluoroacetato)zincate(II) anion. Some of the trifluoromethyl groups were disordered over two positions. Some of the $CHCl_3$ molecules were disordered over two positions. Selected C–Cl distances were adequately restrained to 1.77(2) or 1.770(2) Å. Similarly, the 1,3-Cl–Cl distances were fixed to 2.90(4) or 2.900(2) Å. The structure was deposited in CCDC with deposition number #2452188.

Cage 3-Zn$_{12}$

Crystals suitable for SCXRD analysis were grown *via* slow evaporation of $CHCl_3$ solution. The NH protons were located on the difference Fourier map and refined freely without geometric constraints. Virtually all 35 $CHCl_3$ molecules were disordered and were modelled accordingly: C–Cl distance restraints of 1.77 Å with an adequate standard deviation were applied. Analogously, 1,3-Cl–Cl distances were restrained to 2.92 Å, with an adequate standard deviation. The structure was deposited in CCDC with deposition number #2452189.

Cage 4-Zn$_4$

Crystals suitable for SCXRD analysis were grown *via* slow evaporation of pyridine solution. One of the *tren* moieties, located at the face of the tetrahedral cage, was disordered. The N_{tren}–$C_{\alpha\text{-}tren}$ distances were restrained to 1.413(2) Å. One imine group was also involved in this disorder, and the $C_{\alpha\text{-pyrrole}}$–$N_{imine}$ distances were restrained to 1.280(2) Å. This part of the molecule was excluded from the bond length analysis conducted in the manuscript. All pyridine molecules in the crystal structure were disordered. Due to the inability to locate charge-compensating cations within the crystal structure of **4-Zn$_4$**, it was arbitrarily assumed that the organic ligand undergoes partial tautomerisation. This assumption is justified by literature precedent.[65] Furthermore, the proposed structure is supported by high-resolution ESI-MS data, which revealed a dominant signal at *m/z* consistent with the elemental composition $C_{120}H_{150}N_{44}Zn_4$. The structure was deposited in CCDC with deposition number #2452190.

Synthesis

$CHCl_3$ for synthesis and $CDCl_3$ were prepared directly before use by running through a basic alumina column. Reagents not listed here were used as received.

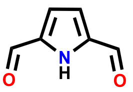

2,5-Diformylpyrrole was synthesised as described in the literature.[92]

Cage [1-Zn$_2$]OAc

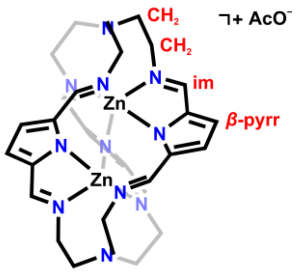

2,5-Diformylpyrrole (24.6 mg, 0.2 mmol) was placed in a 25 mL round-bottom flask equipped with a magnetic stirrer and dissolved in 5 mL of *n*-butanol. Then DIPEA (50 µL, 0.29 mmol) was added, and the mixture was stirred for 5 minutes. After this time, zinc(II) acetate dihydrate (58.5 mg, 0.27 mmol) was added, and the mixture was sonicated for 1 minute. Subsequently, tris(2-aminoethyl)amine (20 µL, 0.13 mmol) and 5 mL of *n*-butanol were added. Flask was equipped with a reflux condenser, and the mixture was stirred under reflux for 17 hours. After completion of the reaction, the solution was concentrated to ca. 2 mL under reduced pressure. Et_2O was added in excess, inducing precipitation of the product **[1-Zn$_2$]OAc**. The suspension was transferred to a centrifuge tube and centrifuged for 5 minutes (6000 rpm). The supernatant was separated from the solid, and the washing procedure with Et_2O was repeated twice. The precipitate was then treated with $CHCl_3$ and centrifuged again for 5 minutes (6000 rpm) to purify **[1-Zn$_2$]OAc** further. Then the $CHCl_3$ solution was separated from the solid, and the procedure was repeated twice. Finally, it was concentrated using a rotary evaporator, yielding a ruby-red solid. The product was dried under vacuum for 1 hour. Yield: 47.1 mg (95%).

^{1}H NMR (600 MHz, $CDCl_3$, 300 K) δ (ppm): 7.99 (s, 6H, im), 6.61 (s, 6H, *b*-pyrr), 3.10 – 2.95 (m, 18H, CH_2), 2.53 – 2.44 (m, 6H, CH_2), 1.94 (s, 3H, C**H$_3$**COO).
^{13}C NMR (151 MHz, $CDCl_3$, 300 K) δ (ppm): 179.3 (CH_3**C**OO), 160.2 (im), 141.5 (*a*-pyrr), 119.0 (*b*-pyrr), 58.1 (CH_2), 55.1 (CH_2), 23.3 (**C**H_3COO).
HR-MS (ESI+, TOF): *m/z* 680.1721 calcd. for $[C_{30}H_{36}N_{11}Zn_2]^+$ 680.1706.

Cage [1-Zn_2]CF_3COO

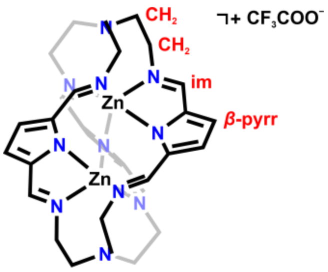

2,5-Diformylpyrrole (12.3 mg, 0.1 mmol) was placed in a 25 mL round-bottom flask equipped with a magnetic stirrer and dissolved in 5 mL of $CHCl_3$. Then DIPEA (25 µL, 0.15 mmol) was added, and the mixture was stirred for 5 minutes. After this time, zinc(**II**) trifluoroacetate hydrate (38.9 mg, 0.13 mmol) was added, and the mixture was sonicated for 1 minute. Subsequently, tris(2-aminoethyl)amine (10 µL, 0.67 mmol) and 5 mL of $CHCl_3$ were added. Flask was equipped with a reflux condenser, and the mixture was stirred under reflux for 17 hours. After completion of the reaction, the solution was concentrated to ca. 2 mL under reduced pressure. Cyclohexane was added in excess, inducing precipitation of the product **[1-Zn_2]CF_3COO**. The suspension was transferred to a centrifuge tube and centrifuged for 5 minutes (6000 rpm). The supernatant was separated from the solid, and the washing procedure with cyclohexane was repeated twice. The precipitate was then treated with $CHCl_3$ and centrifuged again for 5 minutes (6000 rpm) to purify **[1-Zn_2]CF_3COO** further. Then the $CHCl_3$ solution was separated from the solid, and the procedure was repeated twice. Finally, it was concentrated using a rotary evaporator, yielding a ruby-red solid. The product was dried under vacuum for 1 hour. Yield: 20.1 mg (77%).

^{1}H NMR (600 MHz, CD_3CN, 300 K) δ (ppm): 8.08(s, 6H, im), 6.66 (s, 6H, *b*-pyrr), 3.04 – 2.95 (m, 18H, CH_2), 2.60 – 2.52 (m, 6H, CH_2).
^{13}C NMR (151 MHz, CD_3CN, 300 K) δ (ppm): 161.5 (im), 160.9 (CF_3**C**OO), 142.6 (*a*-pyrr), 119.5 (*b*-pyrr), 58.5 (CH_2), 55.6 (CH_2).
^{19}F NMR (465 MHz, CD_3CN, 300 K) δ (ppm): -75.1 (C**F**$_3$COO).
HR-MS (ESI+, TOF): *m/z* 680.1728 calcd. for $[C_{30}H_{36}N_{11}Zn_2]^+$ 680.1706.

Cage H_2O⊂2-Zn_2

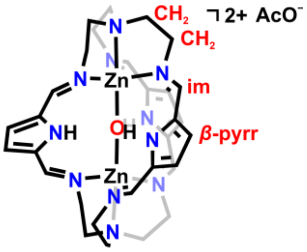

Cage [1-Zn_2]OAc (2 mg, 0.003 mmol) was placed in a NMR tube and dissolved in 500 µL of CD_3CN. Gradual addition of 800 µL deionised water in portions of 100 µL led to the immediate transformation of **[1-Zn_2]OAc** into **H_2O⊂2-Zn_2**.

^{1}H NMR (600 MHz, CD_3CN, 300 K) δ (ppm): 17.98 (s, 3H, NH/H_2O), 8.42 (s, 6H, im), 6.85 (s, 6H, *b*-pyrr), 3.44 (td, ^3J = 12.7 Hz, ^4J = 2.7 Hz, 6H, CH_2), 3.35 (dd, ^2J = 12.3 Hz, ^3J = 3.3 Hz, 6H, CH_2), 3.09 (dd, ^2J = 13.7 Hz, ^3J = 2.7 Hz, 6H, CH_2), 2.73 (td, ^3J = 13.3 Hz, ^4J = 3.7 Hz, 6H, CH_2), 1.79 (s, 6H, C**H_3**COO).

Cage 3-Zn_{12}

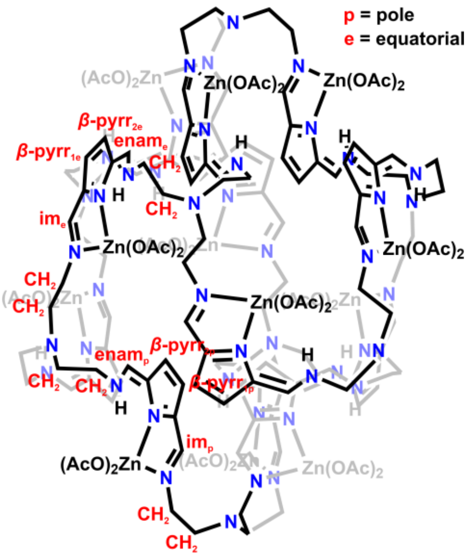

2,5-Diformylpyrrole (24.6 mg, 0.2 mmol) was placed in a 25 mL round-bottom flask equipped with a magnetic stirrer. $CHCl_3$ (5 mL) was added to dissolve the solid, followed by DIPEA (50 µL, 0.29 mmol), and the solution was stirred for 5 minutes. After this time, zinc(II) acetate dihydrate (58.5 mg, 0.27 mmol) was added, and the mixture was sonicated for 1 minute. Next, tris(2-aminoethyl)amine (20 µL, 0.13 mmol) and an additional 5 mL of $CHCl_3$ were added to the reaction mixture. Flask was equipped with a reflux condenser, and the mixture was stirred under reflux for 17 hours. After completion of the reaction, the solution was concentrated to ca. 2 mL under reduced pressure. MeCN was added in excess, inducing precipitation of the product **3-Zn_{12}**. The suspension was transferred to a centrifuge tube and centrifuged for 5 minutes (6000 rpm). The supernatant was separated from the solid, and the MeCN washing procedure was repeated twice. The precipitate was dissolved in $CHCl_3$, and the resulting solution was removed using a rotary evaporator. The obtained, slightly yellowish solid was dried under vacuum for 1 hour. Yield: 49.1 mg (67%).

^{1}H NMR (600 MHz, $CDCl_3$, 300 K) δ (ppm): 11.71 (m, 6H, NH_p), 11.31 (m, 6H, NH_e), 8.35 (d, ^3J = 15.6 Hz, 6H, $enam_p$), 7.99 (s, 6H, im_e), 7.64 (s, 6H, im_p), 7.38 (d, ^3J = 15.1 Hz, 6H, $enam_e$), 6.24 (d, ^3J = 3.8 Hz, 6H, *b*-$pyrr_{2p}$), 6.19 (d, ^3J = 3.8 Hz, 6H, *b*-$pyrr_{1e}$), 5.81 (d, ^3J = 3.8 Hz, 6H, *b*-$pyrr_{2e}$), 4.50 (m, 6H, CH_2), 4.34 (m, 6H, CH_2), 4.25 (d, ^3J = 3.8 Hz, 6H, *b*-$pyrr_{1p}$), 4.14 – 3.85 (m, 24H, CH_2), 3.84 – 3.73 (m, 12H, CH_2), 3.48 – 3.35 (m, 12H, CH_2), 3.21 (m, 6H, CH_2), 2.88 – 2.74 (m, 12H, CH_2), 2.64 (m, 6H, CH_2), 2.55 (m, 6H, CH_2), 2.36 (m, 6H, CH_2), 2.00 (s, 18H, C**H_3**COO), 1.34 (s, 18H, C**H_3**COO).
^{13}C NMR (151 MHz, $CDCl_3$, 300 K) δ (ppm): 179.4 (CH_3**C**OO), 178.5 (CH_3**C**OO), 160.6 (im_p), 159.3 (im_e), 158.9 ($enam_p$), 157.8 ($enam_e$), 147.4 (*a*-$pyrr_e$), 146.3 (*a*-$pyrr_p$), 132.9 (*a*-$pyrr_e$), 132.3 (*a*-$pyrr_p$), 129.9 (*b*-$pyrr_{2p}$), 128.8 (*b*-$pyrr_{2e}$), 117.9 (*b*-$pyrr_{1p}$), 115.4 (*b*-$pyrr_{1e}$), 58.3 (CH_2), 54.3 (CH_2), 53.5 (CH_2), 53.4 (CH_2), 53.0 (CH_2), 51.6 (CH_2), 49.3 (CH_2), 45.1 (CH_2), 23.2 (**C**H_3COO), 21.9 (**C**H_3COO).

Cage 4-Zn_4

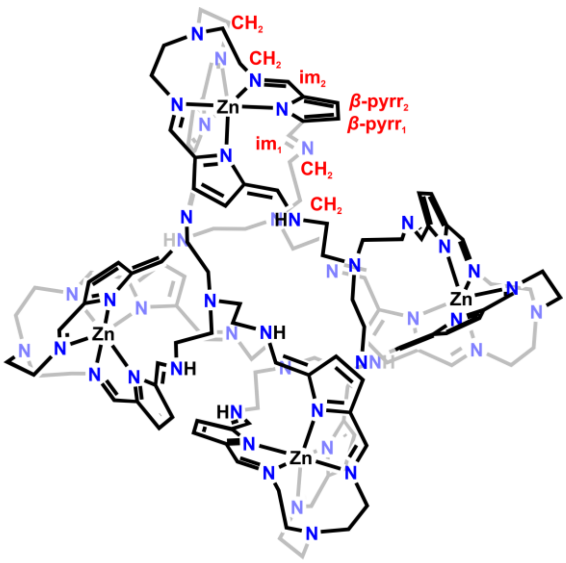

3-Zn_{12} (44.2 mg, 0.01 mmol) was placed in a 10 mL round-bottom flask and dissolved in 3 mL of pyridine. The solution was left to stand for 20 hours, allowing for slight evaporation. This led to the formation of **4-Zn_4** as yellow crystals, which were separated from the solvent and washed with MeCN. The obtained product was dried under vacuum for 1 hour. Yield: 17.7 mg (72%).

^{1}H NMR (600 MHz, CD_3OD, 300 K) δ (ppm): 8.21 (s, 12H, im$_2$, 7.11 (d, ^3J = 3.8 Hz, 12H, *b*-pyrr$_2$), 6.75 (d, ^3J = 3.8 Hz, 12H, *b*-pyrr$_1$), 6.24 (s, 12H, im$_1$), 3.62 (m, 12H, CH_2), 3.41 (m, 12H, CH_2), 3.21 – 3.16 (m, 24H, CH_2), 2.76 (m, 12H, CH_2), 2.72 – 2.58 (m, 24H, CH_2), 1.91 (m, 12H, CH_2).
^{13}C NMR (151 MHz, CD_3OD, 300 K) δ (ppm): 162.0 (im$_1$), 157.6 (im$_2$), 145.7 (*a*-pyrr$_2$), 137.8 (*a*-pyrr$_1$), 119.0 (*b*-pyrr$_1$), 117.3 (*b*-pyrr$_2$), 59.2 (CH_2), 58.1 (CH_2), 57.2 (CH_2), 55.3 (CH_2).
HR-MS (ESI+, TOF): *m/z* 1234.5113 calcd. for $[C_{120}H_{150}N_{44}Zn_4]^{2+}$ 1234.5139.

Analytical data for [1-Zn_2]OAc

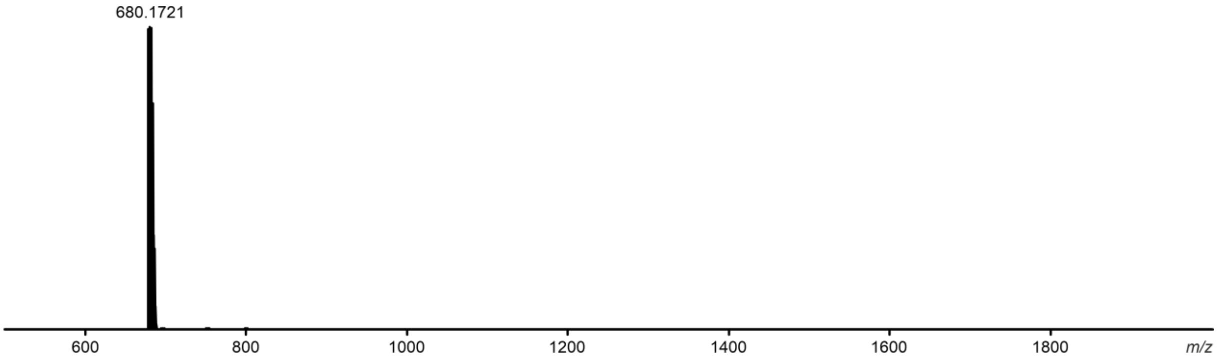

Figure S1. The 500-2000 *m/z* range of the high-resolution mass spectrum of a **[1-Zn_2]OAc** (ESI+, TOF).

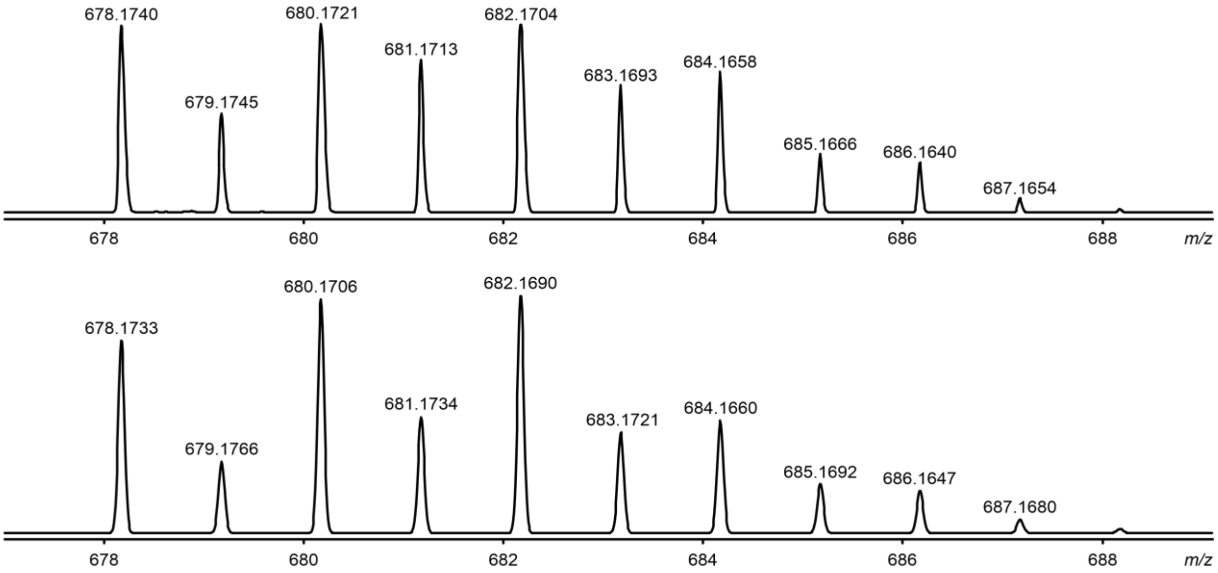

Figure S2. The high-resolution mass spectrum of **[1-Zn_2]OAc** (ESI+, TOF, $[C_{30}H_{36}N_{11}Zn_2]^+$). Top: experimental spectrum, bottom: simulated pattern.

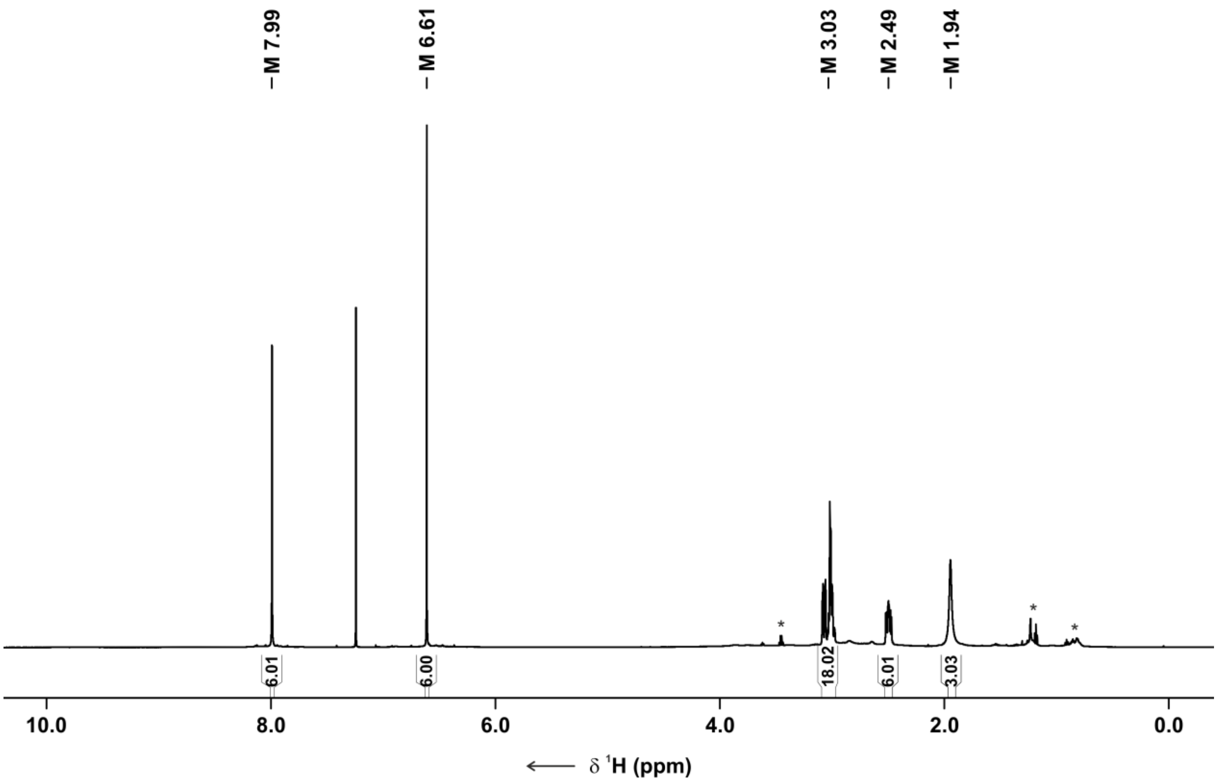

Figure S3. The ^{1}H NMR spectrum of **[1-Zn$_2$]OAc** ($CDCl_3$, 300 K, 600 MHz).

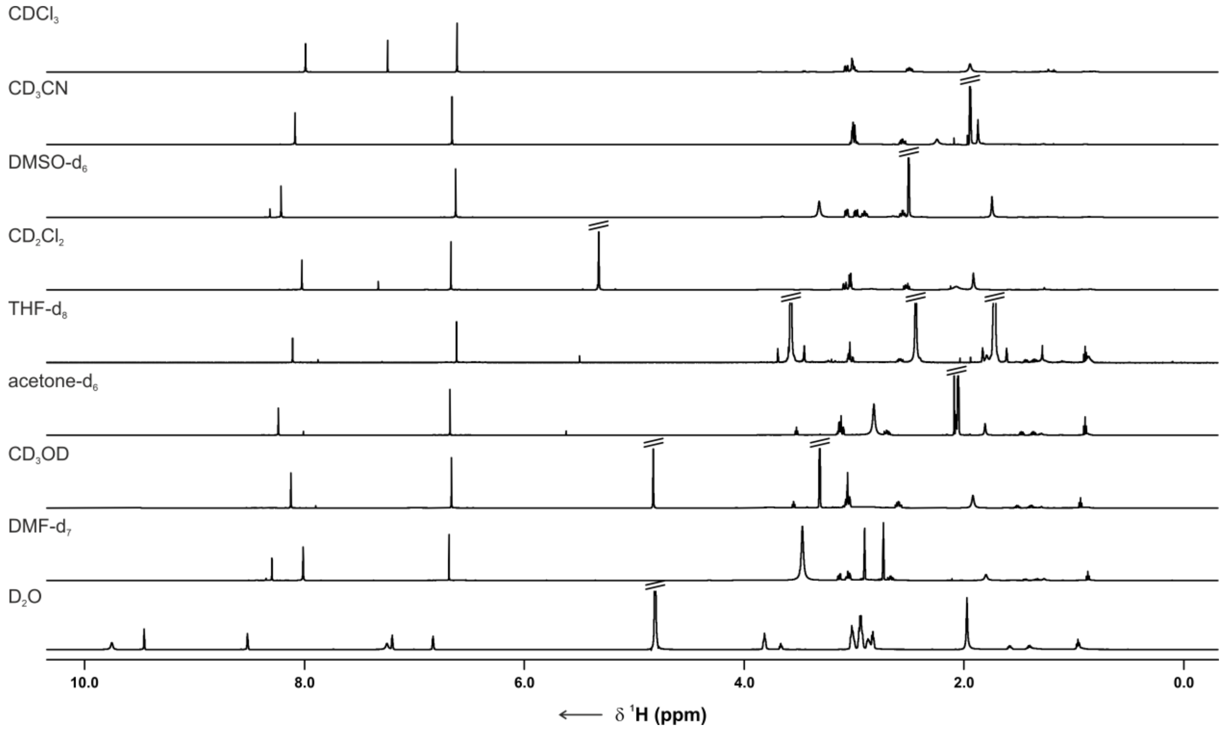

Figure S4. The ^{1}H NMR spectra of **[1-Zn$_2$]OAc** in various deuterated solvents (300 K, 600 MHz).

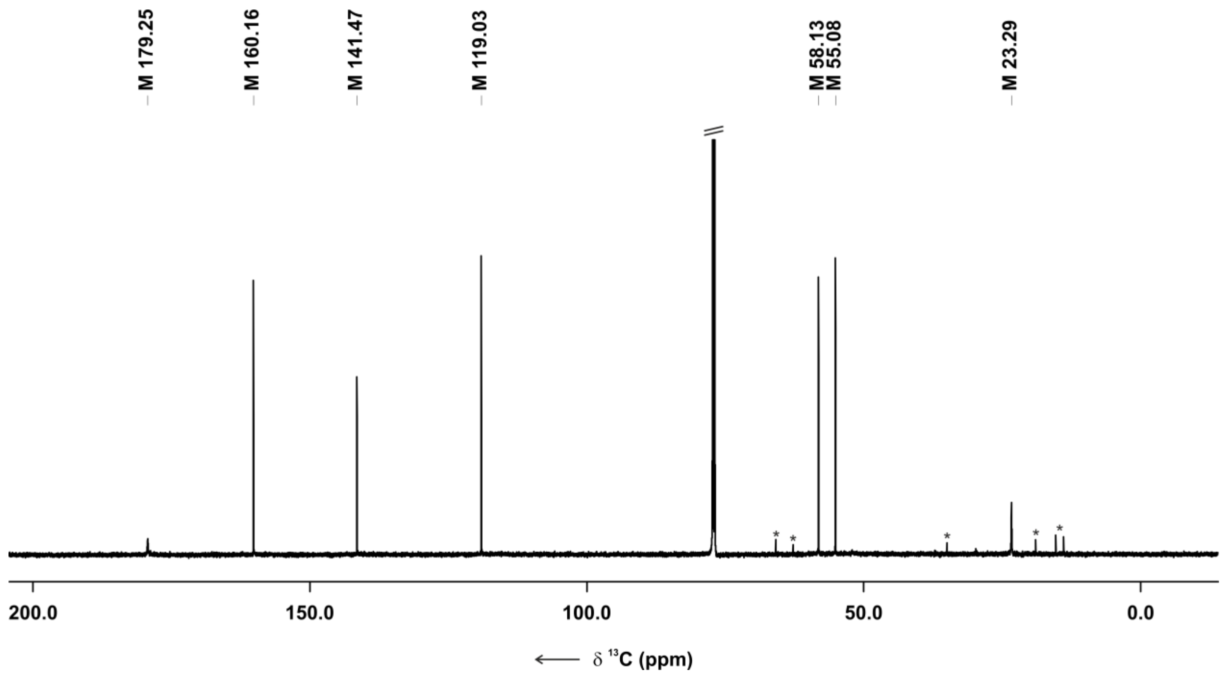

Figure S5. The ^{13}C NMR spectrum of **[1-Zn$_2$]OAc** ($CDCl_3$, 300 K, 151 MHz).

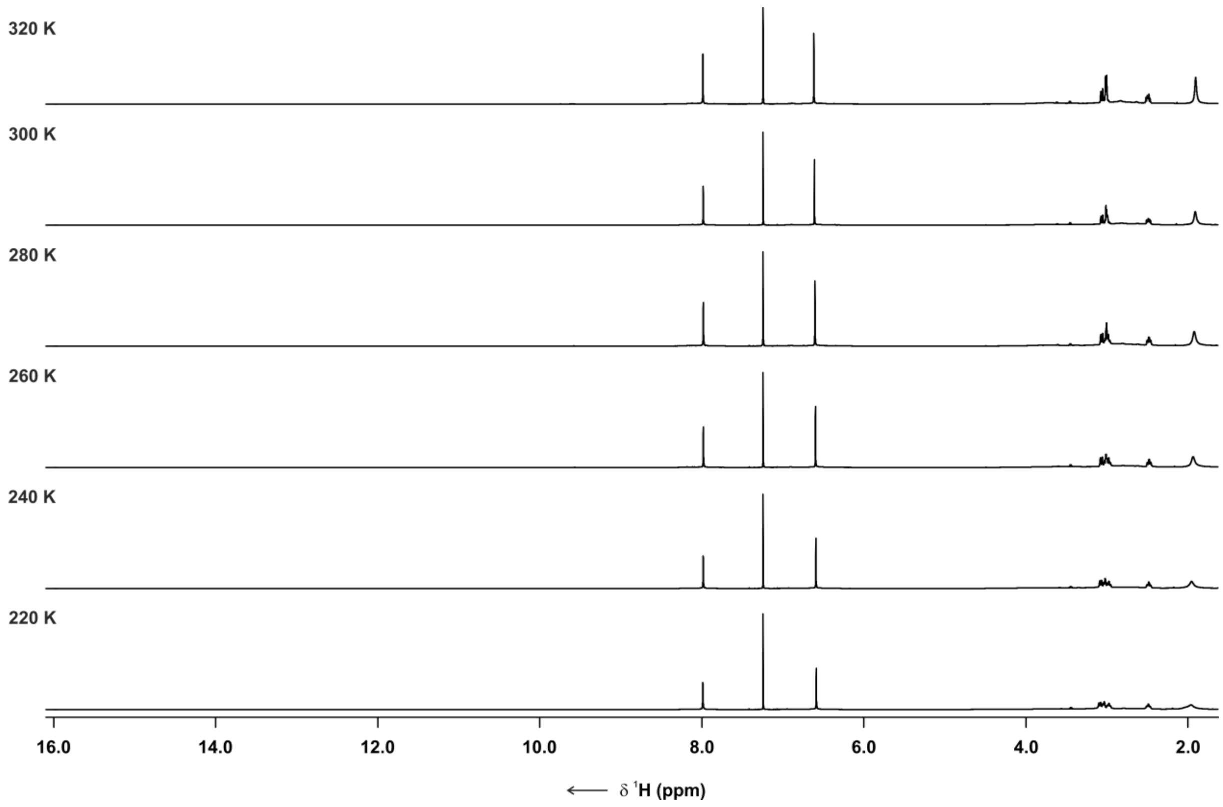

Figure S6. The ^{1}H NMR spectra of **[1-Zn$_2$]OAc** recorded in the 320 K – 220 K temperature range ($CDCl_3$, 600 MHz).

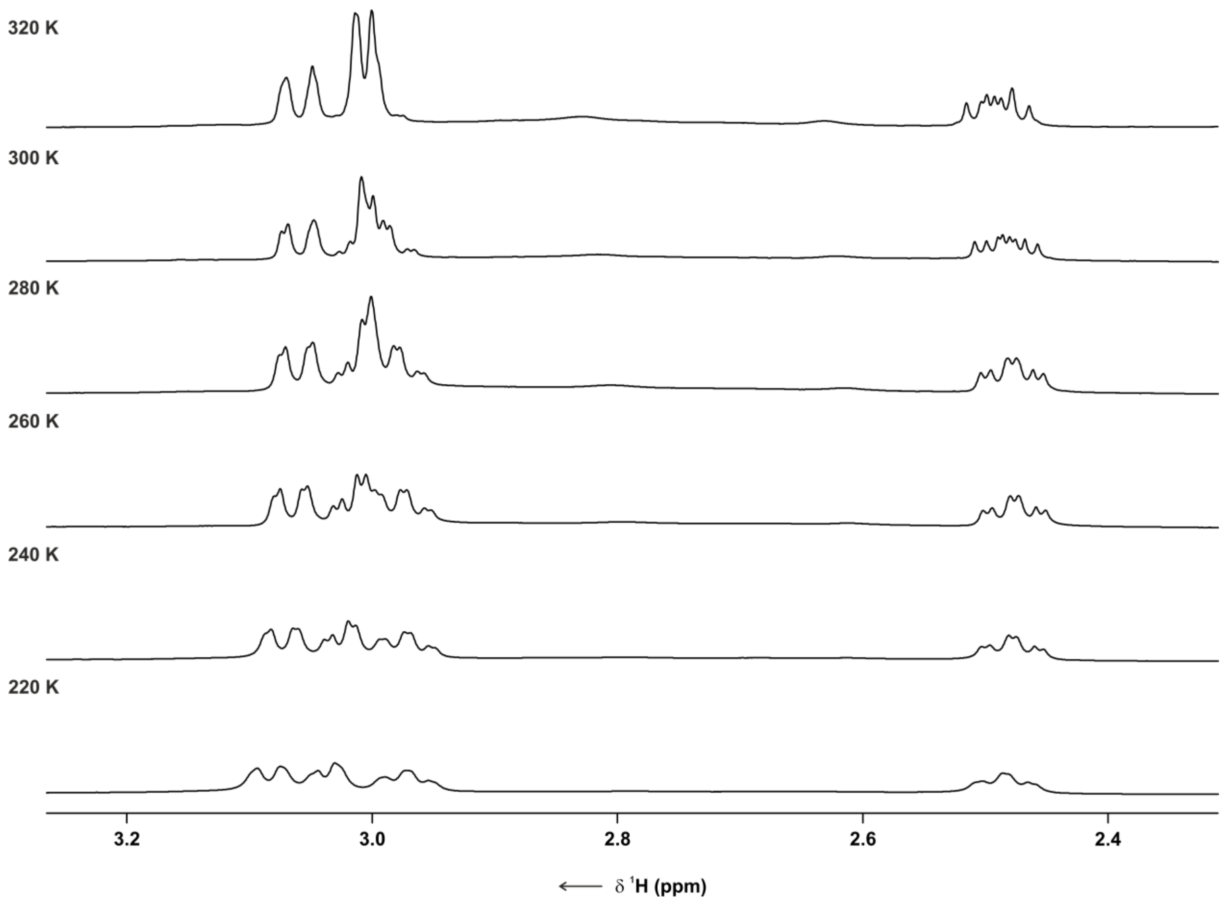

Figure S7. Partial ^{1}H NMR spectra of **[1-Zn$_2$]OAc** recorded in the 320 K – 220 K temperature range ($CDCl_3$, 600 MHz).

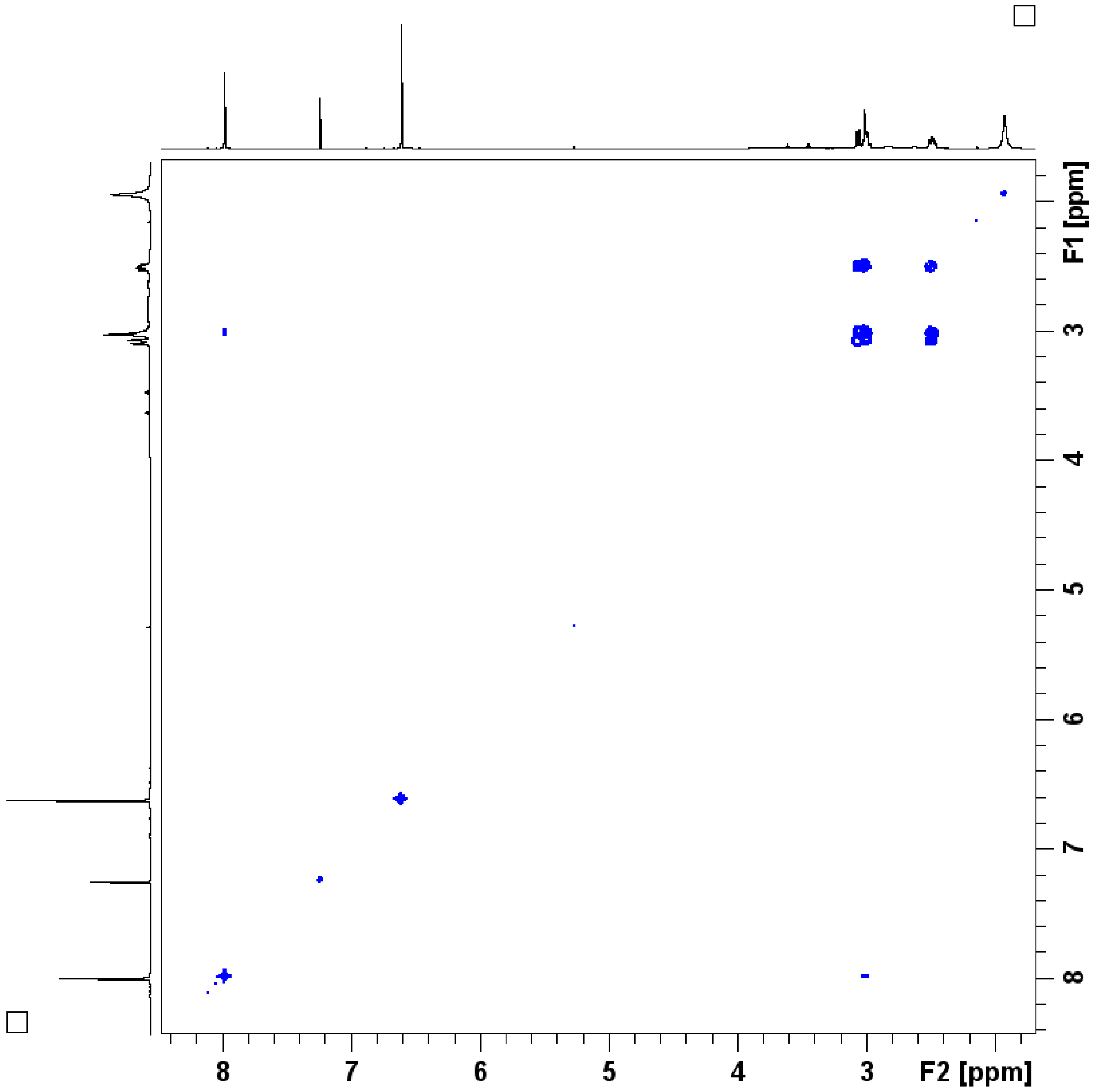

Figure S8. The ^{1}H-^{1}H COSY spectrum of **[1-Zn$_2$]OAc** ($CDCl_3$, 300 K, 600 MHz).

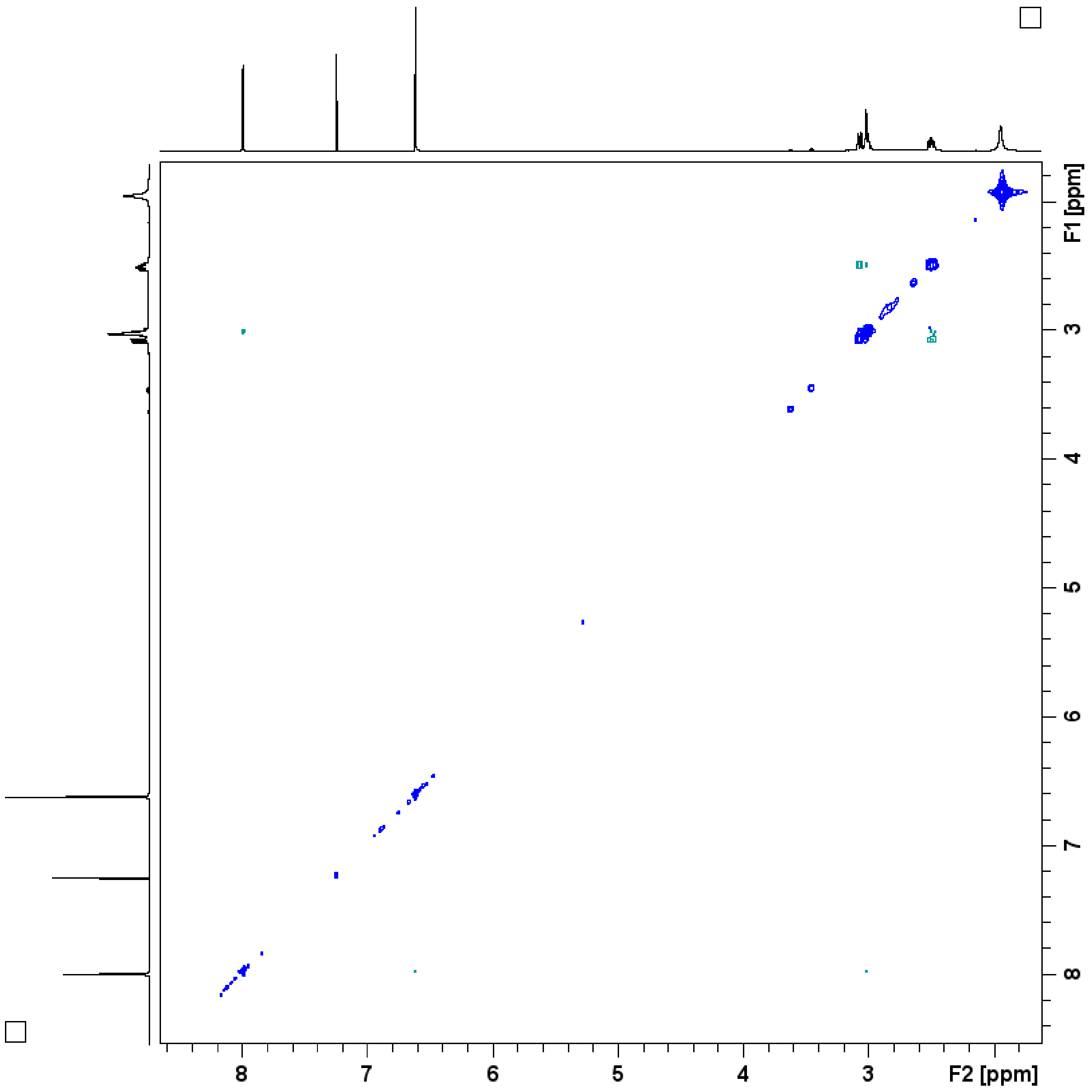

Figure S9. The ^{1}H-^{1}H NOESY spectrum of **[1-Zn$_2$]OAc** ($CDCl_3$, 300 K, 600 MHz).

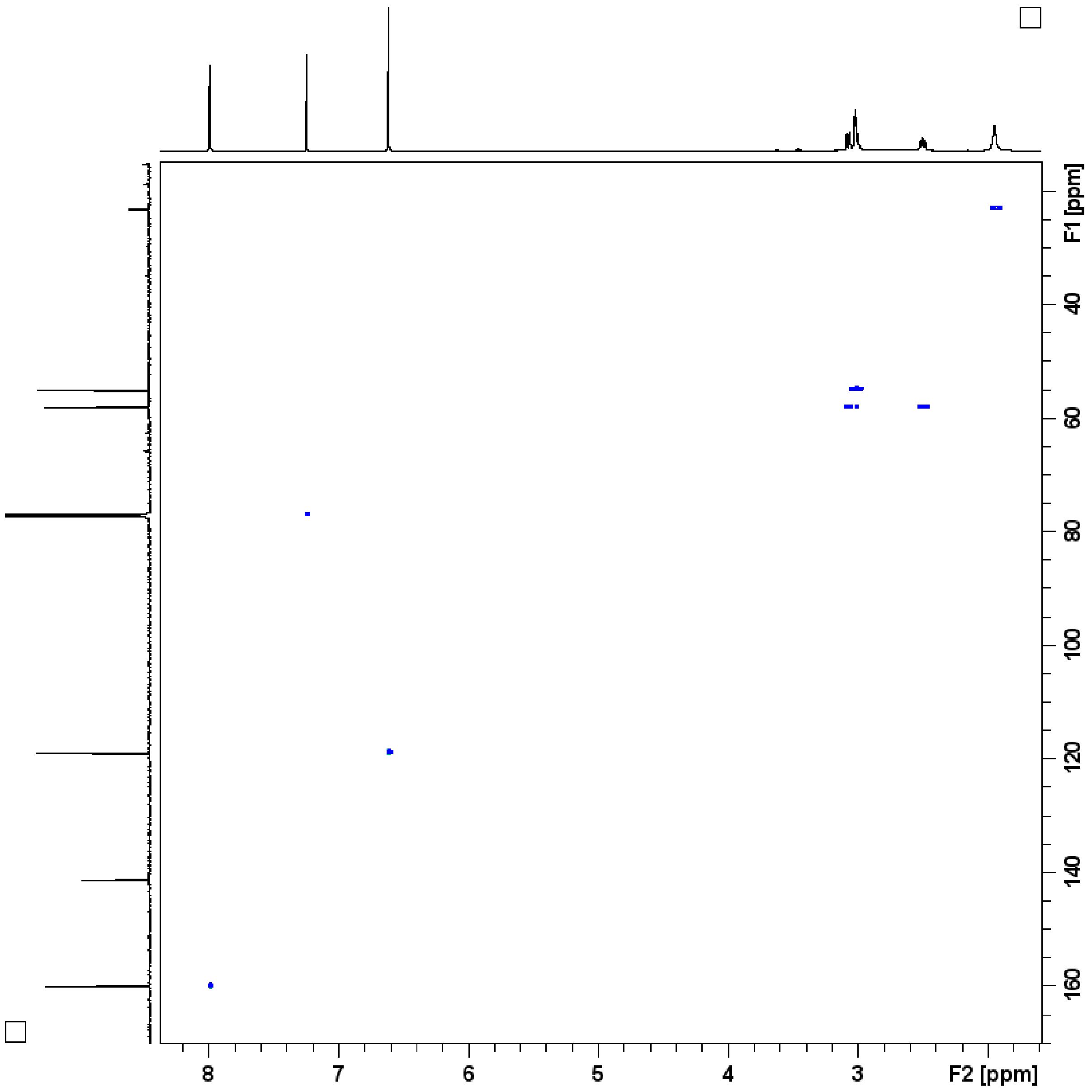

Figure S10. The ^{1}H-^{13}C HSQC spectrum of **[1-Zn$_2$]OAc** ($CDCl_3$, 300 K, 600 MHz).

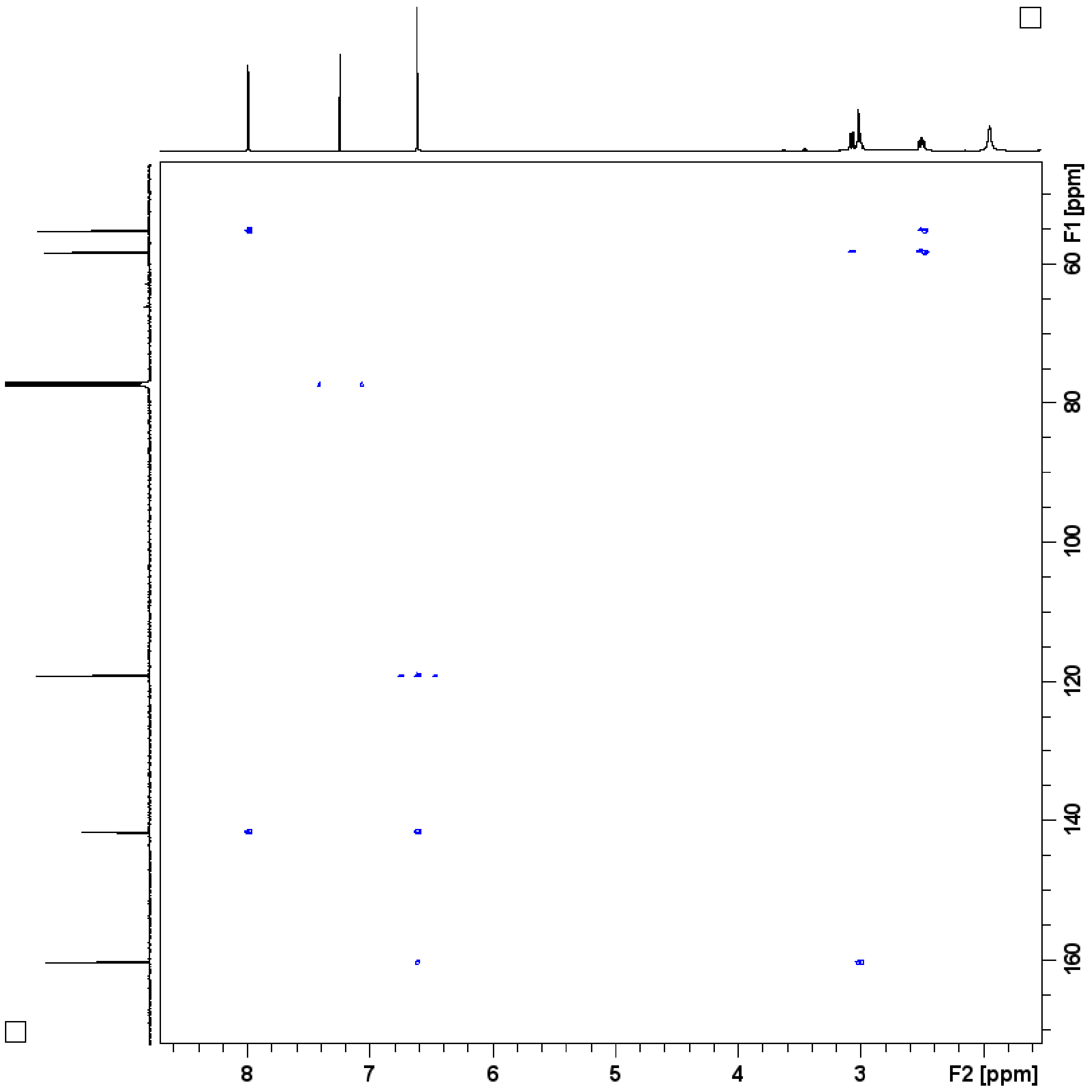

Figure S11. The ^{1}H-^{13}C HMBC spectrum of **[1-Zn$_2$]OAc** ($CDCl_3$, 300 K, 600 MHz).

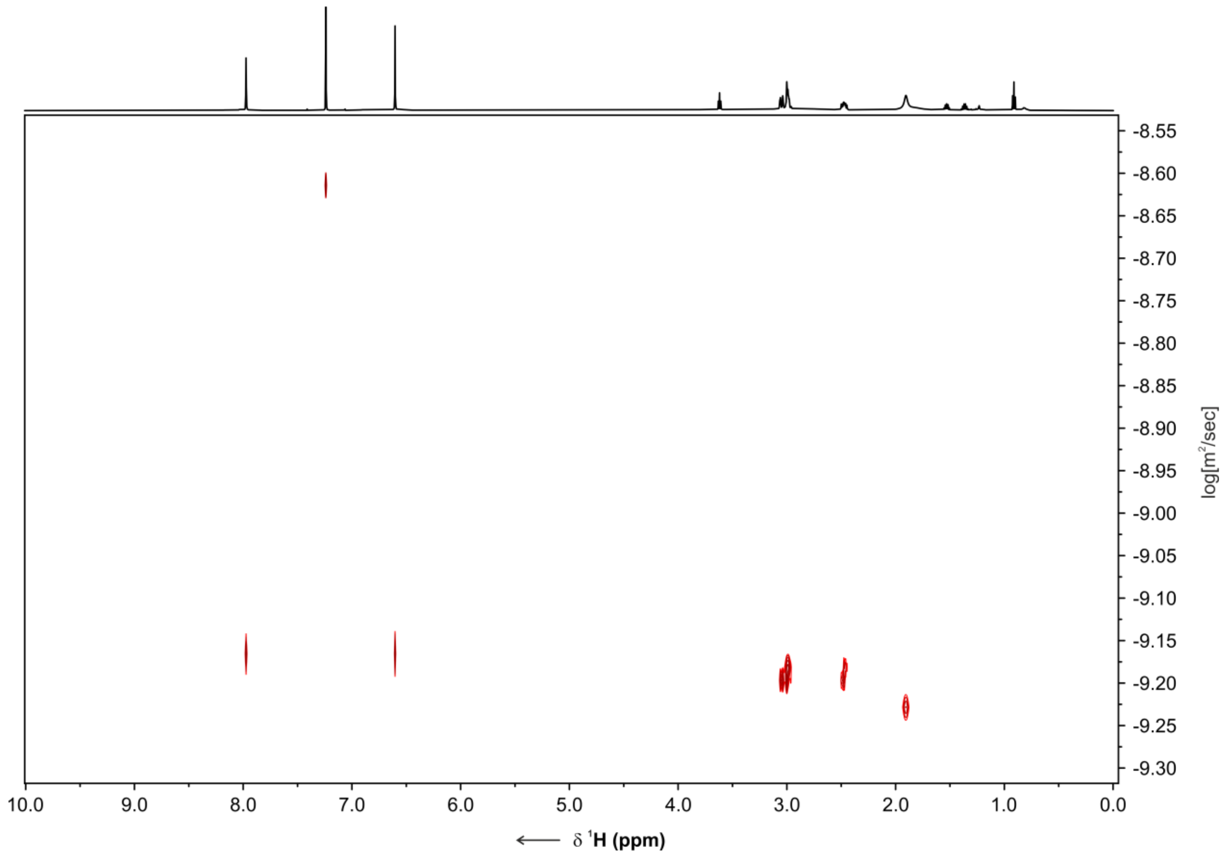

Figure S12. The DOSY NMR spectrum of **[1-Zn$_2$]OAc** ($CDCl_3$, 300 K, 600 MHz).

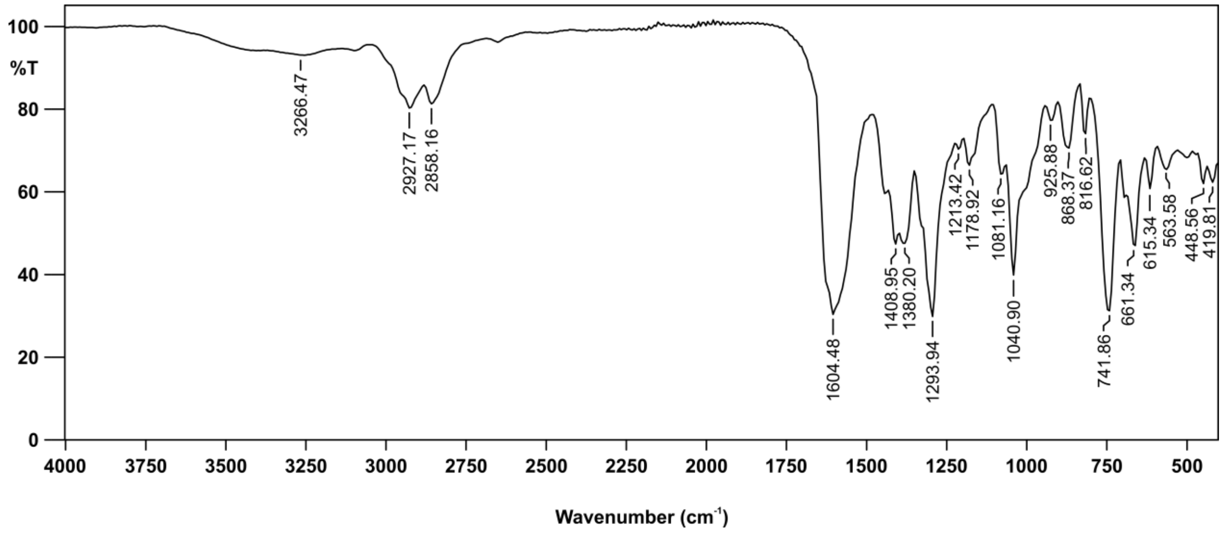

Figure S13. The FT-IR spectrum **[1-Zn$_2$]OAc**.

Analytical data for [1-Zn_2]CF_3COO

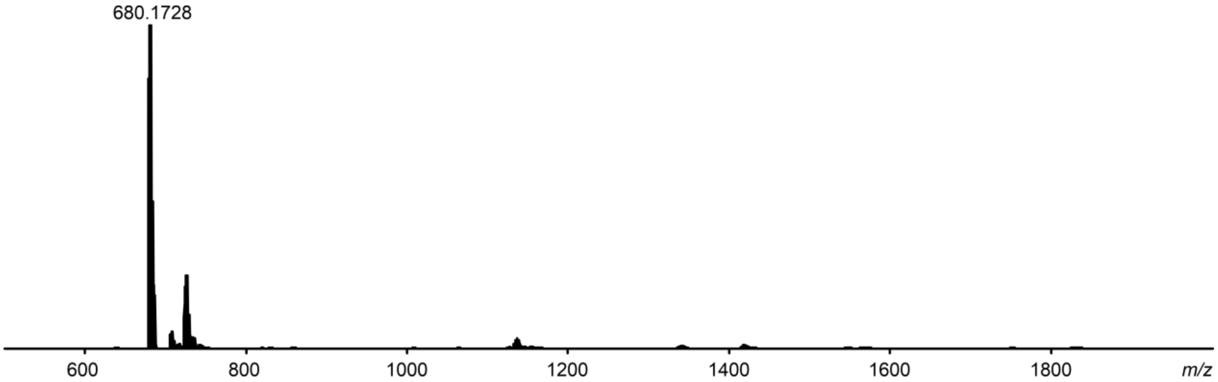

Figure S14. The 500-2000 *m/z* range of the high-resolution mass spectrum of a **[1-Zn_2]CF_3COO** (ESI+, TOF).

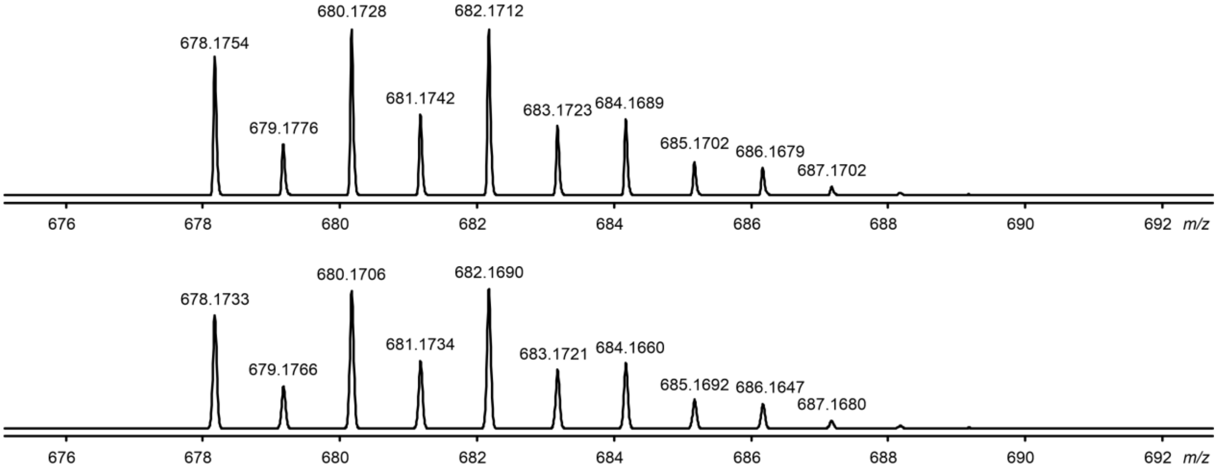

Figure S15. The high-resolution mass spectrum of **[1-Zn_2]CF_3COO** (ESI+, TOF, $[C_{30}H_{36}N_{11}Zn_2]^+$). Top: experimental spectrum, bottom: simulated pattern.

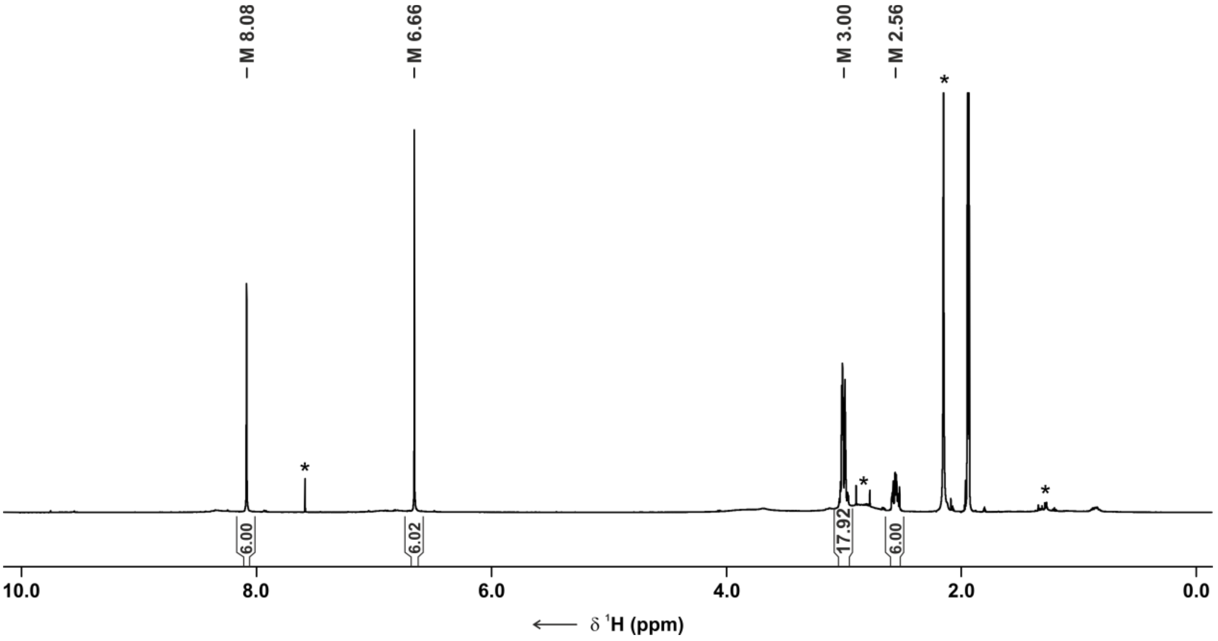

Figure S16. The ^1H NMR spectrum of **[1-Zn_2]CF_3COO** (CD_3CN, 300 K, 600 MHz).

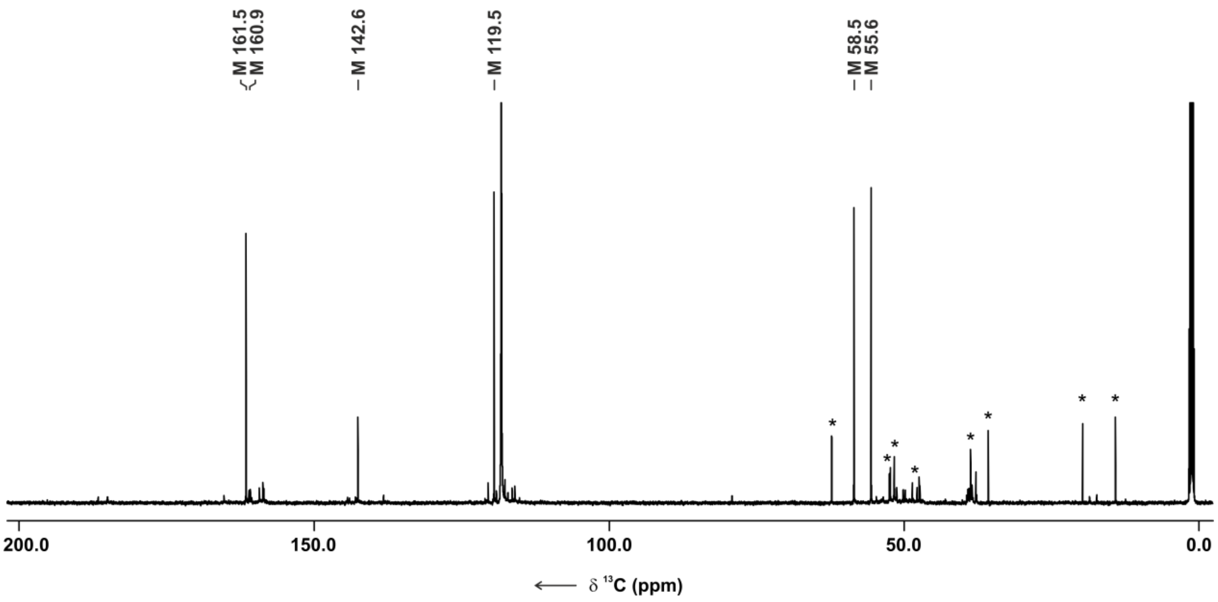

Figure S17. The ^{13}C NMR spectrum of **[1-Zn$_2$]CF$_3$COO** (CD$_3$CN, 300 K, 151 MHz).

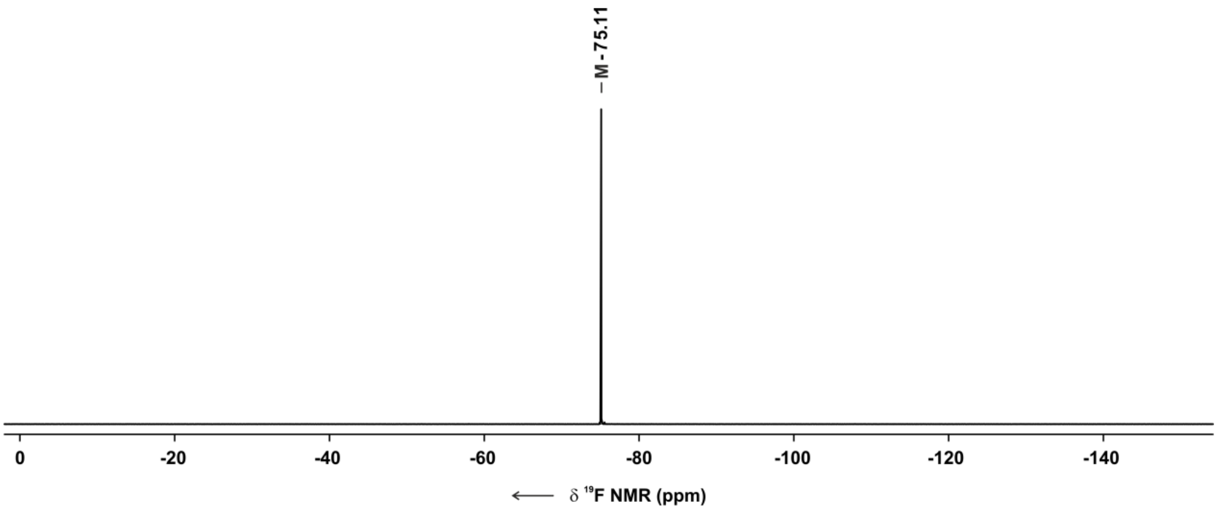

Figure S18. The ^{19}F NMR spectrum of **[1-Zn$_2$]CF$_3$COO** (CD$_3$CN, 300 K, 465 MHz).

Analytical data for $H_2O\subset$2-Zn$_2$

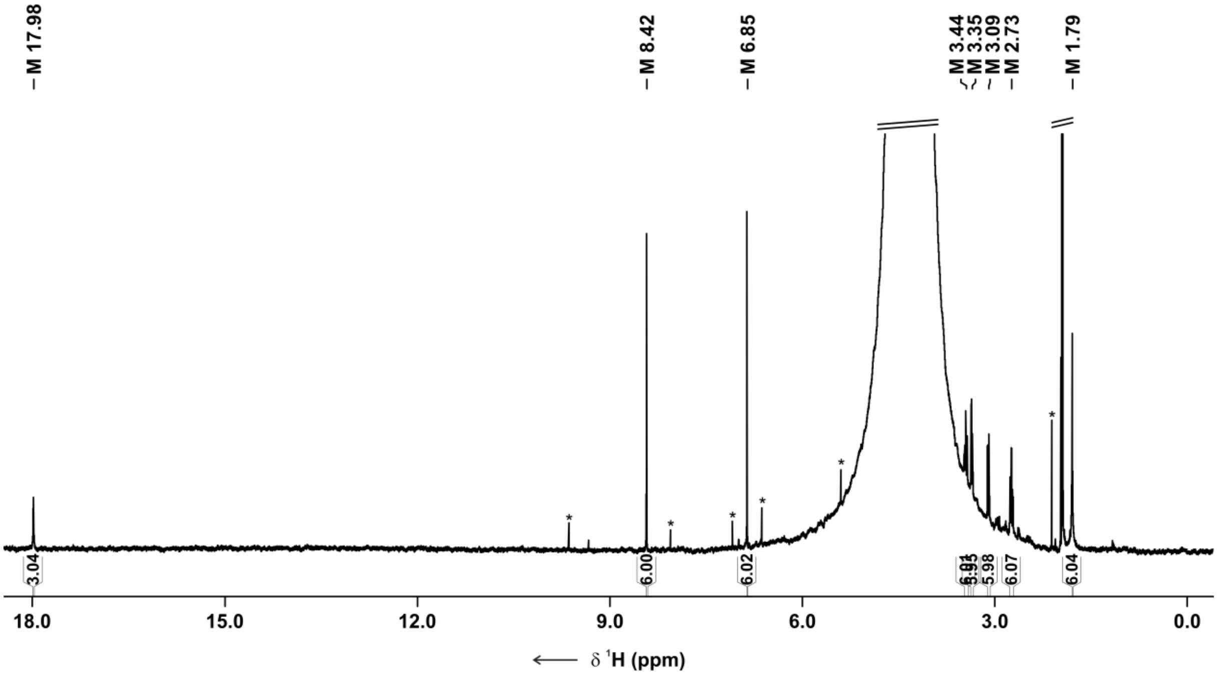

Figure S19. The ^{1}H NMR spectrum of **$H_2O\subset$2-Zn$_2$** (CD_3CN, 300 K, 600 MHz). Signals marked with asterisks originated from residual **[1-Zn$_2$]OAc**, 2,5-diformylpyrrole and tris(2-aminoethyl)amine.

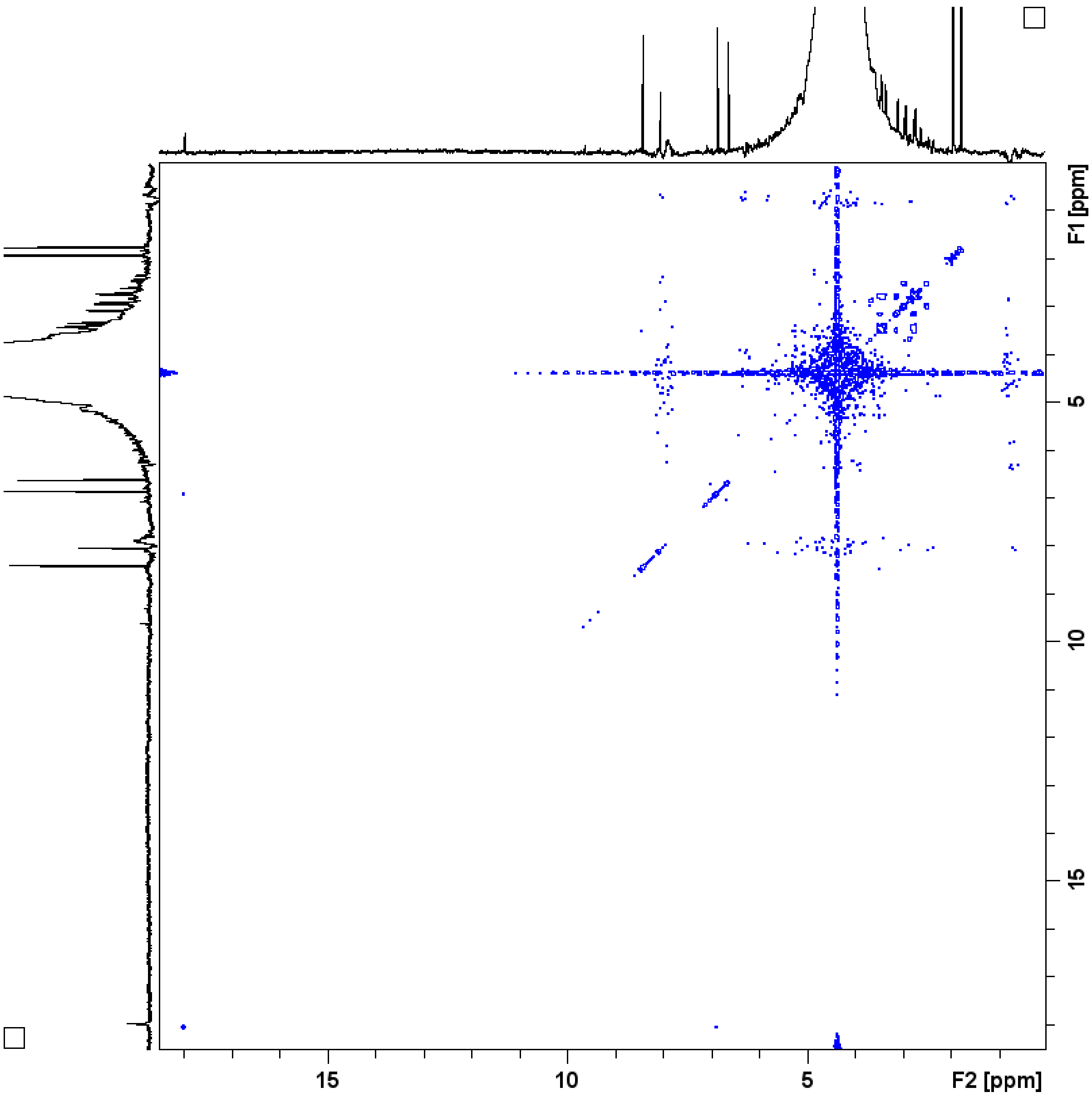

Figure S20. The ^{1}H-^{1}H COSY spectrum of **$H_2O \subset$2-Zn_2** (CD_3CN, 300 K, 600 MHz).

Analytical data for 3-Zn_{12}

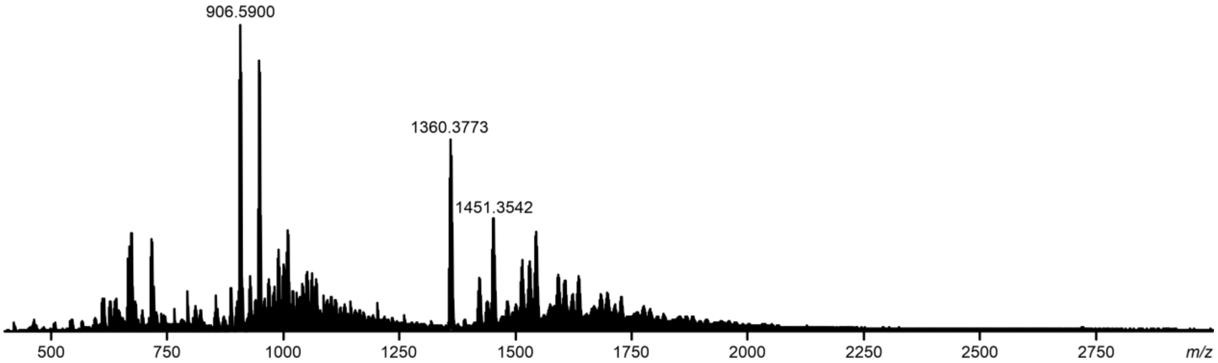

Figure S21. The 400-3000 *m/z* range of the high-resolution mass spectrum of a sample of **3-Zn_{12}** (ESI+, TOF). No signal of **3-Zn_{12}** could be identified.

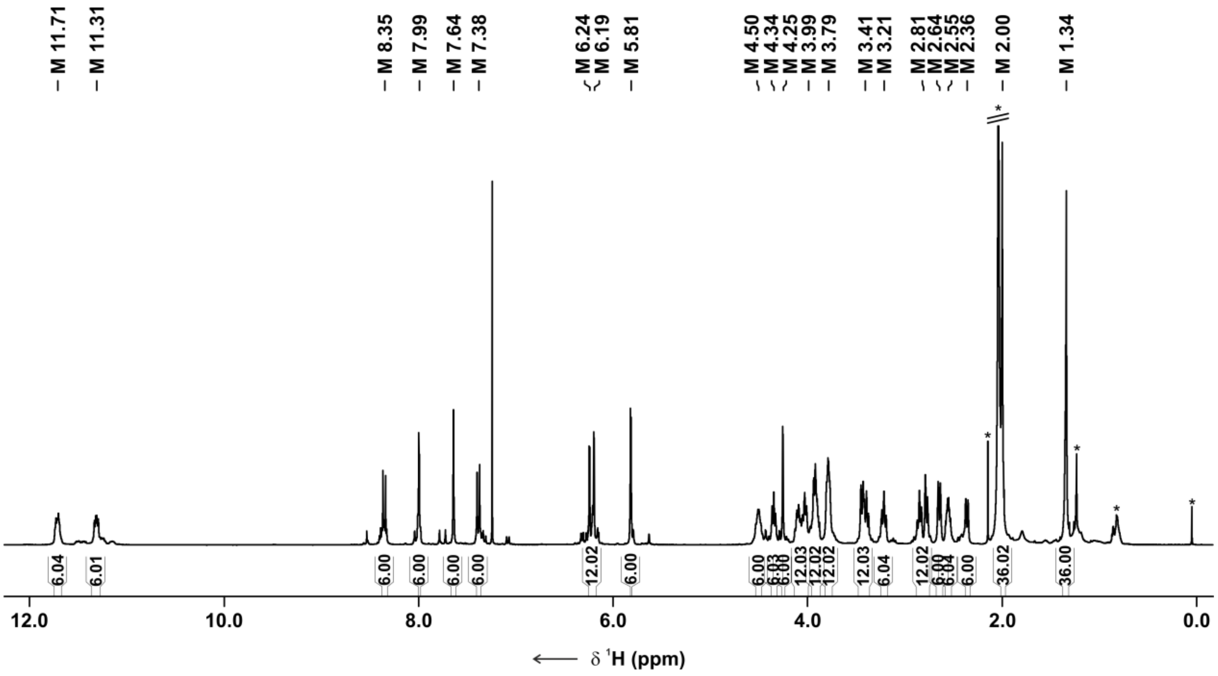

Figure S22. The ^{1}H NMR spectrum of **3-Zn_{12}** ($CDCl_3$, 300 K, 600 MHz).

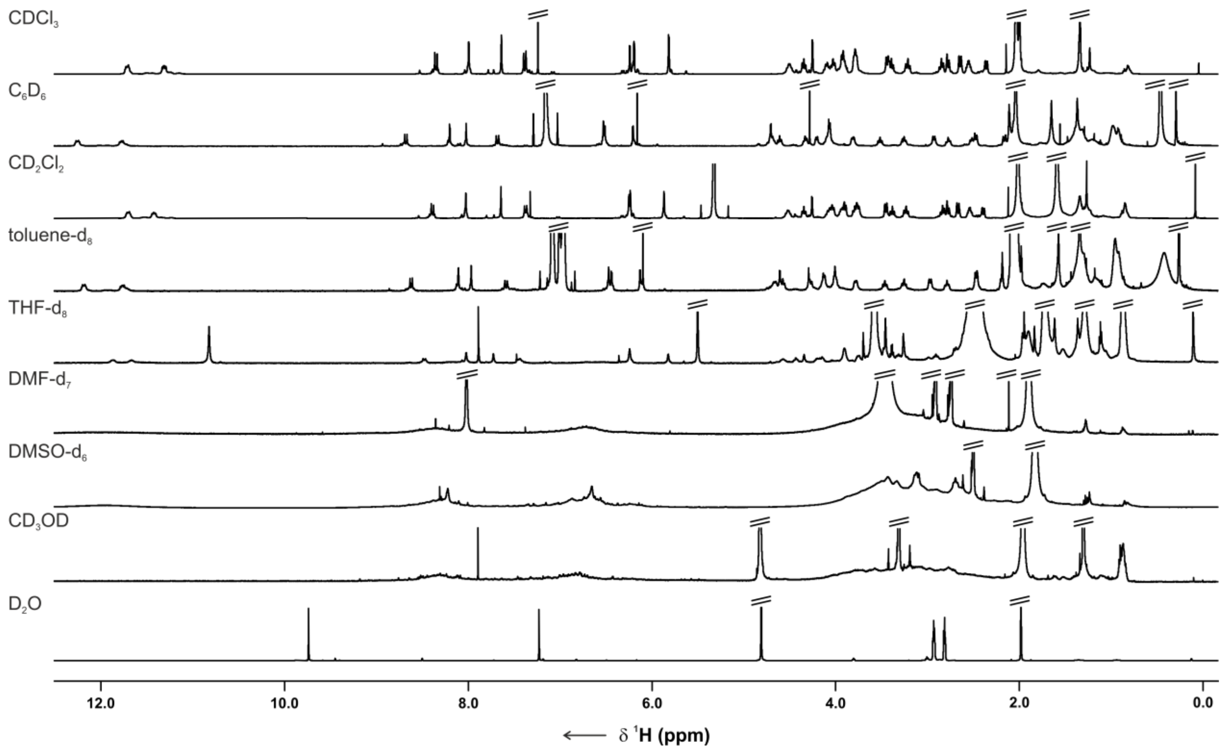

Figure S23. The ^{1}H NMR spectra of **3-Zn$_{12}$** in various deuterated solvents (300 K, 600 MHz).

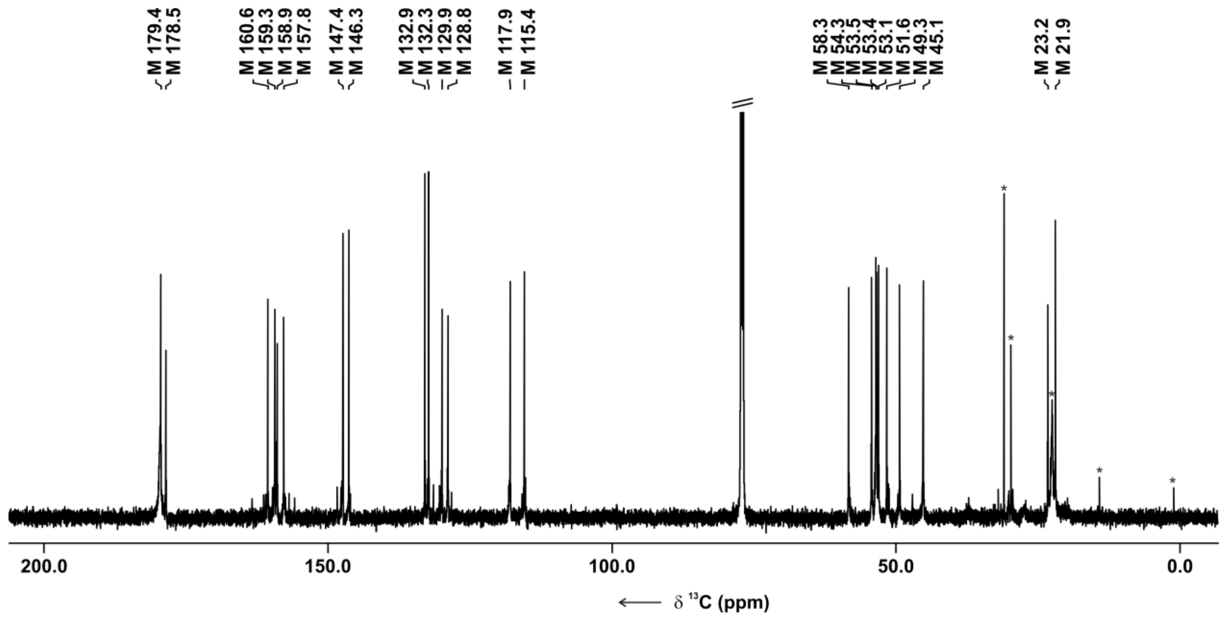

Figure S24. The ^{13}C NMR spectrum of **3-Zn$_{12}$** ($CDCl_3$, 300 K, 151 MHz).

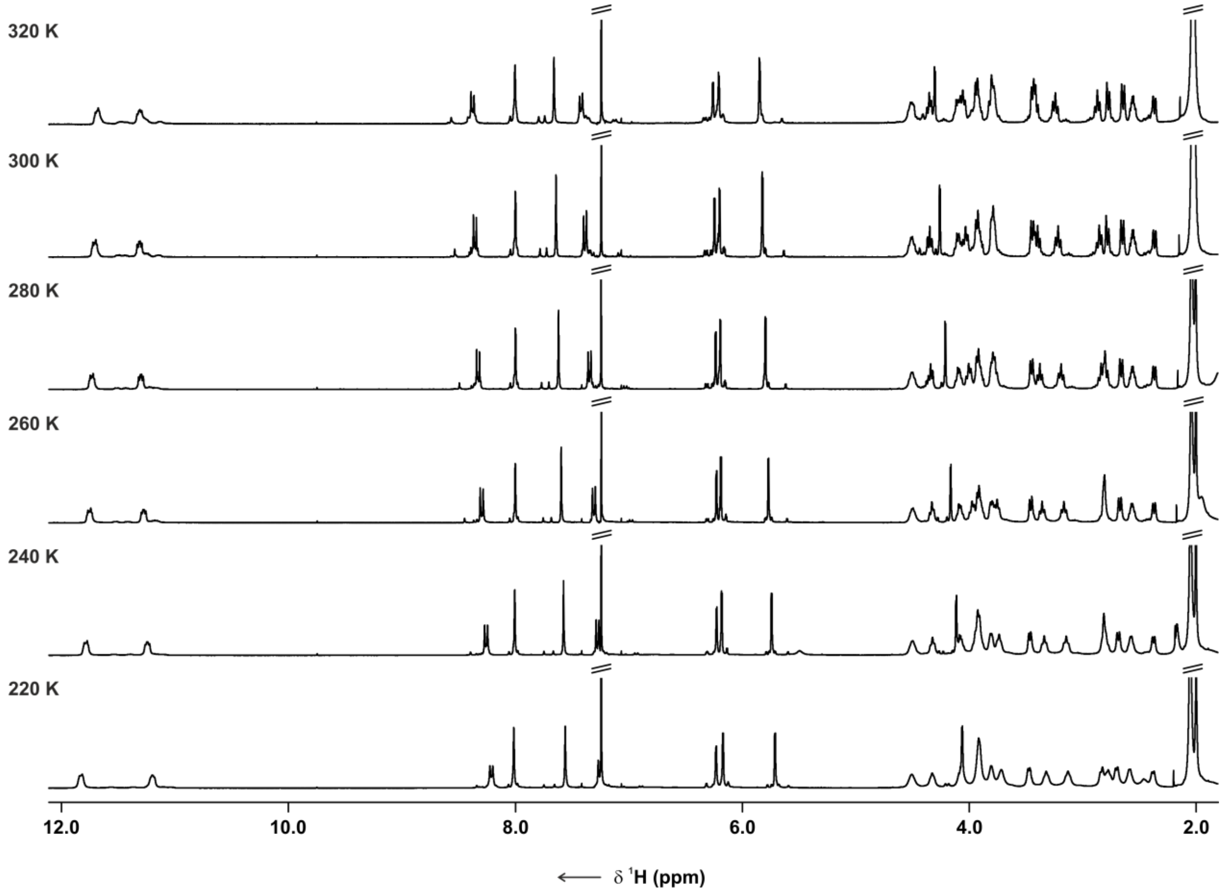

Figure S25. The ¹H NMR spectra of **3-Zn₁₂** recorded in the 320 K – 220 K temperature range ($CDCl_3$, 600 MHz).

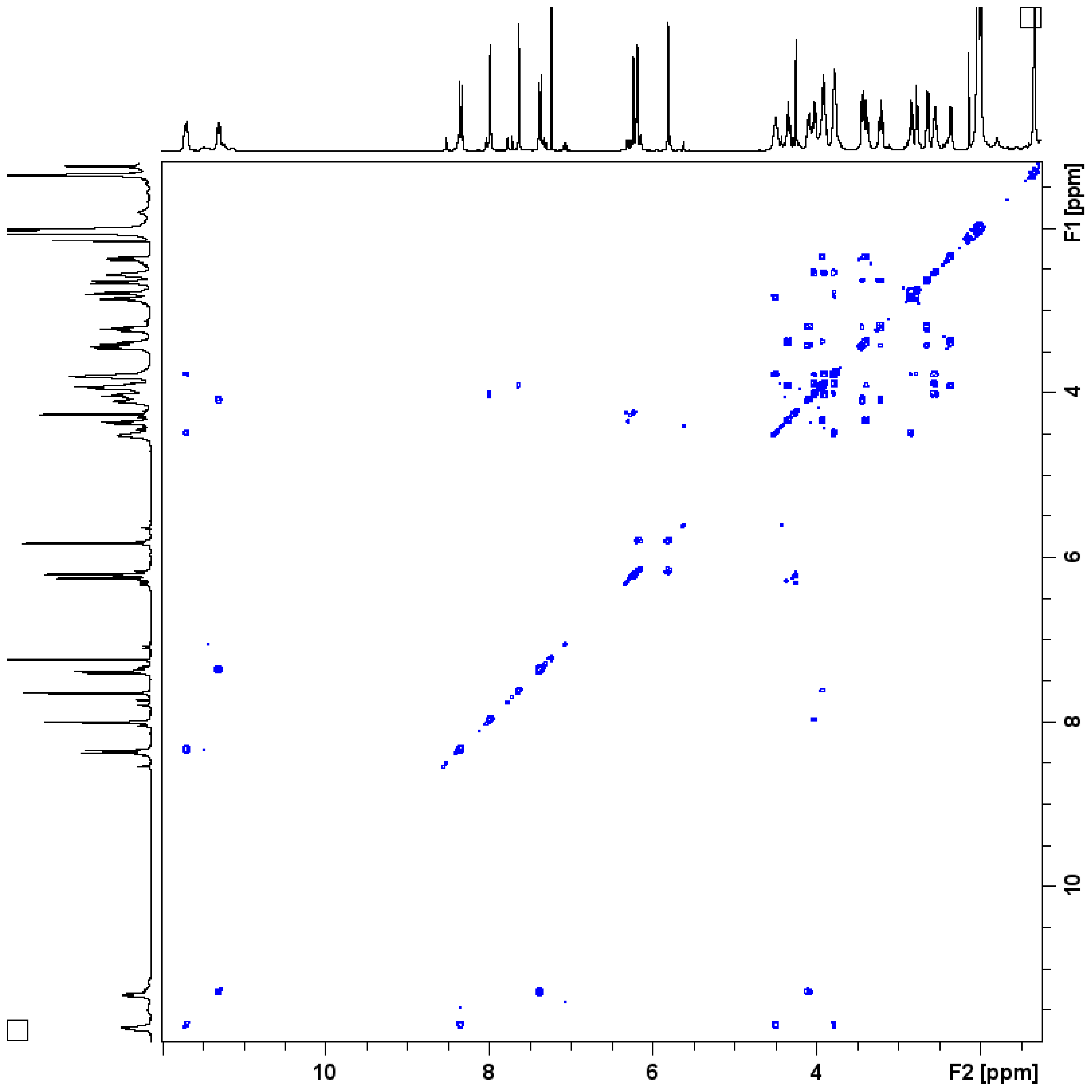

Figure S26. The ^{1}H-^{1}H COSY spectrum of **3-Zn$_{12}$** ($CDCl_3$, 300 K, 600 MHz).

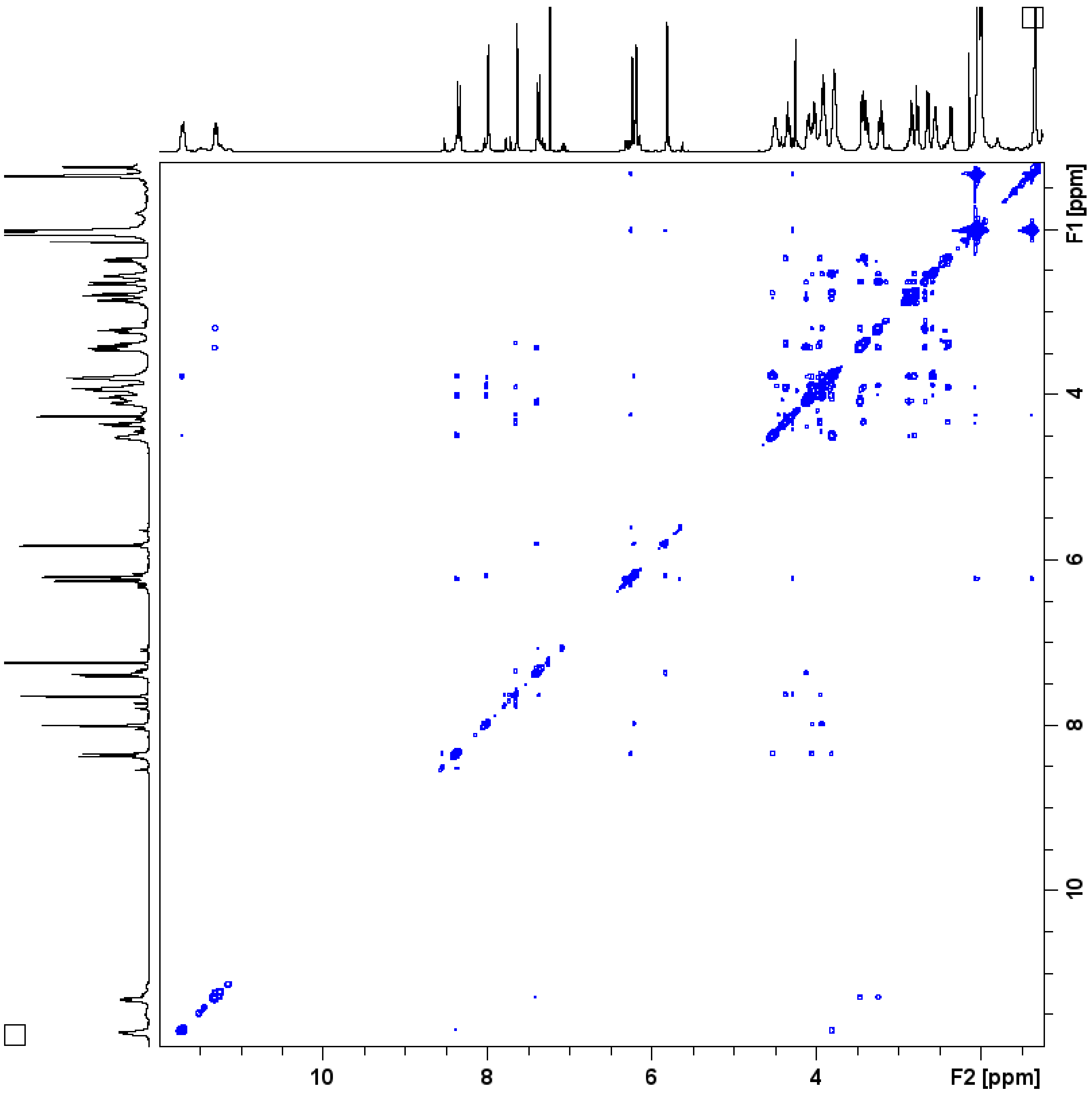

Figure S27. The ^{1}H-^{1}H NOESY spectrum of **3-Zn$_{12}$** ($CDCl_3$, 300 K, 600 MHz).

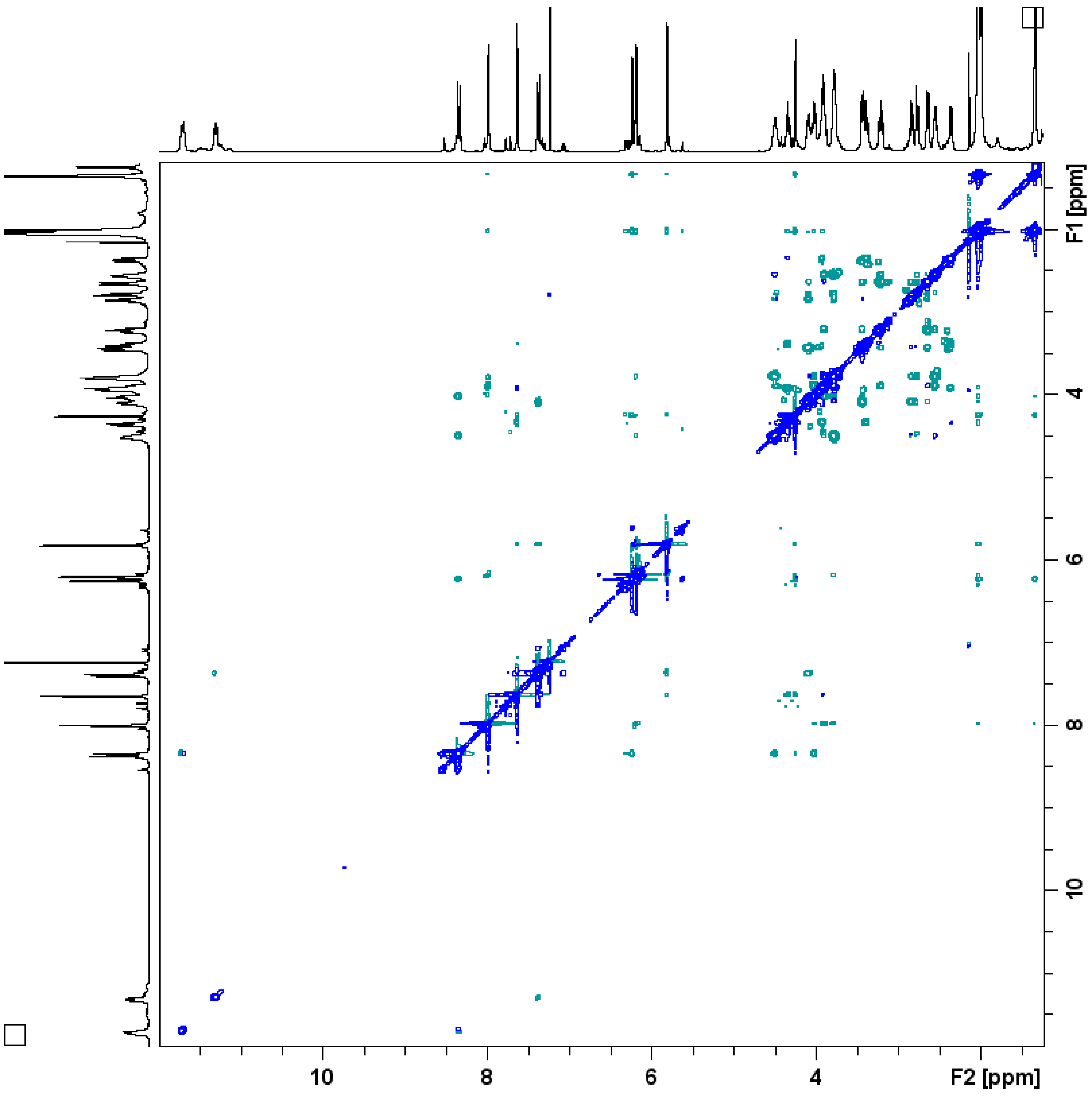

Figure S28. The ^{1}H-^{1}H ROESY spectrum of **3-Zn$_{12}$** ($CDCl_3$, 300 K, 600 MHz).

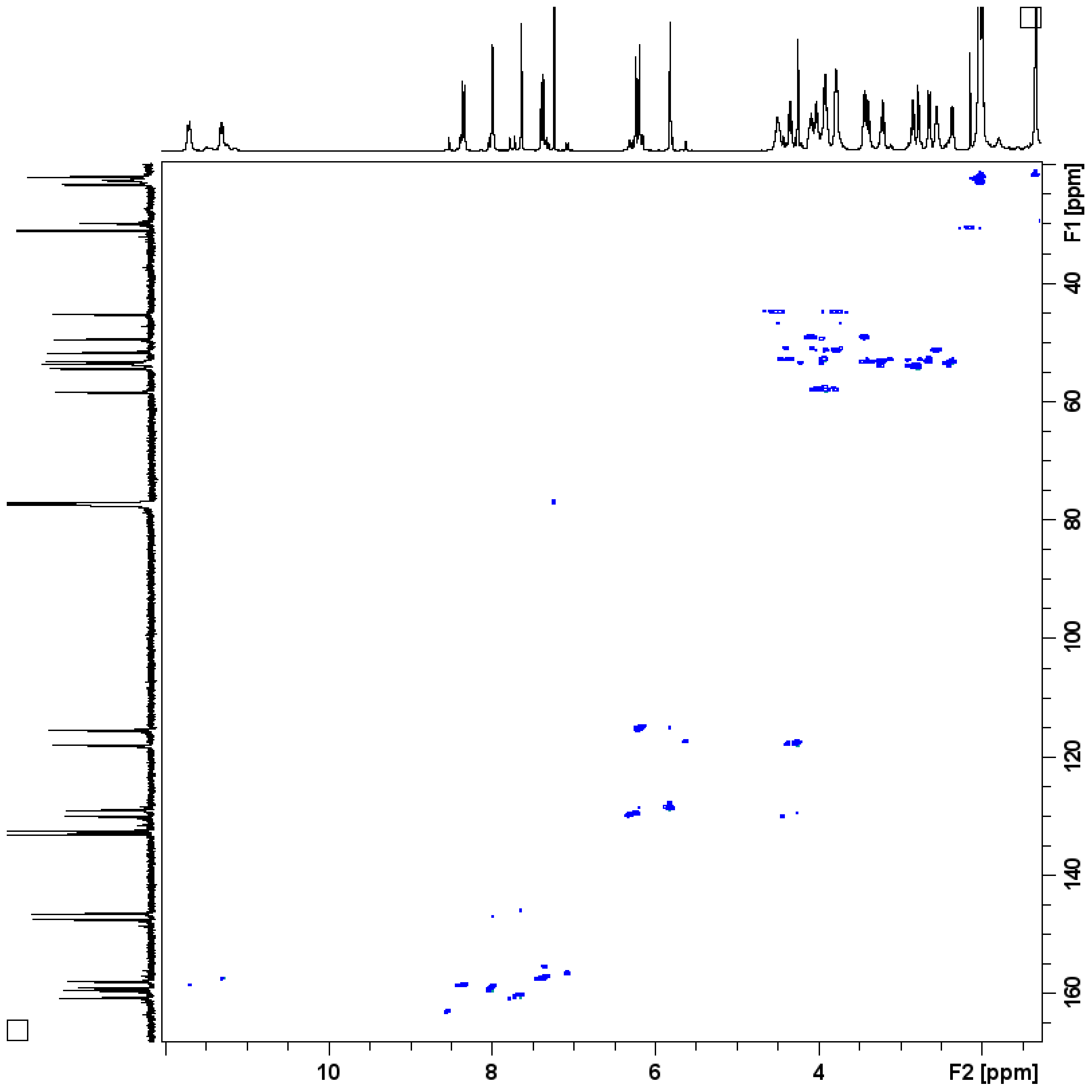

Figure S29. The ^{1}H-^{13}C HSQC spectrum of **3-Zn$_{12}$** ($CDCl_3$, 300 K, 600 MHz).

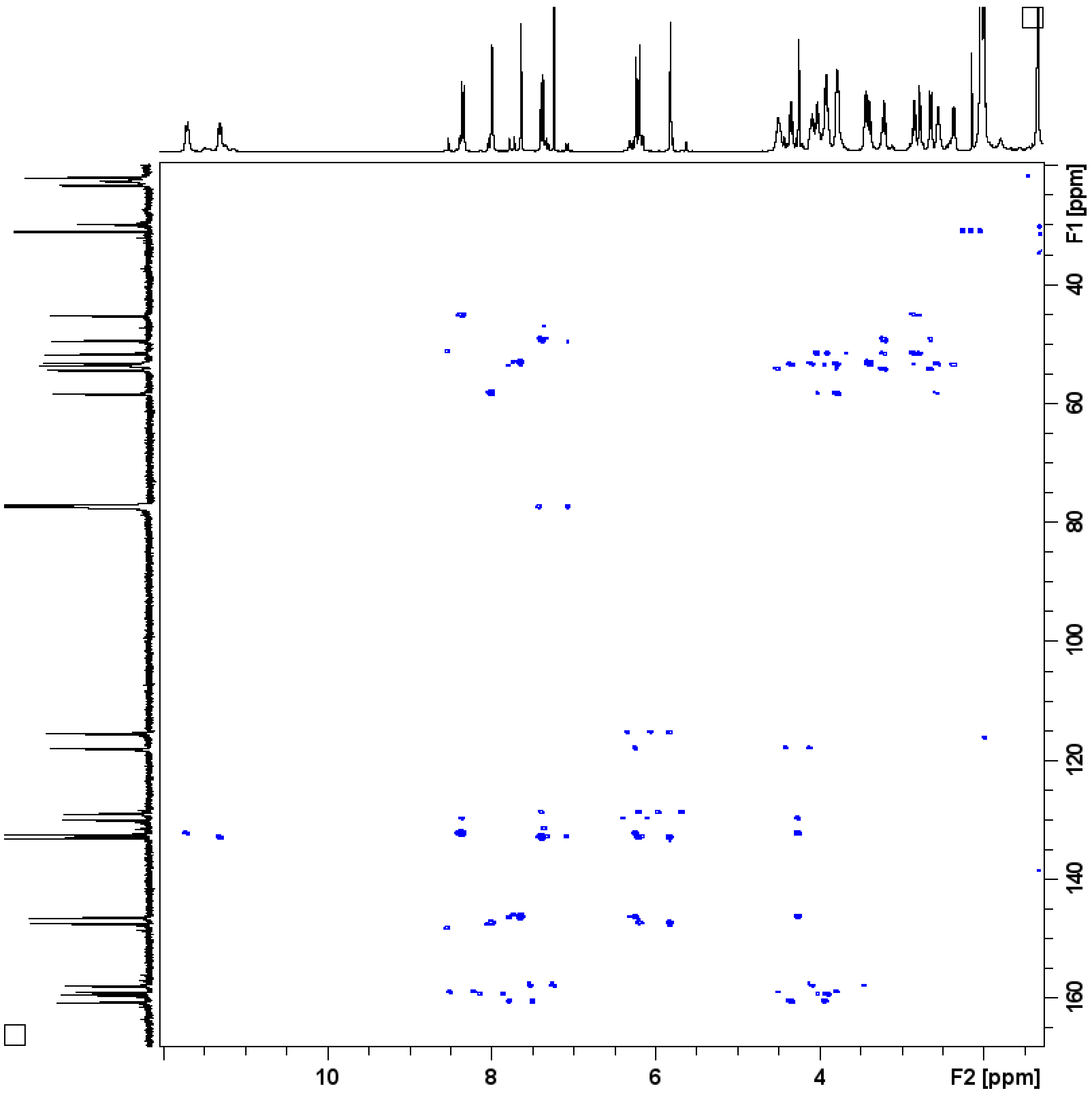

Figure S30. The ^{1}H-^{13}C HMBC spectrum of **3-Zn$_{12}$** ($CDCl_3$, 300 K, 600 MHz).

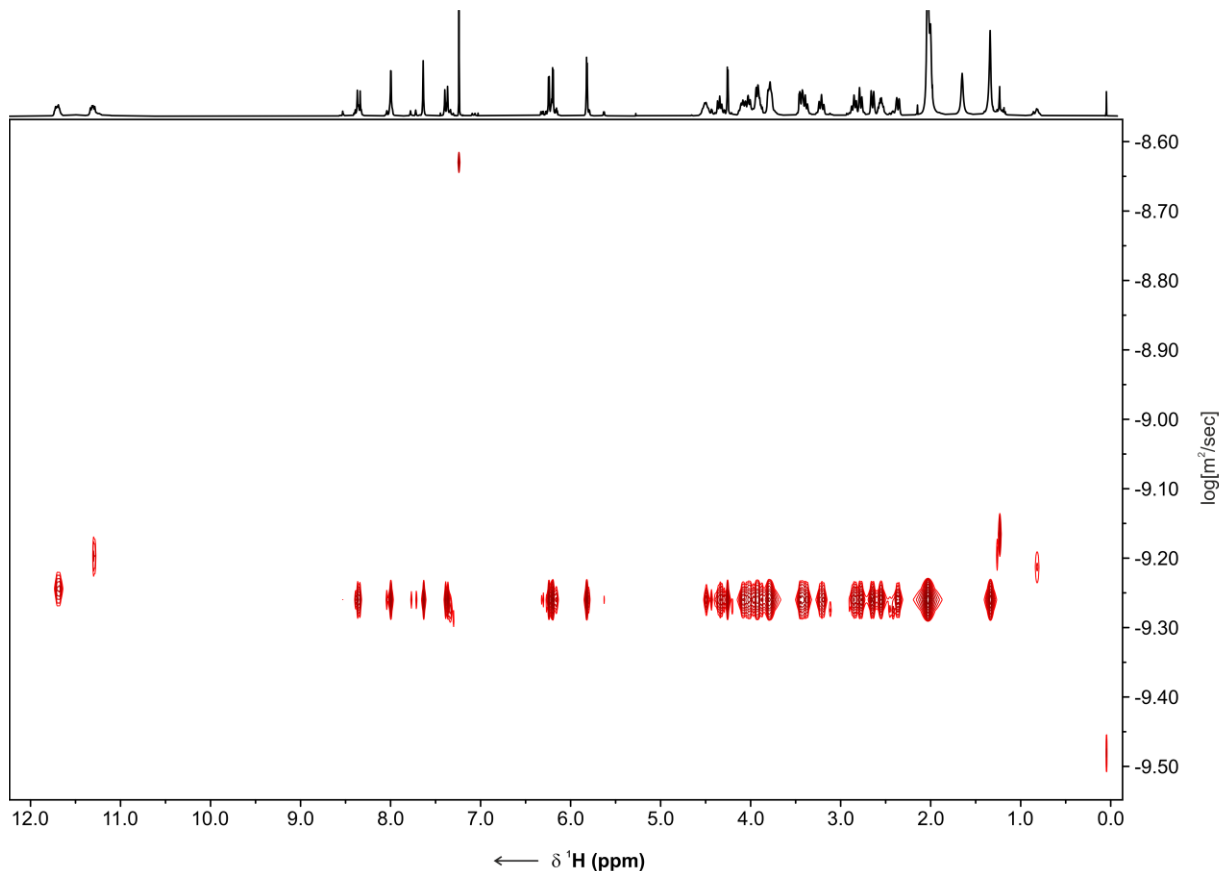

Figure S31. The DOSY NMR spectrum of **3-Zn$_{12}$** ($CDCl_3$, 300 K, 600 MHz).

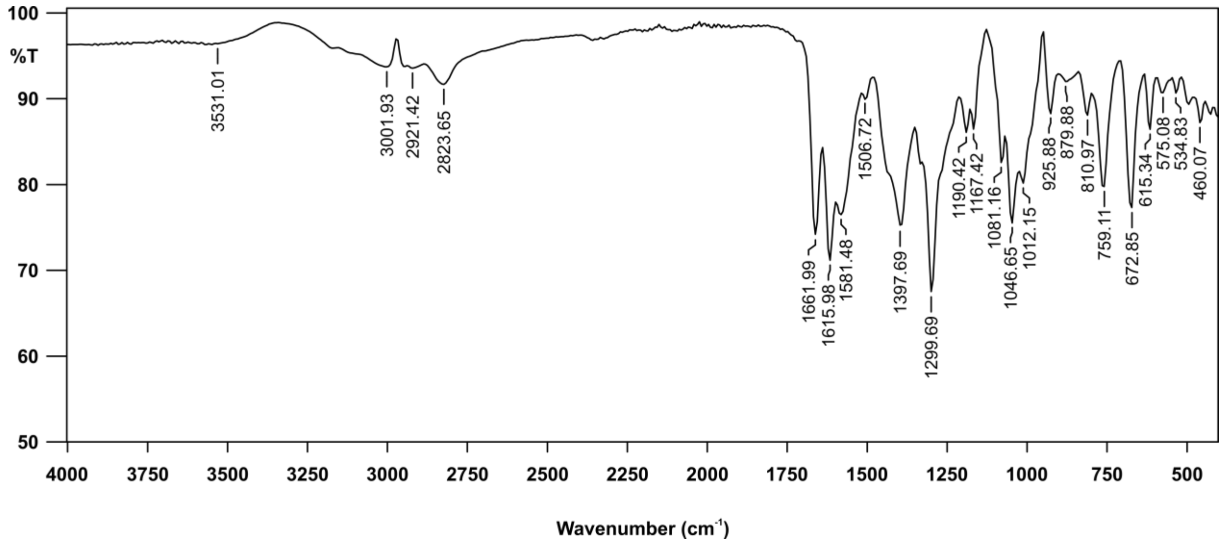

Figure S32. The FT-IR spectrum of **3-Zn$_{12}$**.

Analytical data for 4-Zn4

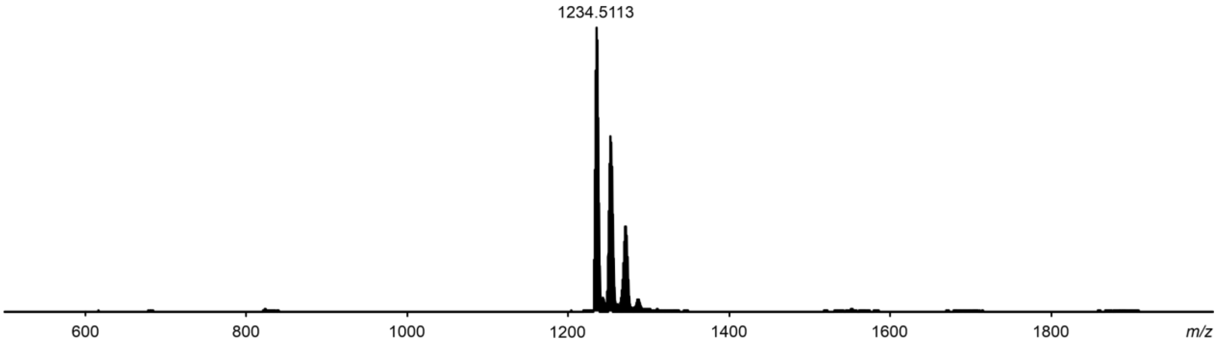

Figure S33. The 500-2000 *m/z* range of the high-resolution mass spectrum of a **4-Zn4** (ESI+, TOF).

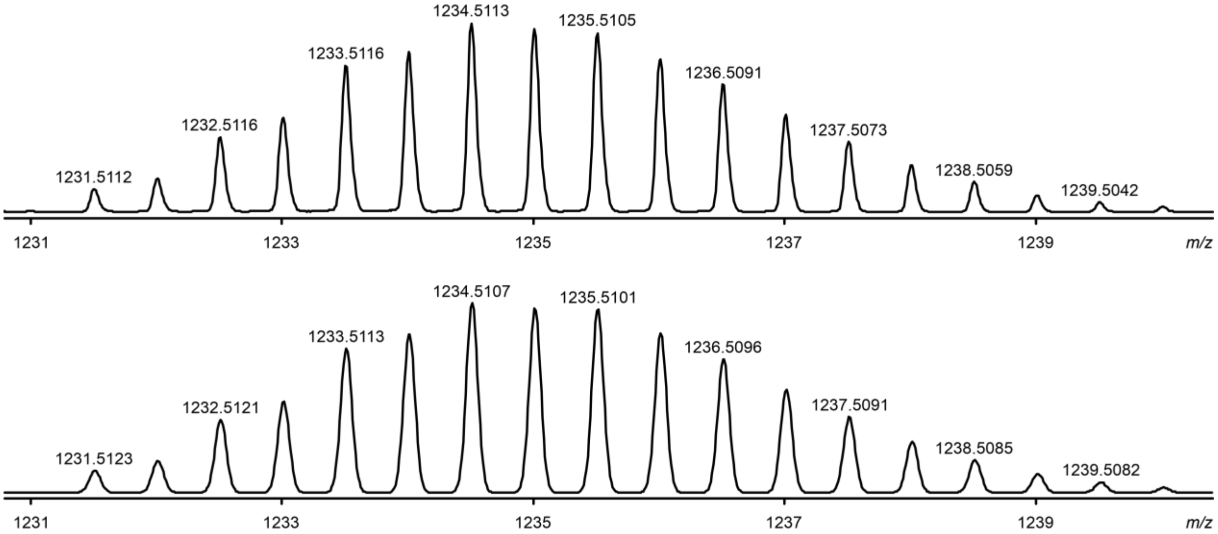

Figure S34. The high-resolution mass spectrum of **4-Zn4** (ESI+, TOF, $[C_{120}H_{150}N_{44}Zn_4]^{2+}$). Top: experimental spectrum, bottom: simulated pattern.

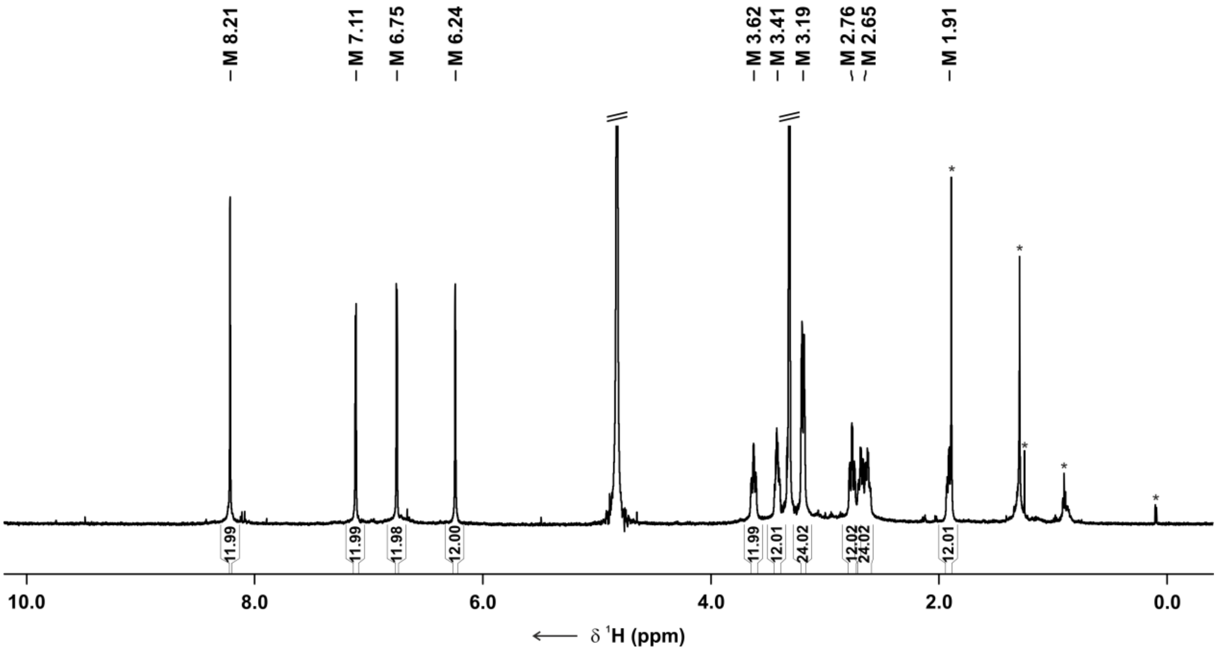

Figure S35. The ^{1}H NMR spectrum of **4-Zn₄** (CD₃OD, 300 K, 600 MHz).

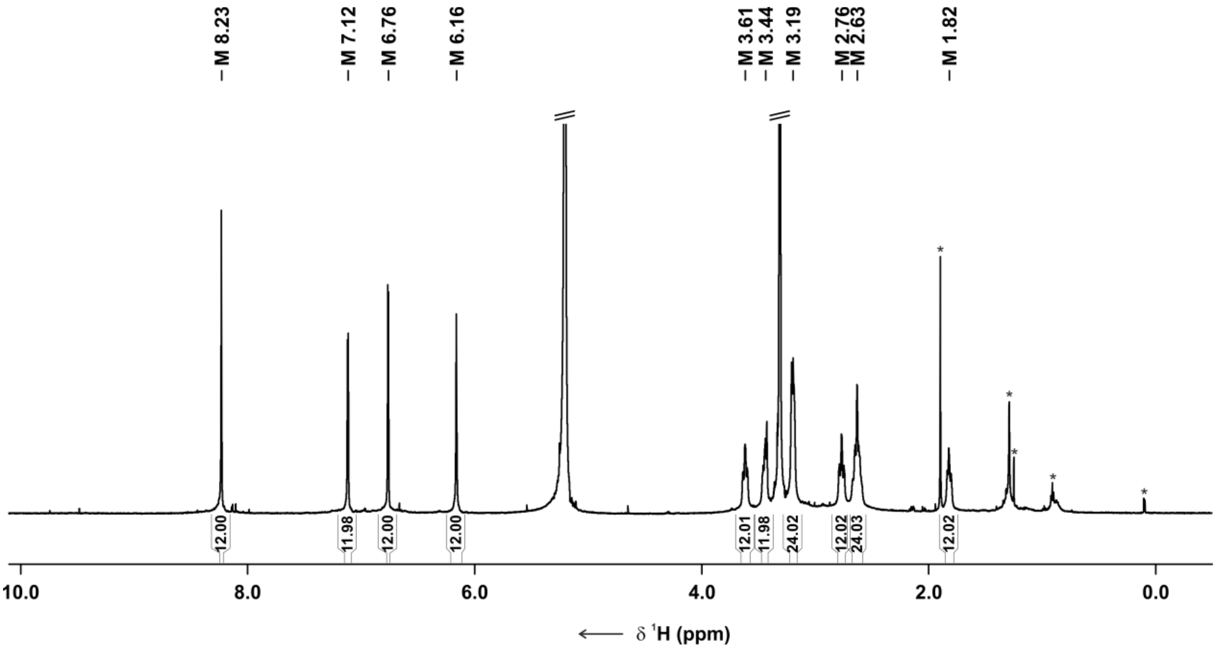

Figure S36. The ^{1}H NMR spectrum of **4-Zn₄** (CD₃OD, 260 K, 600 MHz).

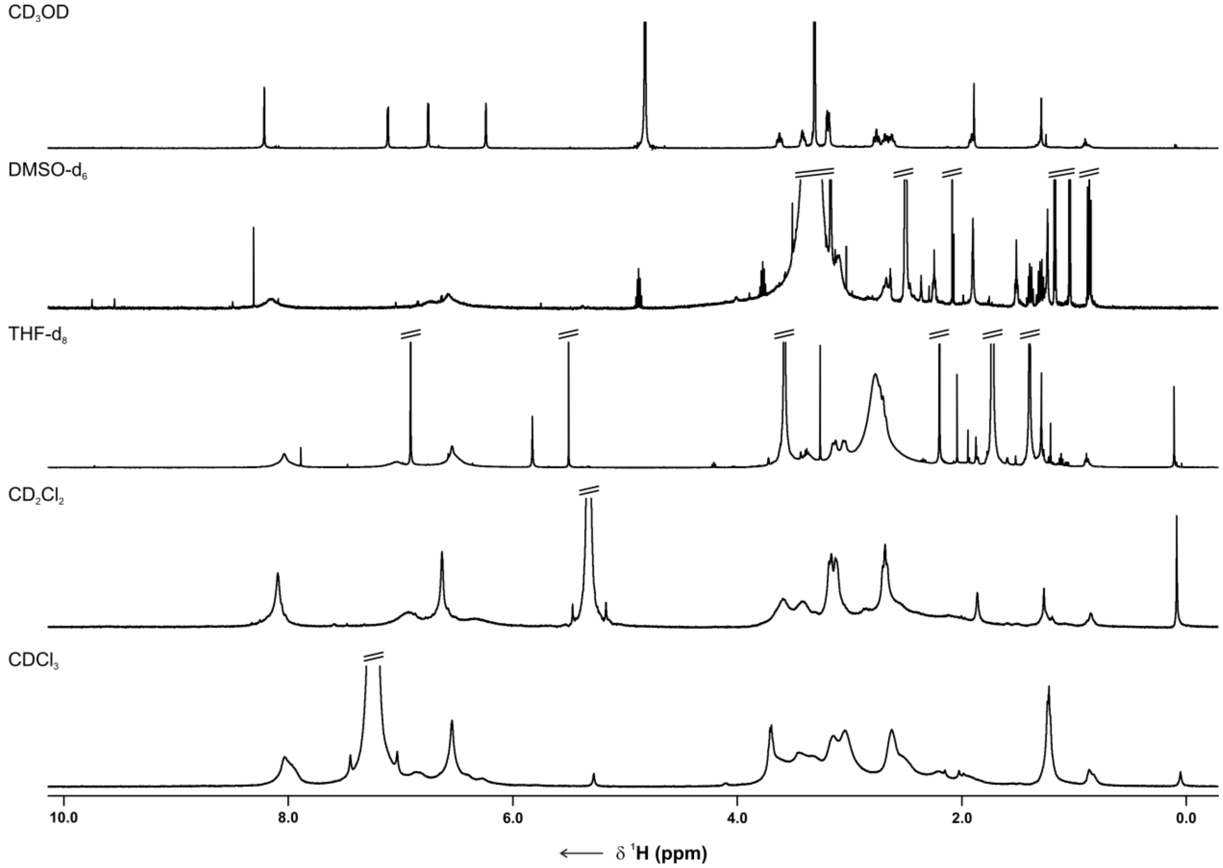

Figure S37. The ^{1}H NMR spectra of **4-Zn₄** in various deuterated solvents (300 K, 600 MHz).

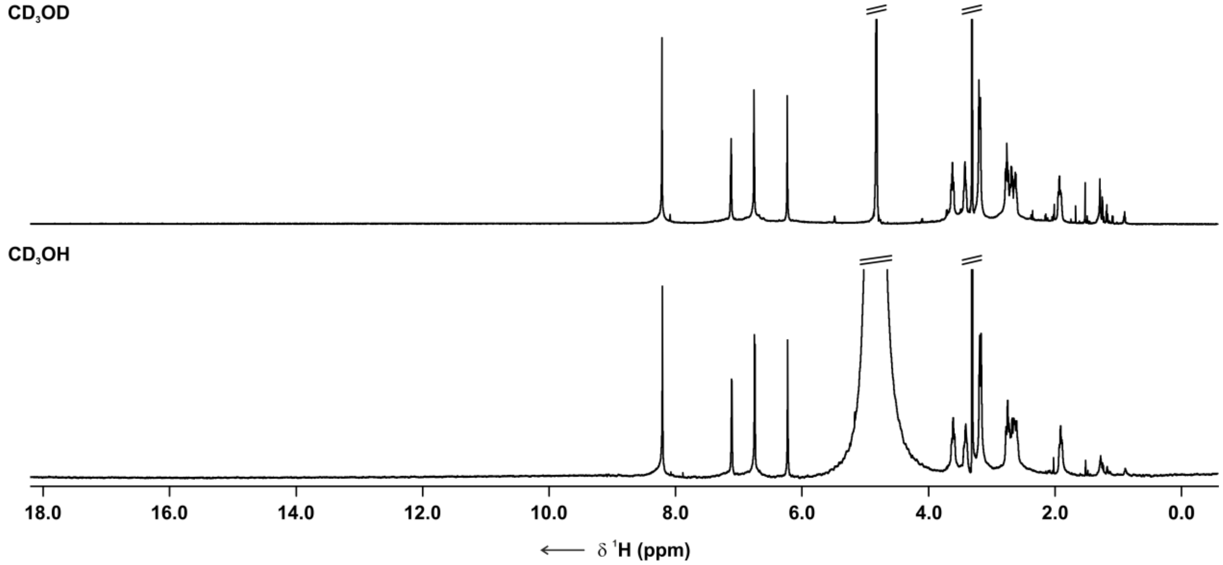

Figure S38. The ^{1}H NMR spectra of **4-Zn$_4$** in CD_3OD and CD_3OH (300 K, 600 MHz).

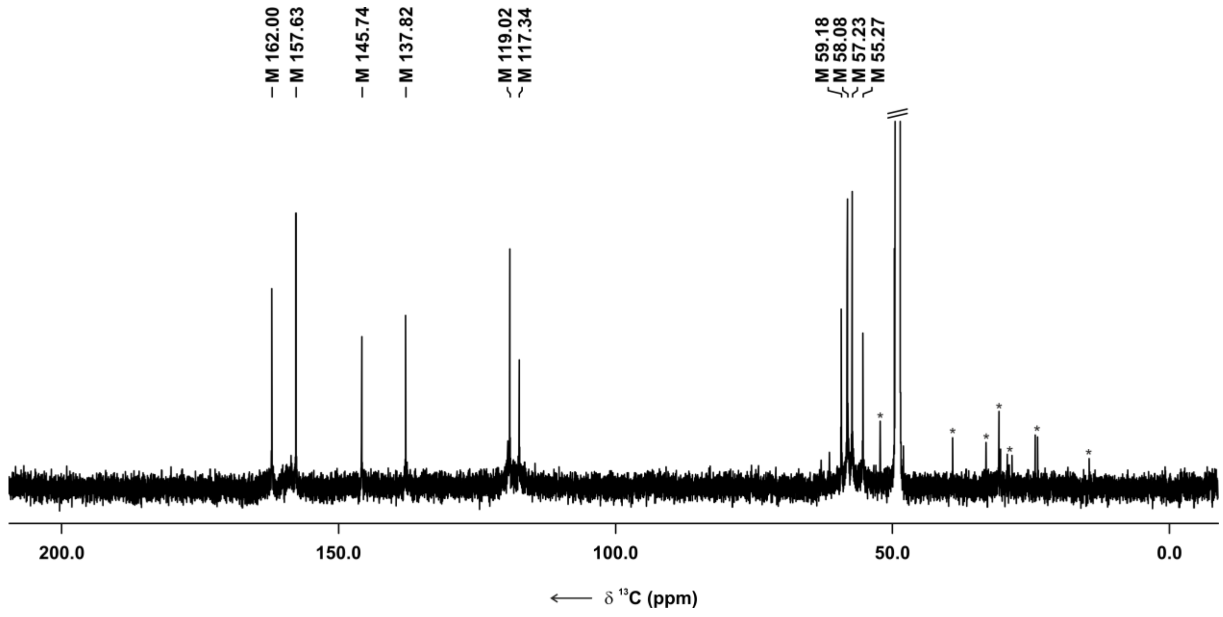

Figure S39. The ^{13}C NMR spectrum of **4-Zn$_4$** (CD_3OD, 300 K, 151 MHz).

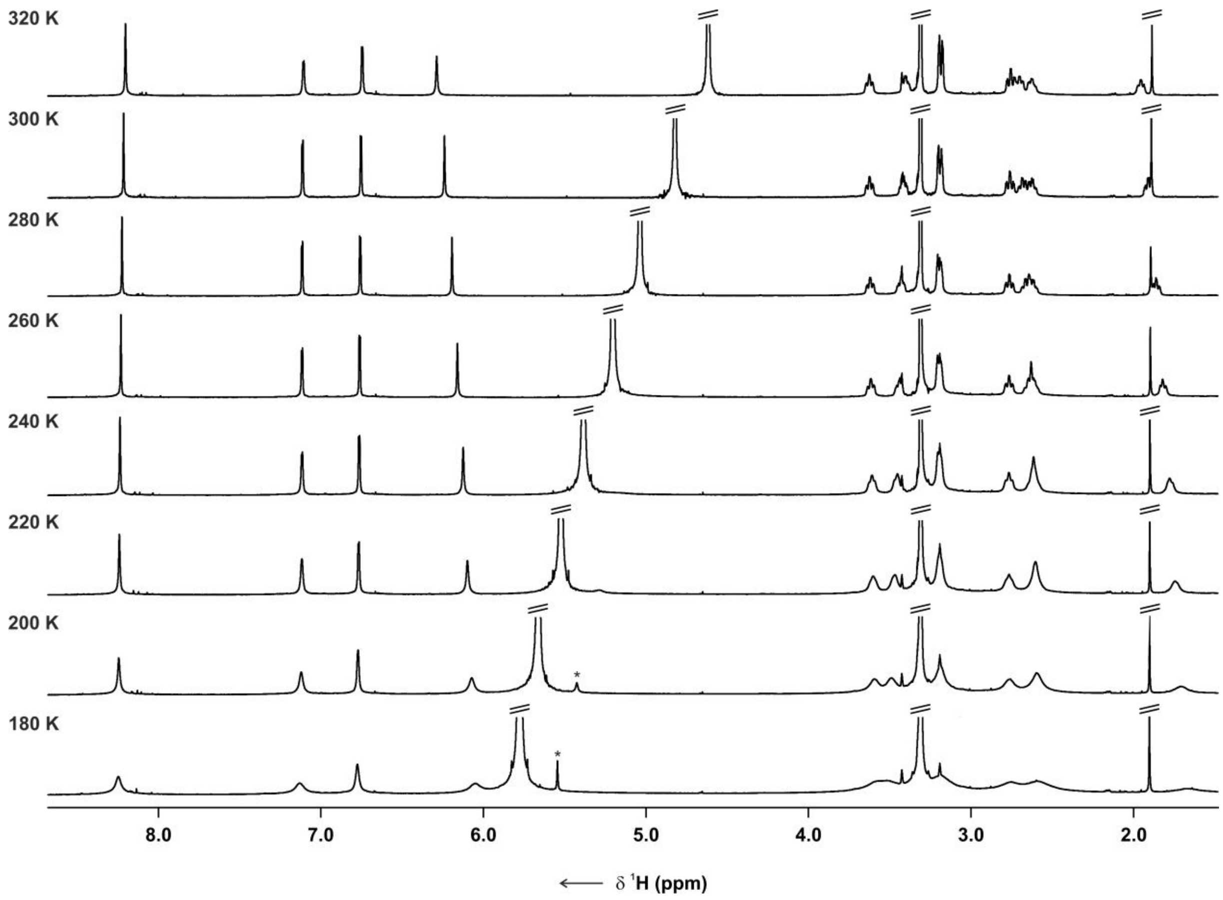

Figure S40. The ^{1}H NMR spectra of **4-Zn₄** recorded in the 320 K – 180 K temperature range (CD_3OD, 600 MHz).

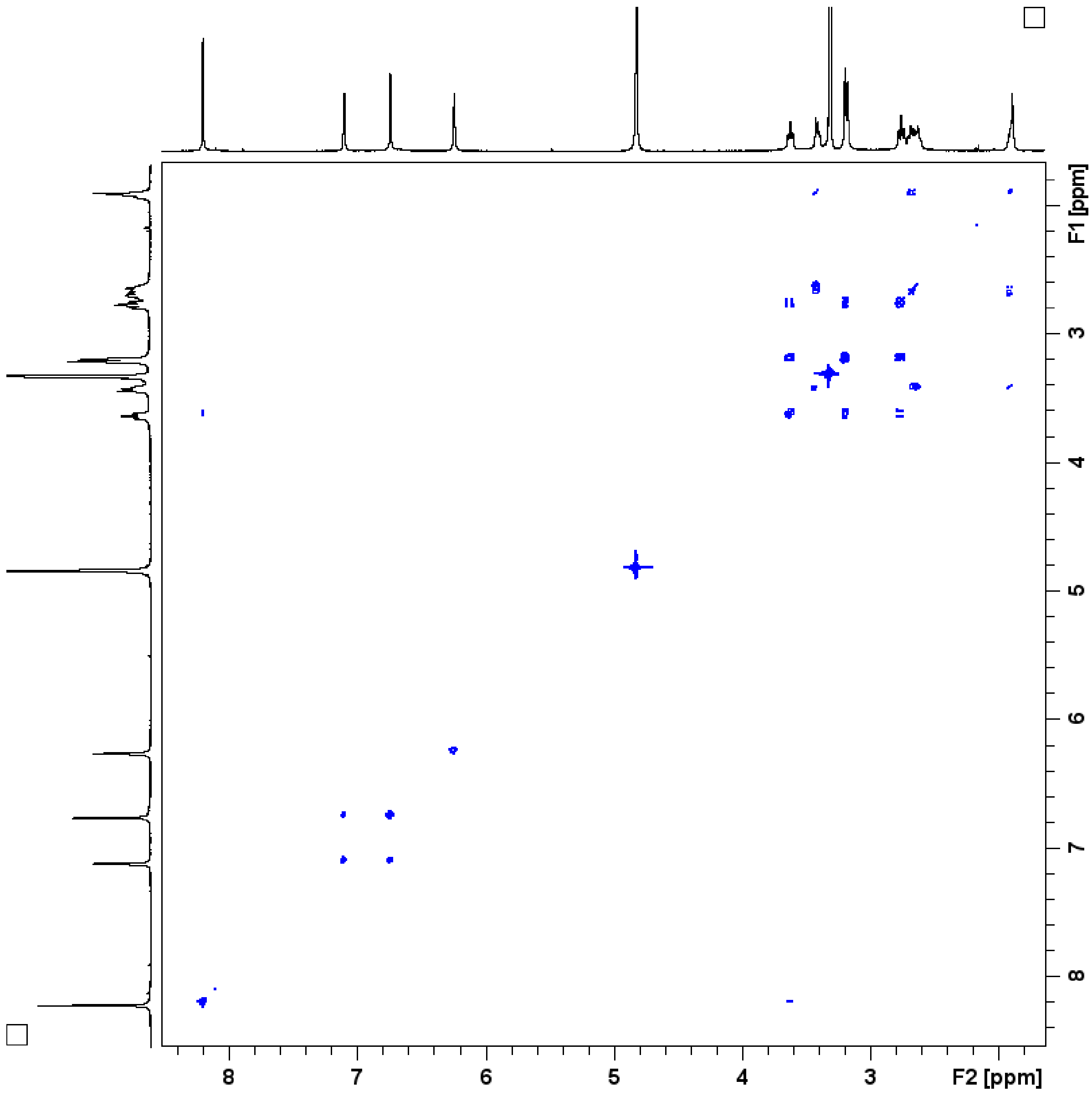

Figure S41. The ^1H-^1H COSY spectrum of **4-Zn$_4$** (CD_3OD, 300 K, 600 MHz).

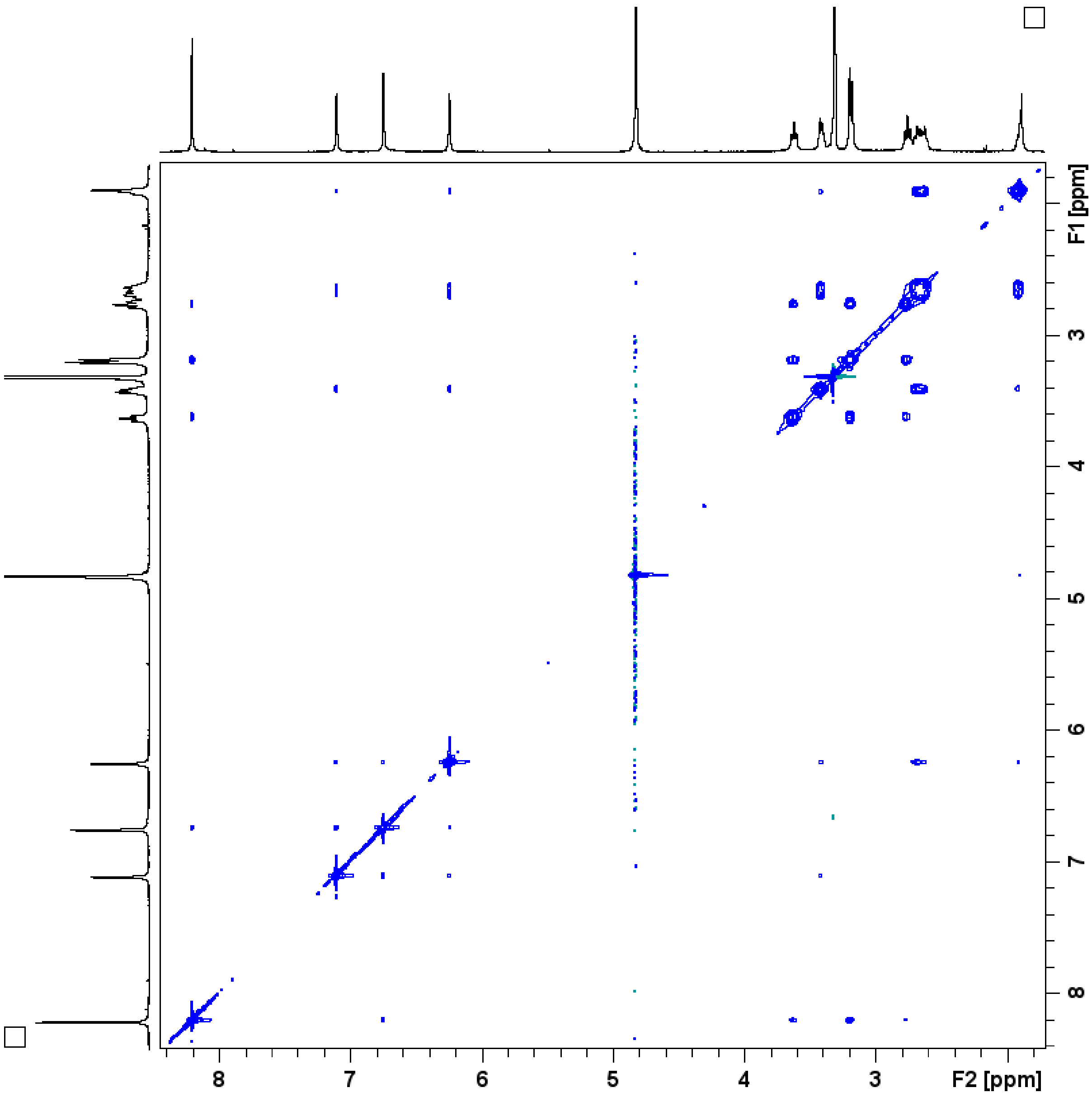

Figure S42. The ^{1}H-^{1}H NOESY spectrum of **4-Zn4** (CD_3OD, 300 K, 600 MHz).

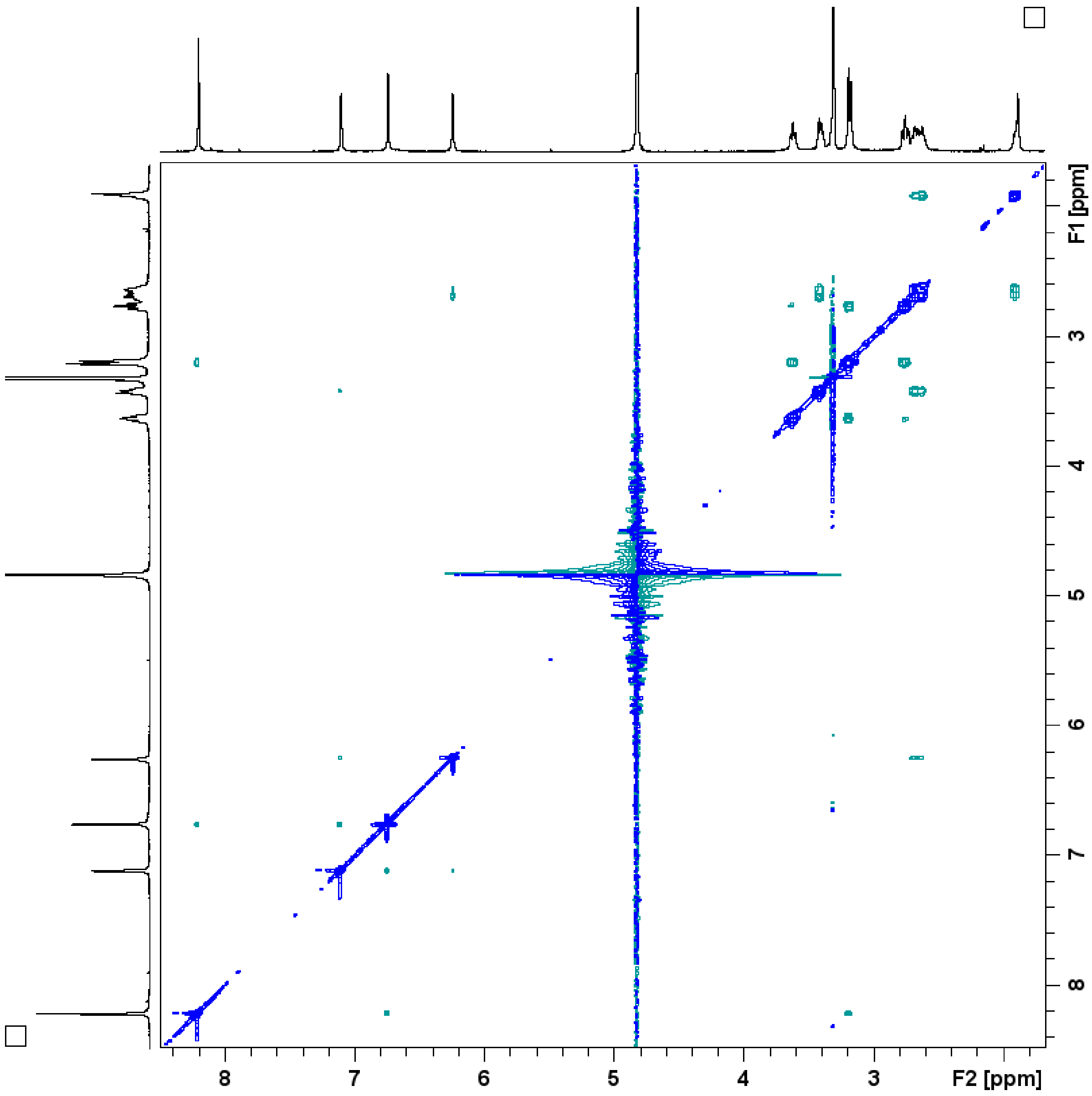

Figure S43. The ^1H-^1H ROESY spectrum of **4-Zn₄** (CD₃OD, 300 K, 600 MHz).

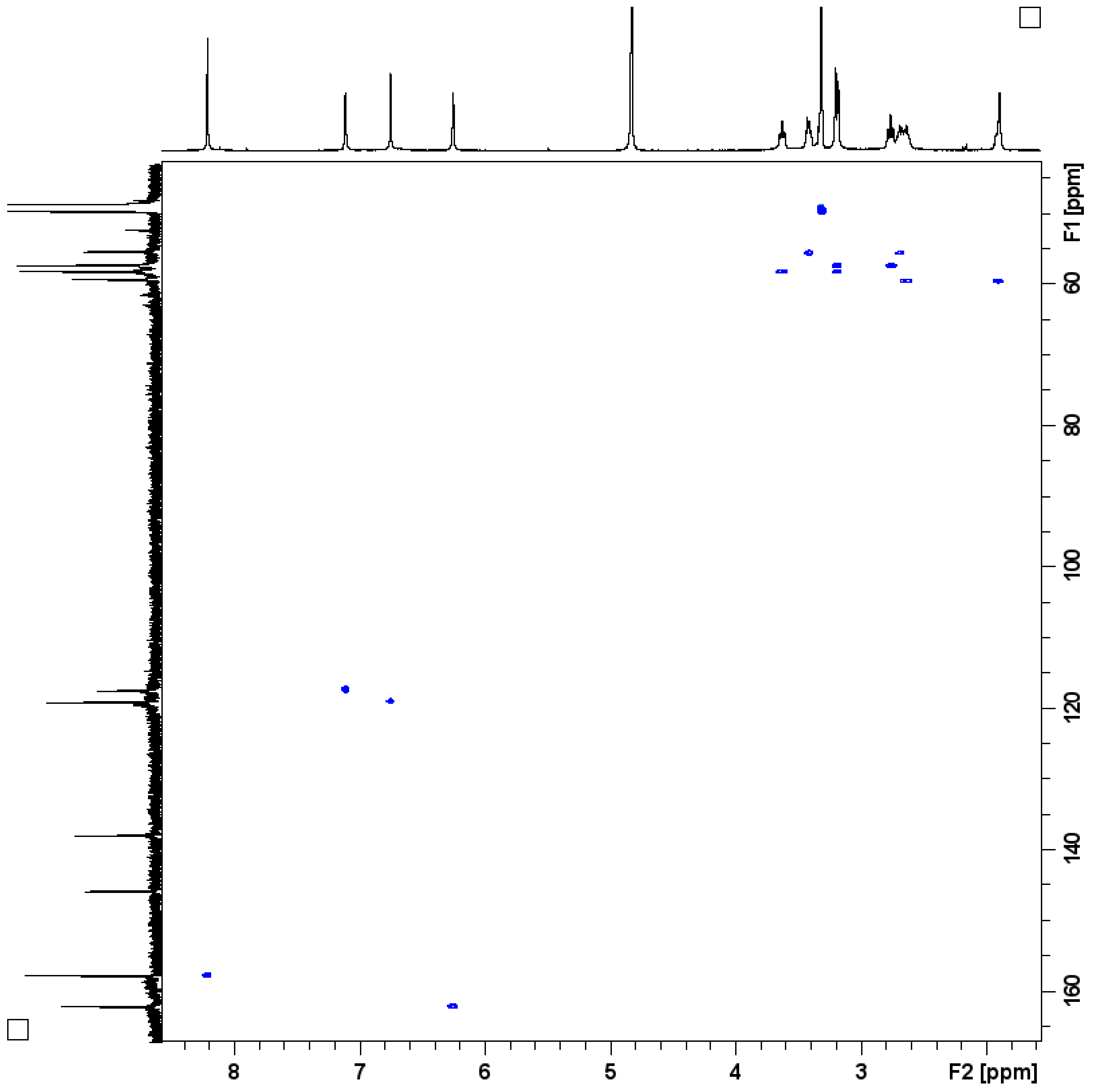

Figure S44. The ^1H-^{13}C HSQC spectrum of **4-Zn$_4$** (CD_3OD, 300 K, 600 MHz).

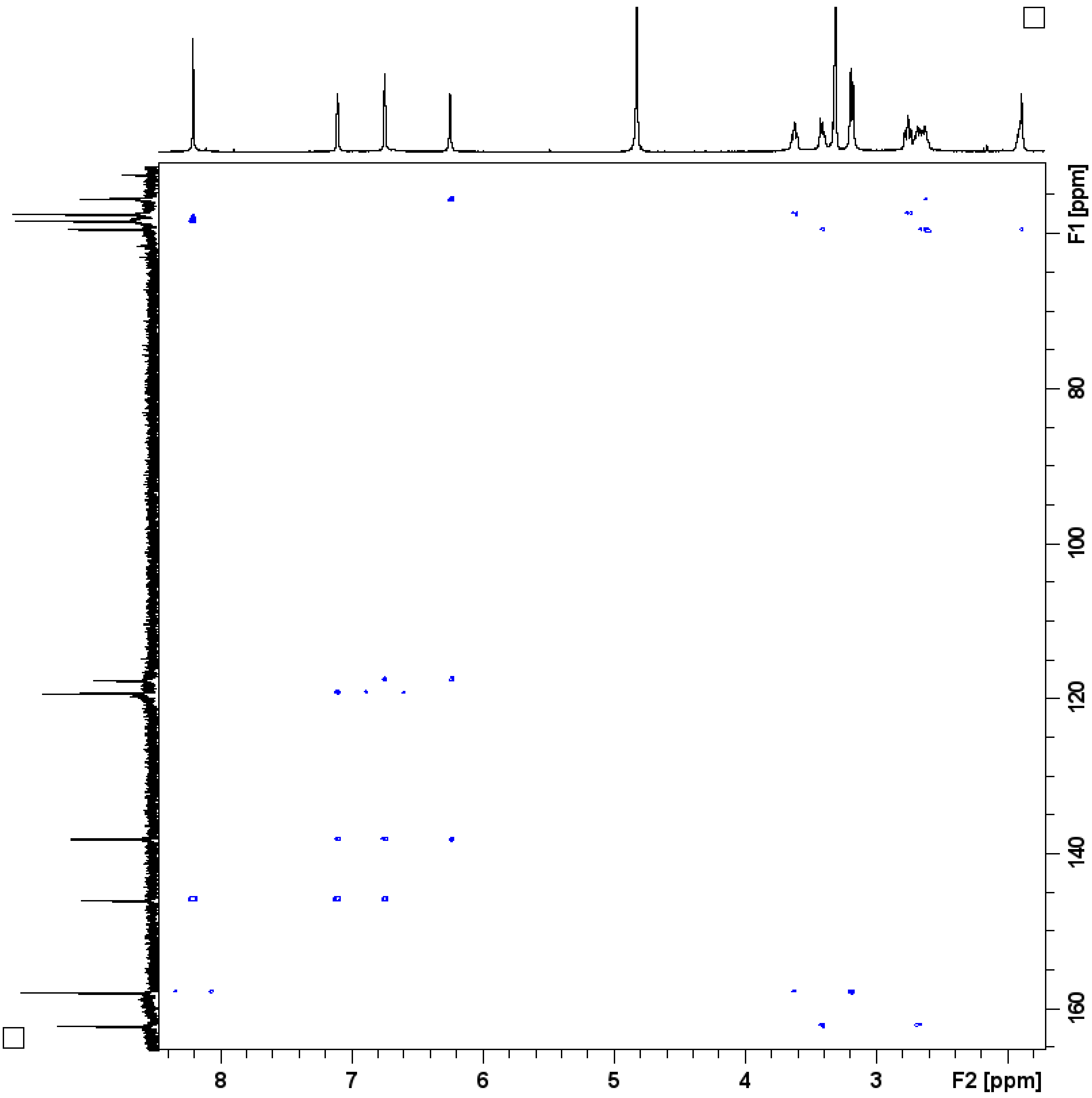

Figure S45. The ^{1}H-^{13}C HMBC spectrum of **4-Zn$_4$** (CD_3OD, 300 K, 600 MHz).

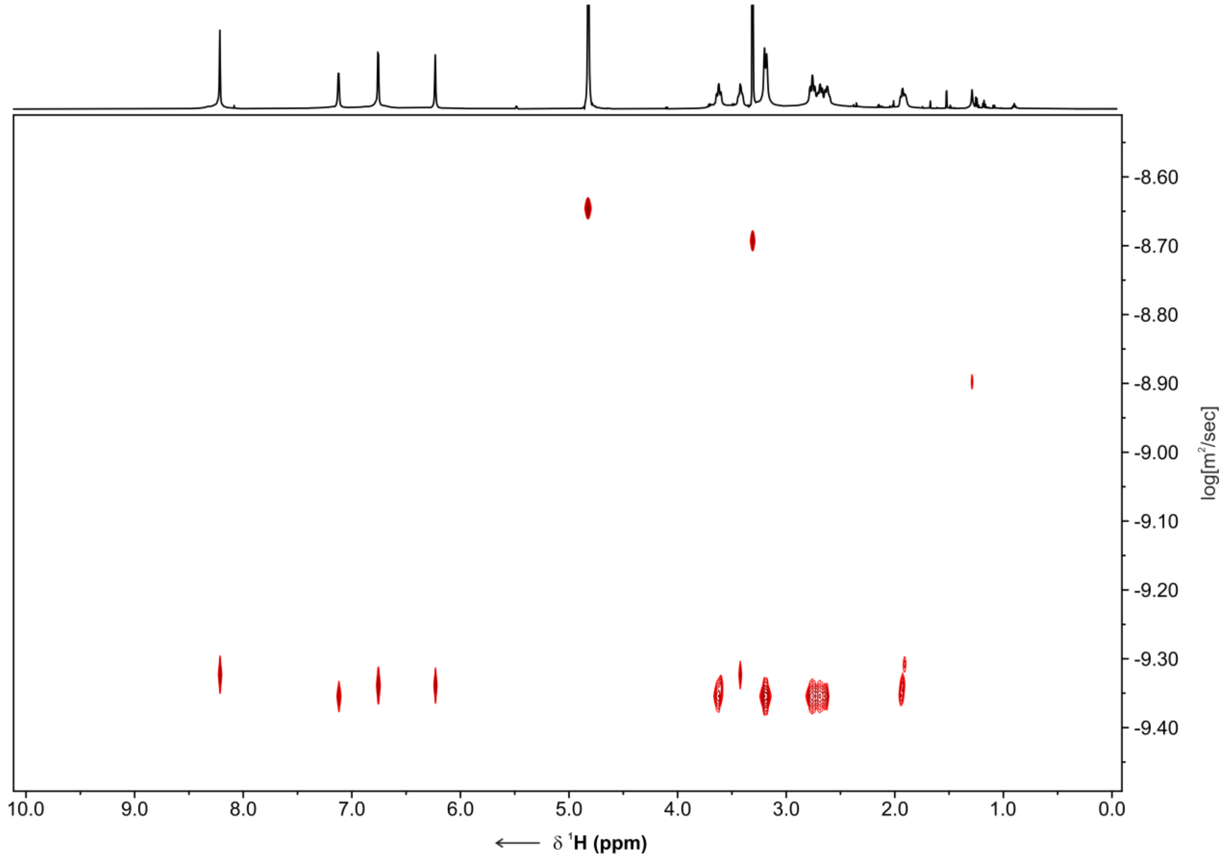

Figure S46. The DOSY NMR spectrum of **4-Zn₄** (CD3OD, 300 K, 600 MHz).

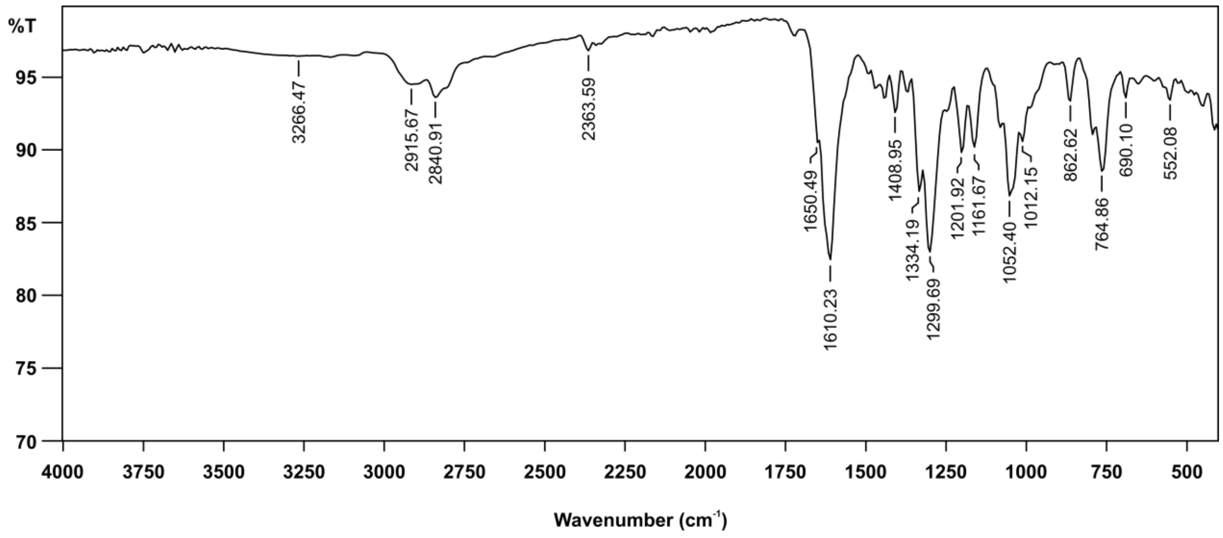

Figure S47. The FT-IR spectrum of **4-Zn₄**.

Transformations of cages

Transformation of [1-Zn_2]OAc to $H_2O\subset$2-Zn_2

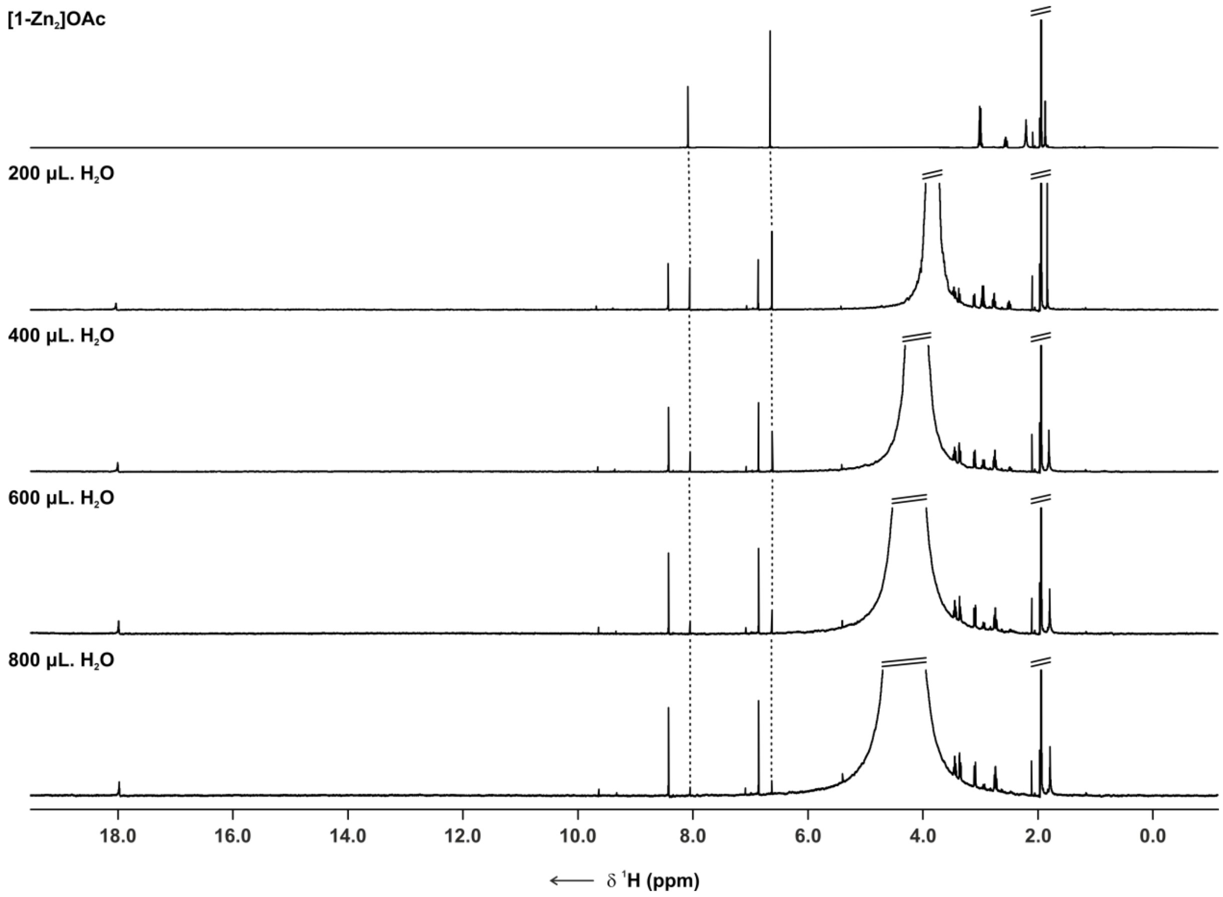

Figure S48. The ^{1}H NMR spectra recorded upon the addition of H_2O to **[1-Zn_2]OAc** (CD_3CN, 300 K, 600 MHz).

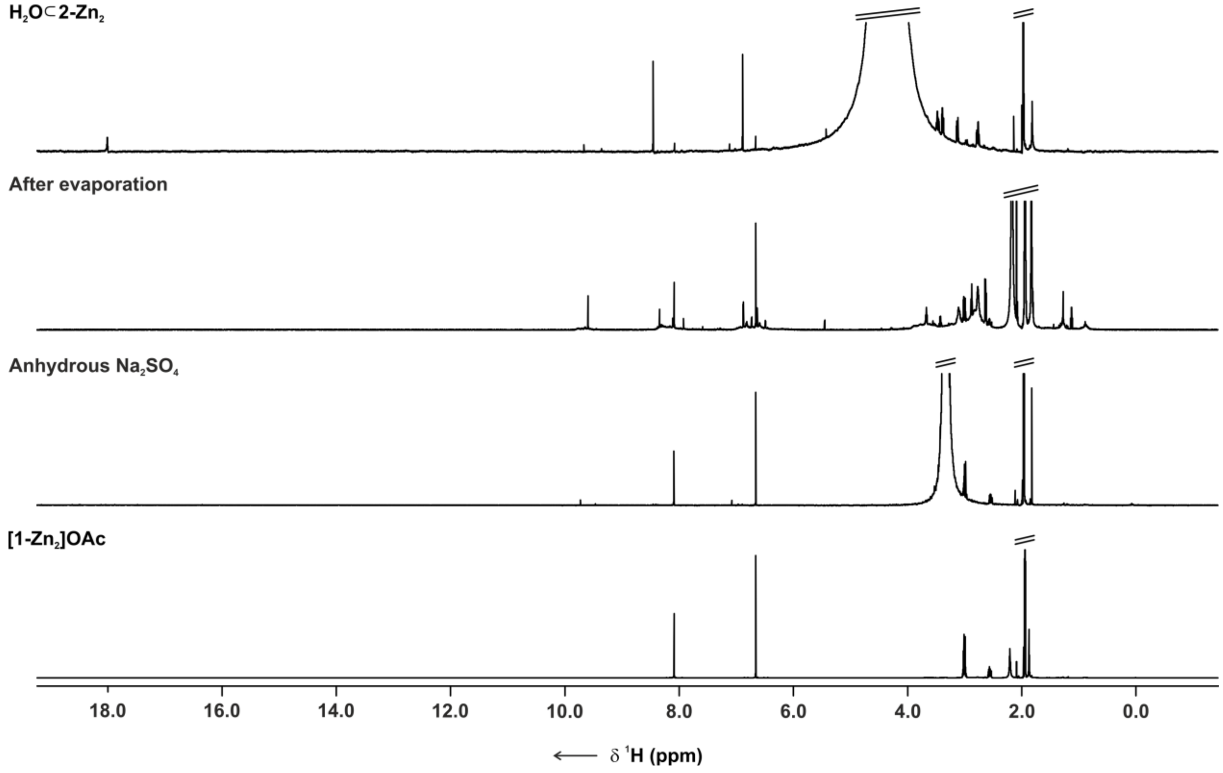

Figure S49. The ^{1}H NMR spectra of **[1-Zn$_2$]OAc** and **H$_2$O⊂2-Zn$_2$**, and recorded for the sample after evaporation of **H$_2$O⊂2-Zn$_2$** under vacuum, and after stirring **H$_2$O⊂2-Zn$_2$** with anhydrous Na_2SO_4 (CD_3CN, 300 K, 600 MHz).

Contraction of 3-Zn_{12} to [1-Zn_2]OAc

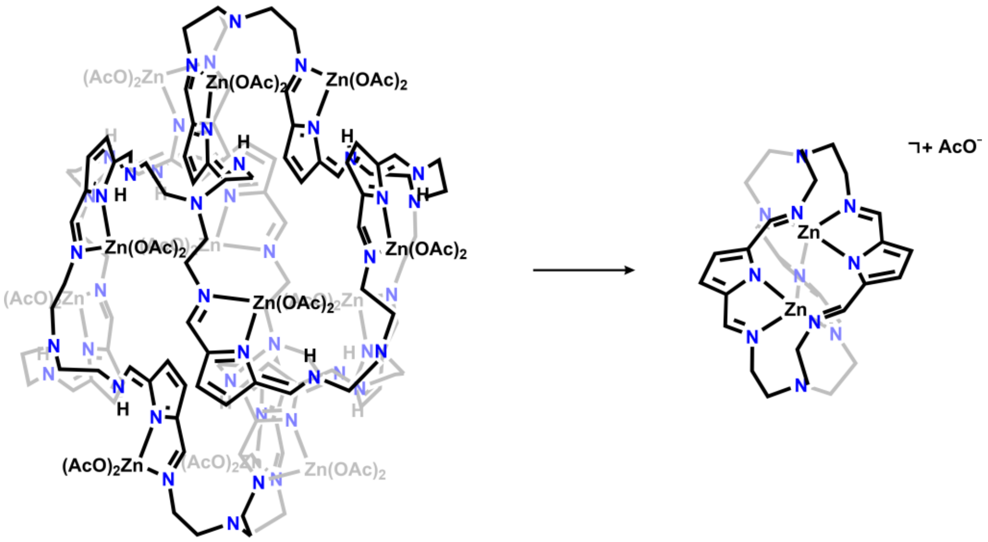

3-Zn_{12} (44.2 mg, 0.01 mmol) was placed in a 25 mL round-bottom flask equipped with a magnetic stirrer and dissolved in 10 mL of *n*-butanol. Flask was equipped with a reflux condenser, and the mixture was stirred under reflux for 17 hours. After completion of the reaction, the solution was concentrated to ca. 2 mL under reduced pressure. Et_2O was added in excess, inducing precipitation of **[1-Zn_2]OAc**. The suspension was transferred to a centrifuge tube and centrifuged for 5 minutes (6000 rpm). The supernatant was separated from the solid, and the washing procedure with Et_2O was repeated twice. The precipitate was then treated with $CHCl_3$ and centrifuged again for 5 minutes (6000 rpm) to purify **[1-Zn_2]OAc** further. Then the $CHCl_3$ solution was separated from the solid, and the procedure was repeated twice. Finally, it was concentrated using a rotary evaporator, yielding a ruby-red solid. The product was dried under vacuum for 1 hour. Yield: 21.1 mg (71%).

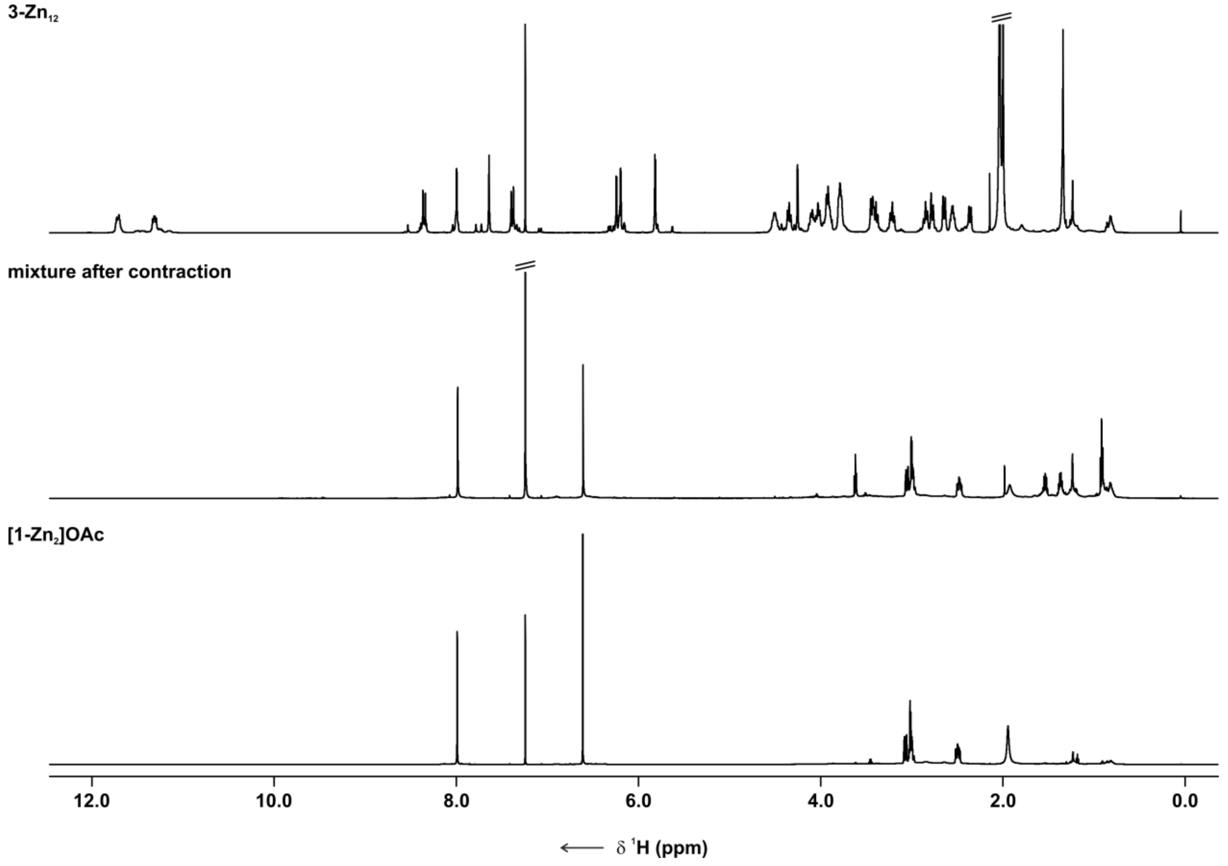

Figure S50. The ^{1}H NMR spectra of **3-Zn$_{12}$**, **[1-Zn$_2$]OAc**, and the mixture after contraction of **3-Zn$_{12}$** to **[1-Zn$_2$]OAc** ($CDCl_3$, 300 K, 600 MHz).

Contraction of 4-Zn$_4$ to [1-Zn$_2$]X

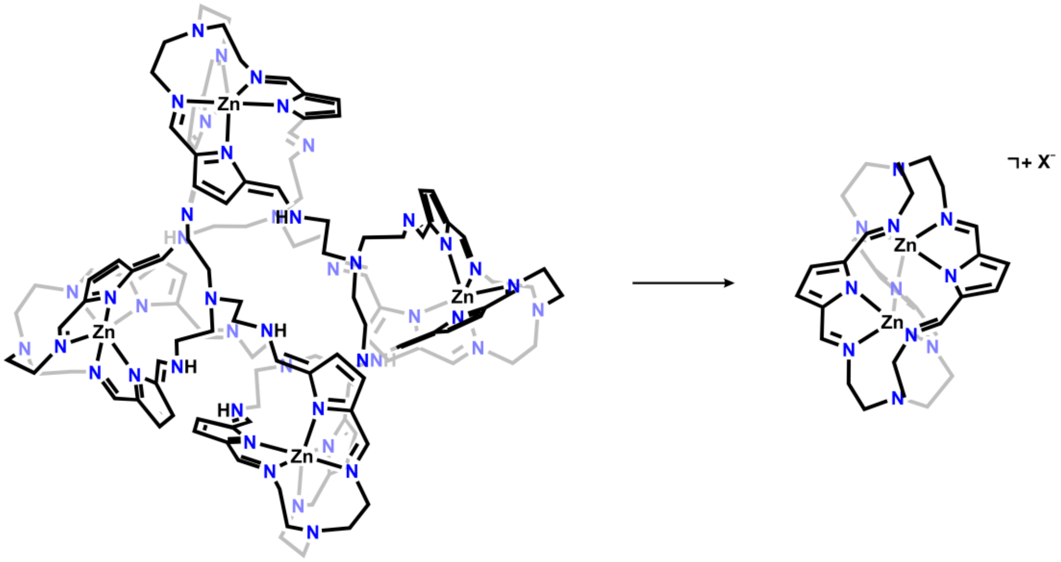

Cage 4-Zn$_4$ (24.6 mg, 0.01 mmol) was placed in a 25 mL round-bottom flask equipped with a magnetic stirrer and dissolved in 10 mL of *n*-butanol. Flask was equipped with a reflux condenser, and the mixture was stirred under reflux for 17 hours. After completion of the reaction, the solution was concentrated to ca. 2 mL under reduced pressure. Et_2O was added in excess, inducing precipitation of **[1-Zn$_2$]X**. The suspension was transferred to a centrifuge tube and centrifuged for 5 minutes (6000 rpm). The supernatant was separated from the solid, and the washing procedure with Et_2O was repeated twice. The precipitate was then treated with $CHCl_3$ and centrifuged again for 5 minutes (6000 rpm) to purify **[1-Zn$_2$]X** further. Then the $CHCl_3$ solution was separated from the solid, and the procedure was repeated twice. Finally, it was concentrated using a rotary evaporator, yielding a ruby-red solid. The product was dried under vacuum for 1 hour.

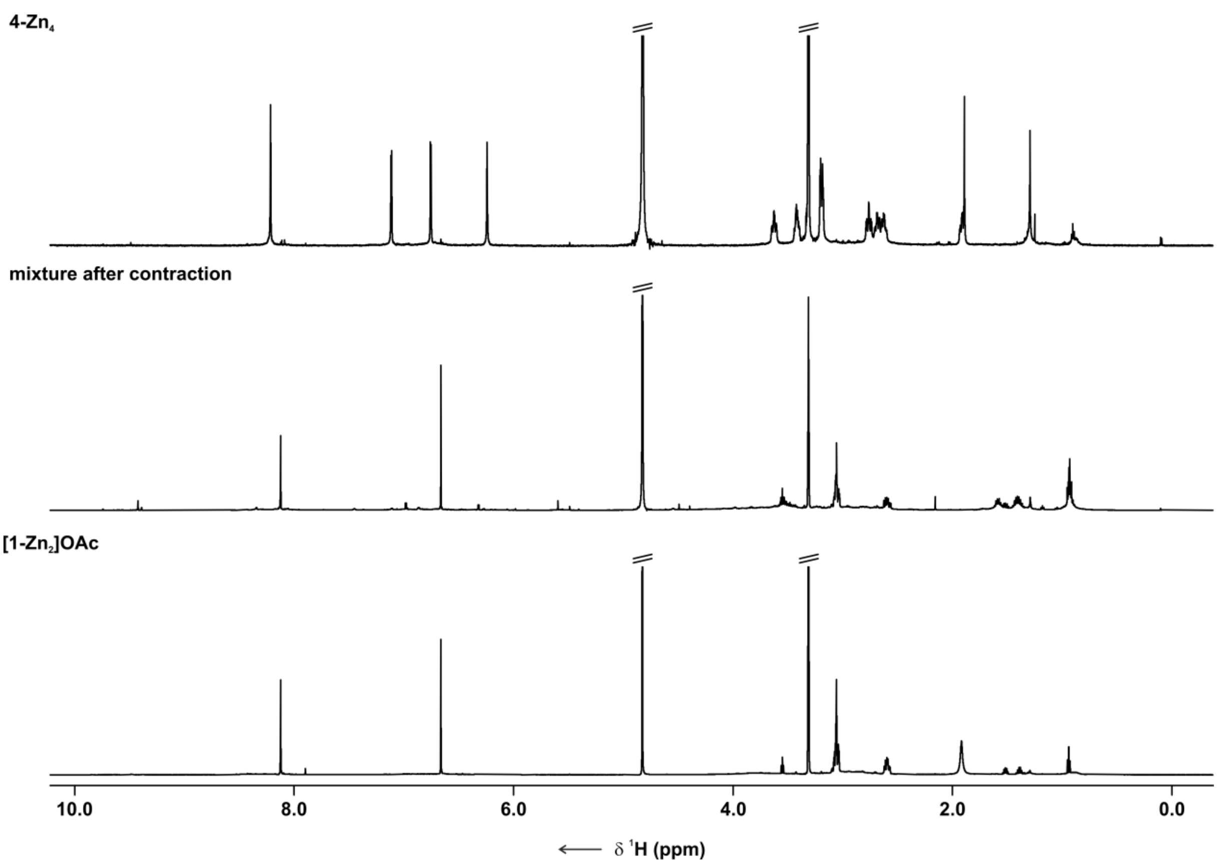

Figure S51. The ^{1}H NMR spectra of **4-Zn$_4$**, **[1-Zn$_2$]OAc** and crude mixture after contraction of **4-Zn$_4$** to **[1-Zn$_2$]X** (CD_3OD, 300 K, 600 MHz).

Transformation of 4-Zn$_4$ to 3-Zn$_{12}$

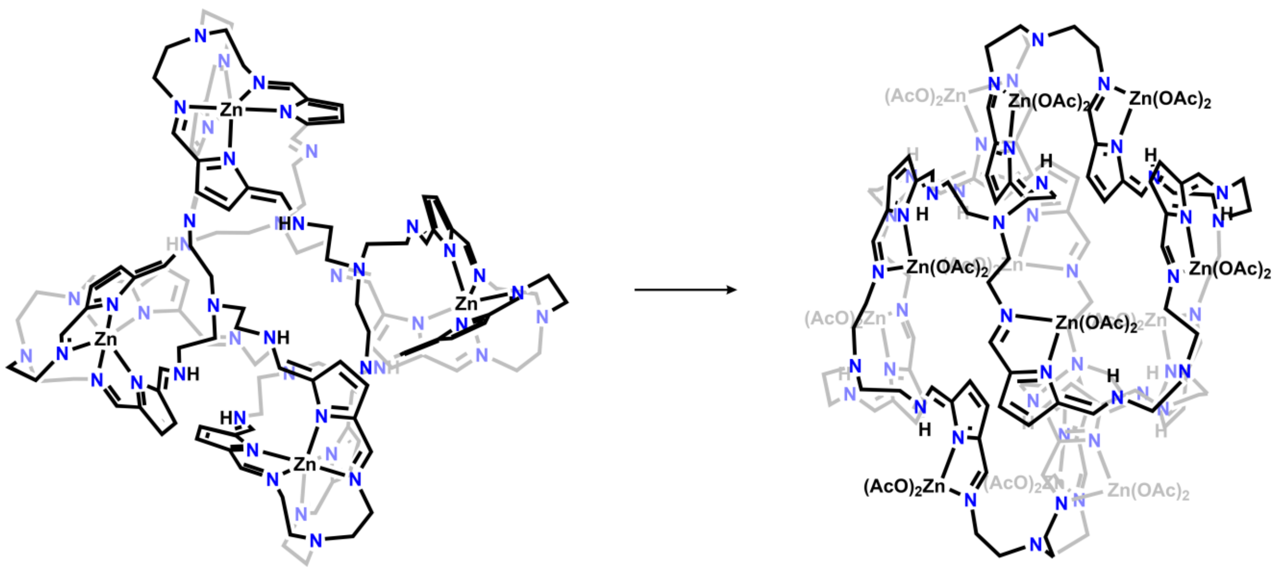

Cage 4-Zn$_4$ (24.6 mg, 0.01 mmol) was placed in a 25 mL round-bottom flask equipped with a magnetic stirrer and dissolved in 5 mL of $CHCl_3$. Zinc(II) acetate dihydrate (26.3 mg, 0.12 mmol) was added, and the mixture was sonicated for 1 minute. Next, 5 mL of $CHCl_3$ was added to the reaction mixture. Flask was closed with a septum, and the mixture was stirred at 25 °C for 17 hours. After completion of the reaction, the solution was concentrated to ca. 2 mL under reduced pressure. MeCN was added in excess, inducing precipitation of the product **3-Zn$_{12}$**. The suspension was transferred to a centrifuge tube and centrifuged for 5 minutes (6000 rpm). The supernatant was separated from the solid, and the MeCN washing procedure was repeated twice. The precipitate was dissolved in $CHCl_3$, and the resulting solution was removed using a rotary evaporator. The obtained, slightly yellowish solid was dried under vacuum for 1 hour. Yield: 37.4 mg (85 %).

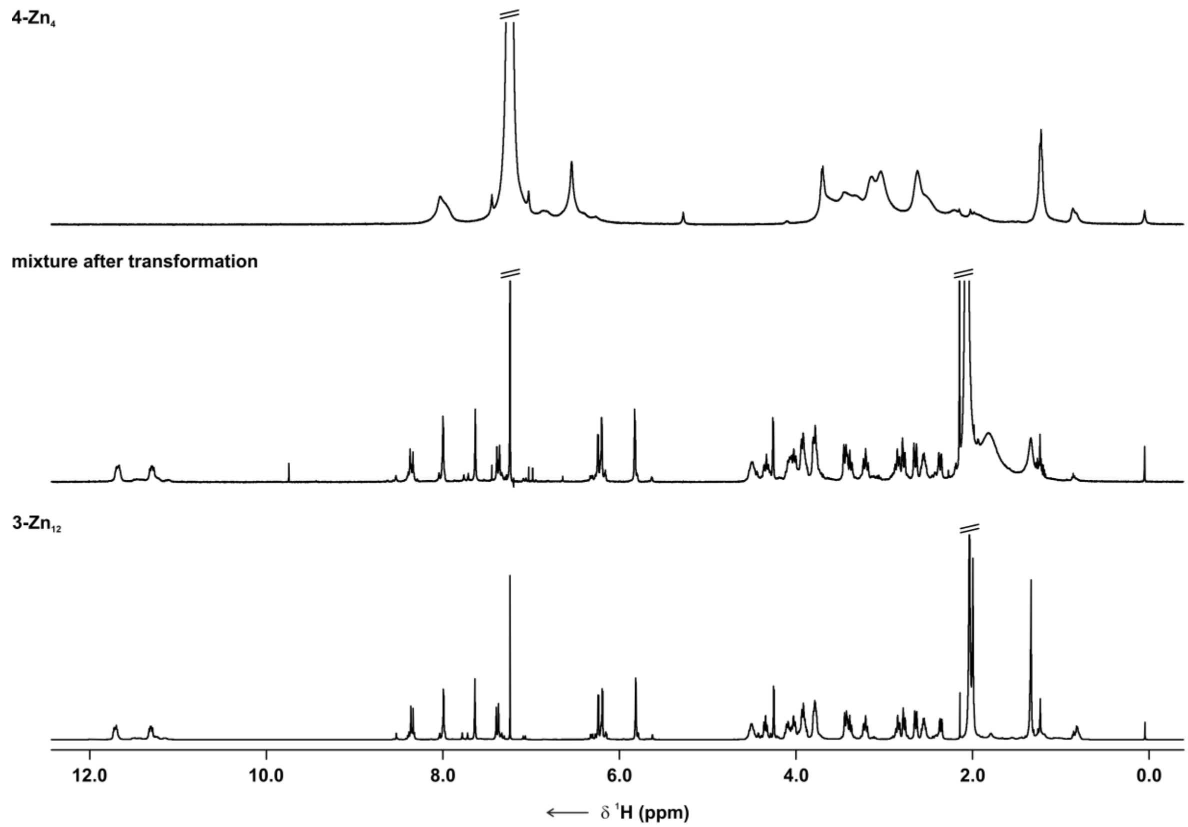

Figure S52. The ^{1}H NMR spectra of **4-Zn$_4$**, **3-Zn$_{12}$**, and crude mixture after transformation of **4-Zn$_4$** to **3-Zn$_{12}$** ($CDCl_3$, 300 K, 600 MHz).

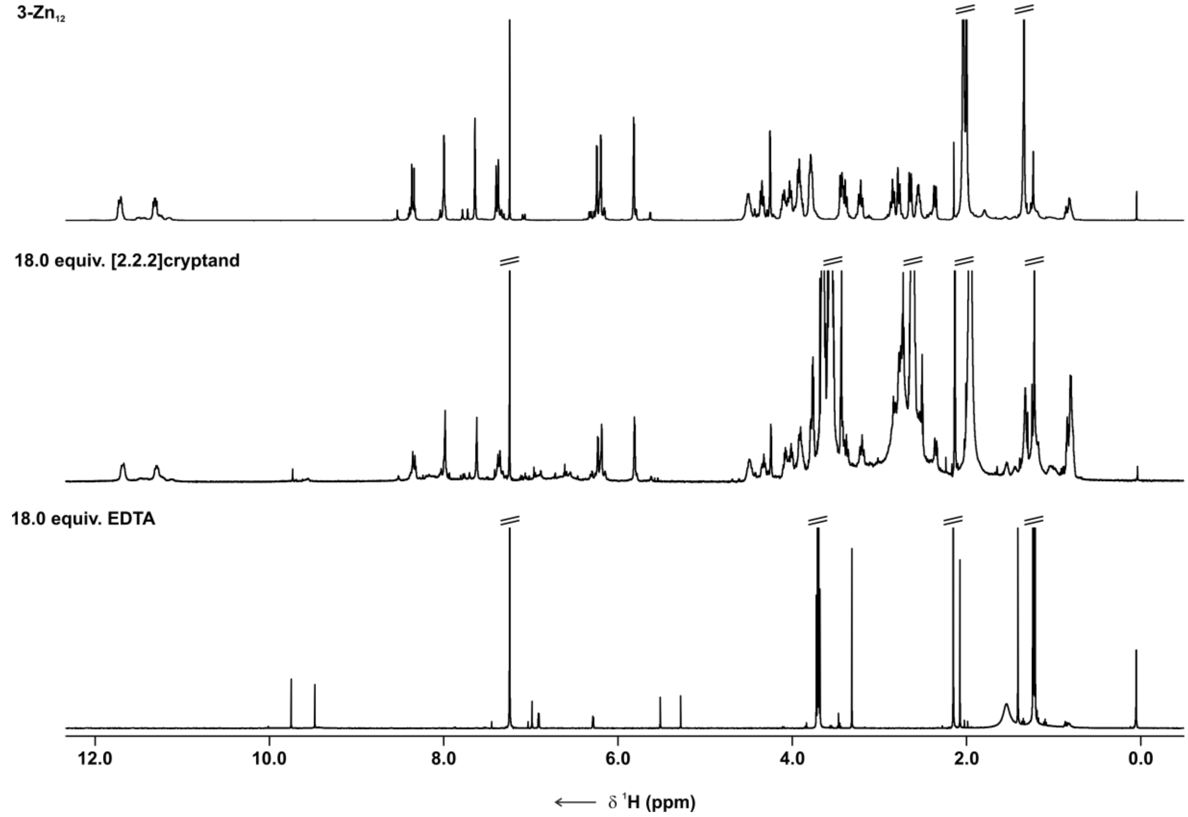

Figure S53. The ^{1}H NMR spectra of **3-Zn$_{12}$** and recorded for the samples after the reaction with 18.0 equiv. of [2.2.2]cryptand or EDTA carried out at 45 °C in MeOH solution ($CDCl_3$, 300 K, 500 MHz).

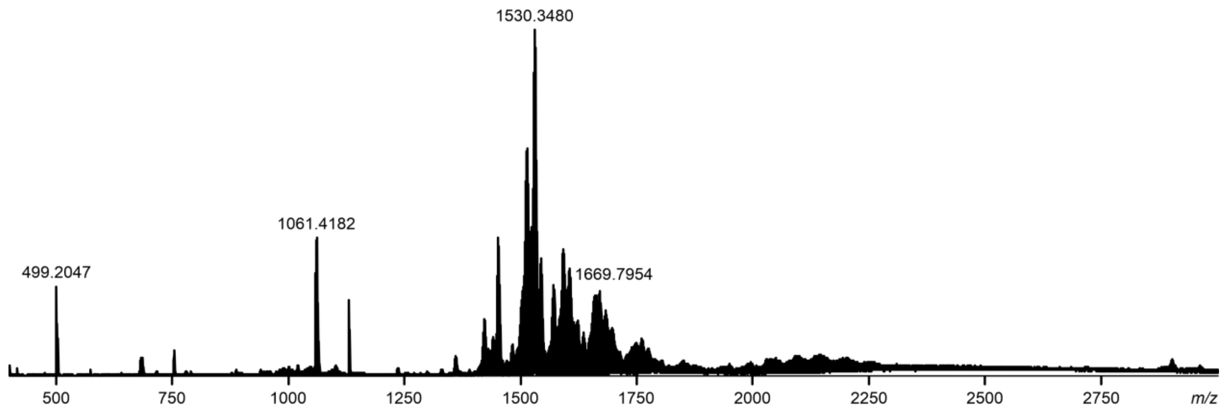

Figure S54. The 400-3000 *m/z* range of the mass spectrum recorded for the mixture obtained upon reacting **3-Zn_{12}** with 18.0 equiv. of [2.2.2]cryptand at 45 °C in MeOH solution (ESI+, TOF).

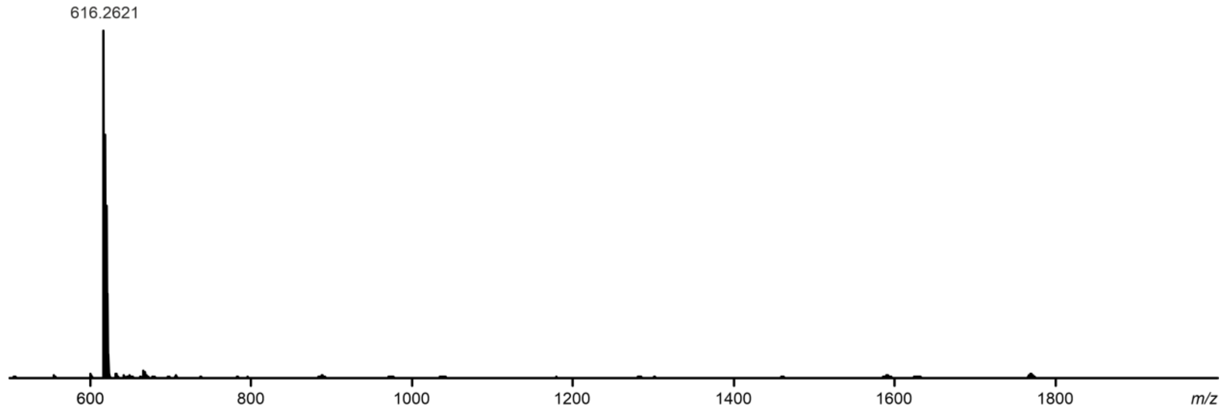

Figure S55. The 500-2000 *m/z* range of the high-resolution mass spectrum recorded for the mixture obtained upon reacting **3-Zn_{12}** with 18.0 equiv. of EDTA at 45 °C in MeOH solution (ESI+, TOF).

X-ray crystallography

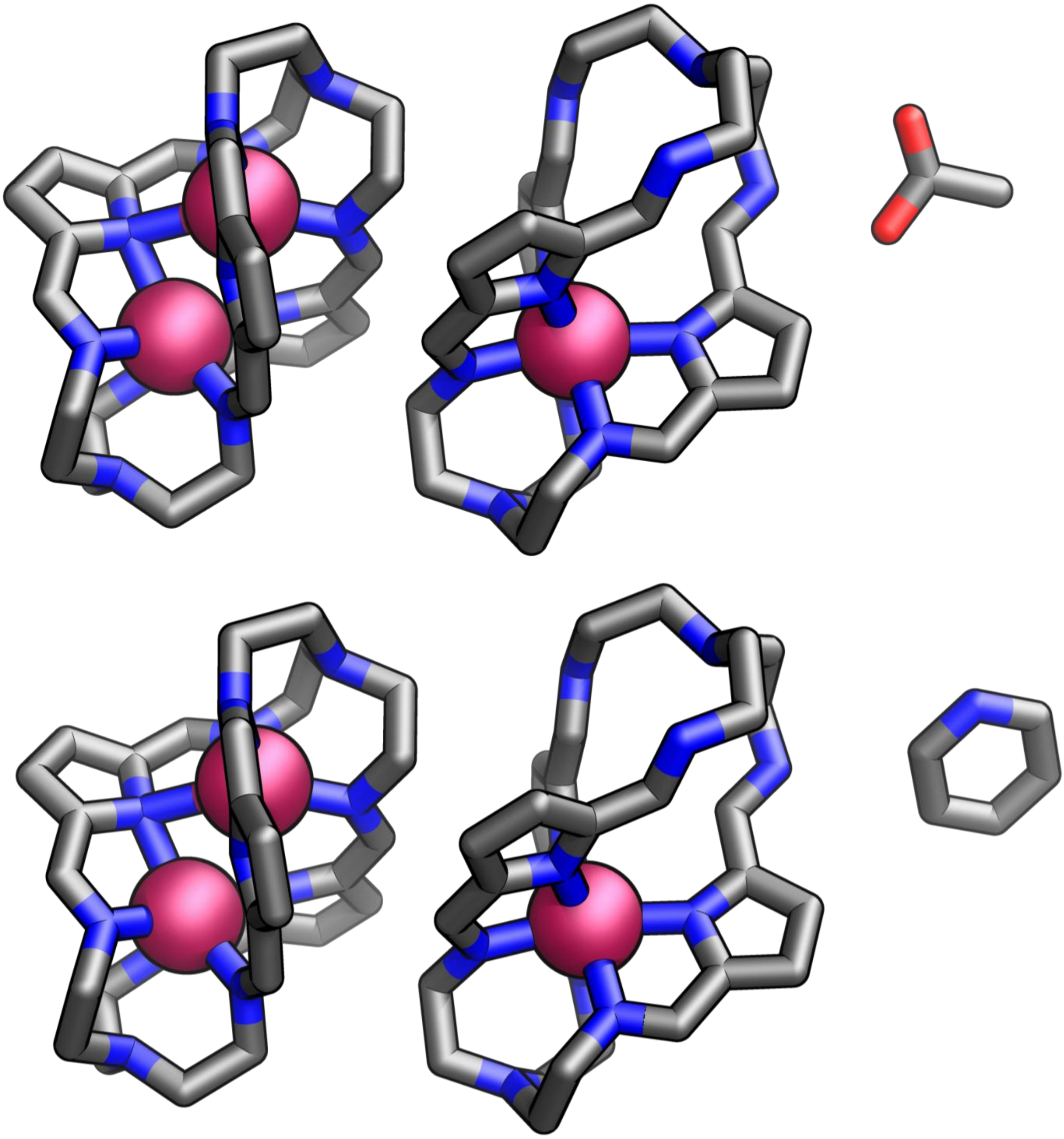

Figure S56. The X-ray molecular structure of **[1-Zn$_2$]$^+$** with two different anions identified in the solid state - **[1-ZnAcOH]$^-$** (top) and **[1-Zn]$^-$** (bottom).

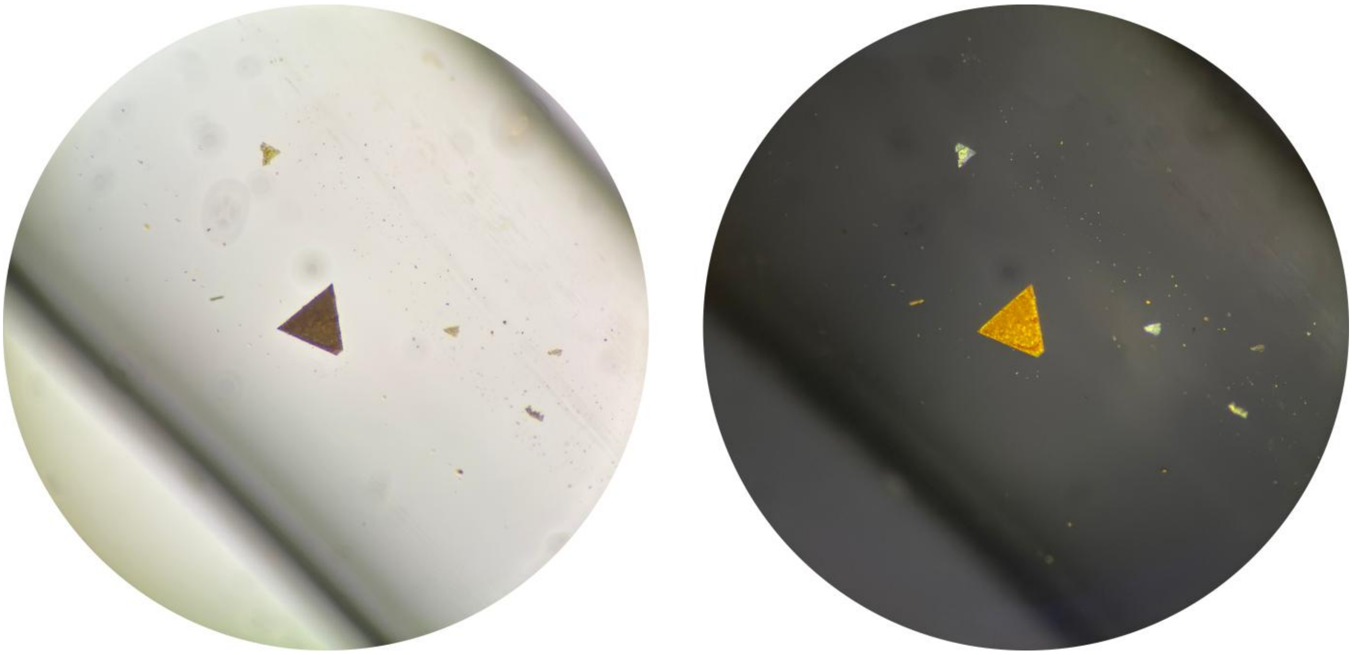

Figure S57. Microscope pictures of crystals of **1-Zn$_2$** without (left) and with polarisation (right).

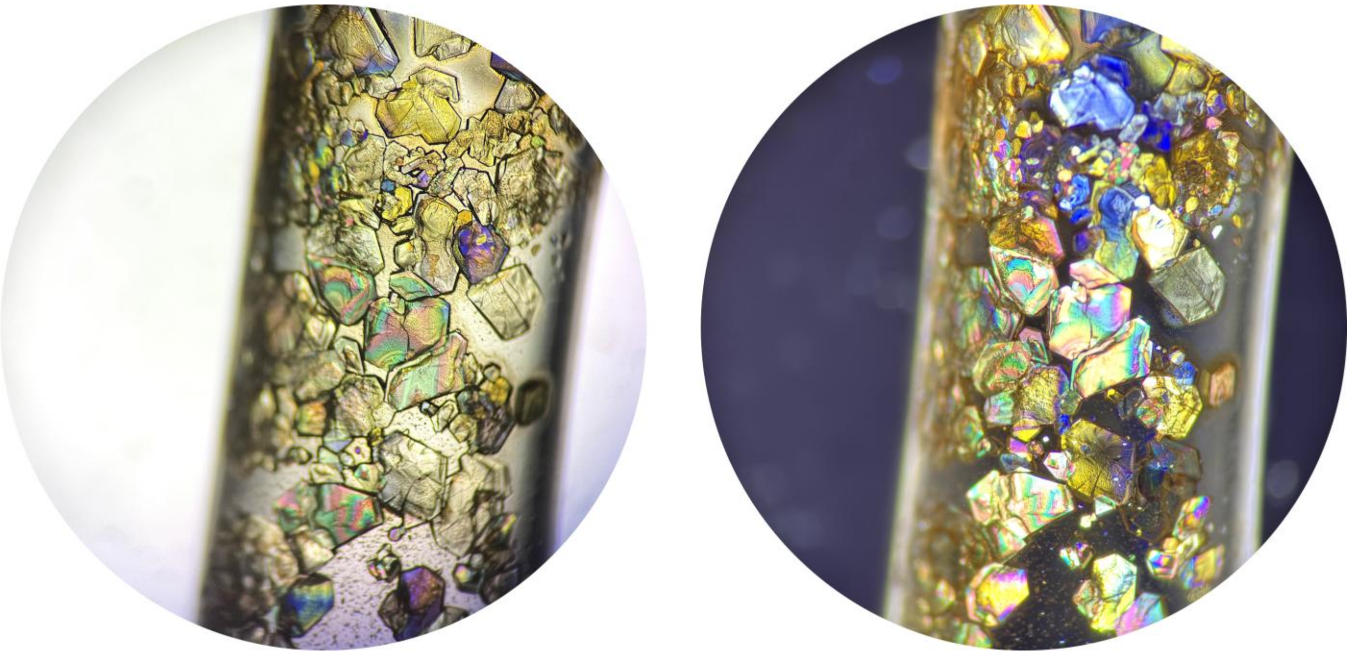

Figure S58. Microscope pictures of crystals of **3-Zn$_{12}$** without (left) and with polarisation (right).

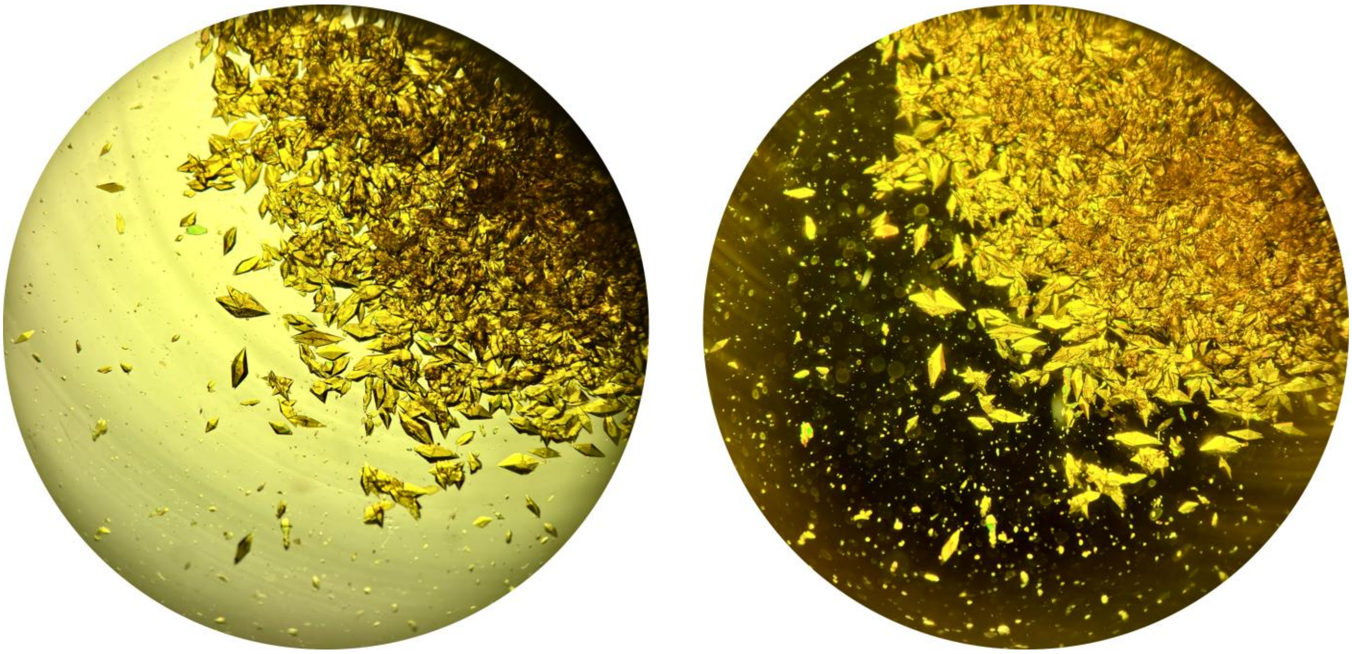

Figure S59. Microscope pictures of crystals of **4-Zn$_4$** without (left) and with polarisation (right).

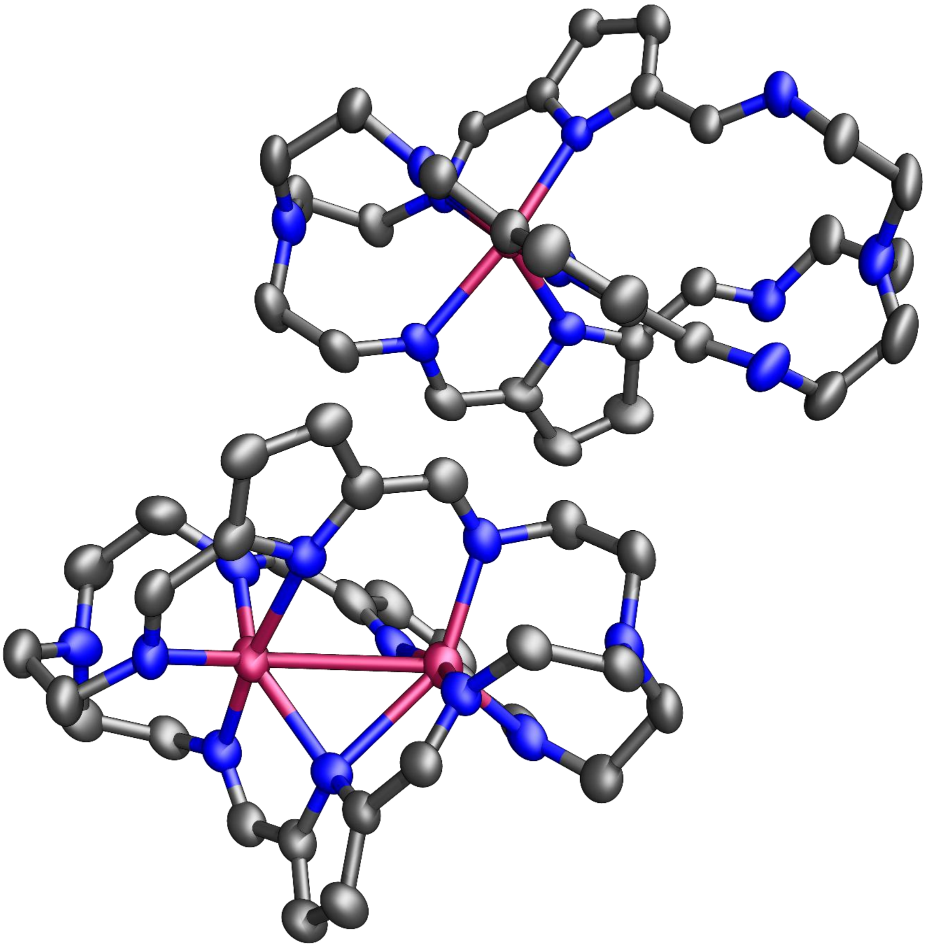

Figure S60. The crystal structure of [**1-Zn$_2$**]$^+$[**1-Zn$_1$**]$^-$. Thermal ellipsoids were depicted at a 50% probability level. Protons and solvents were omitted for the sake of clarity.

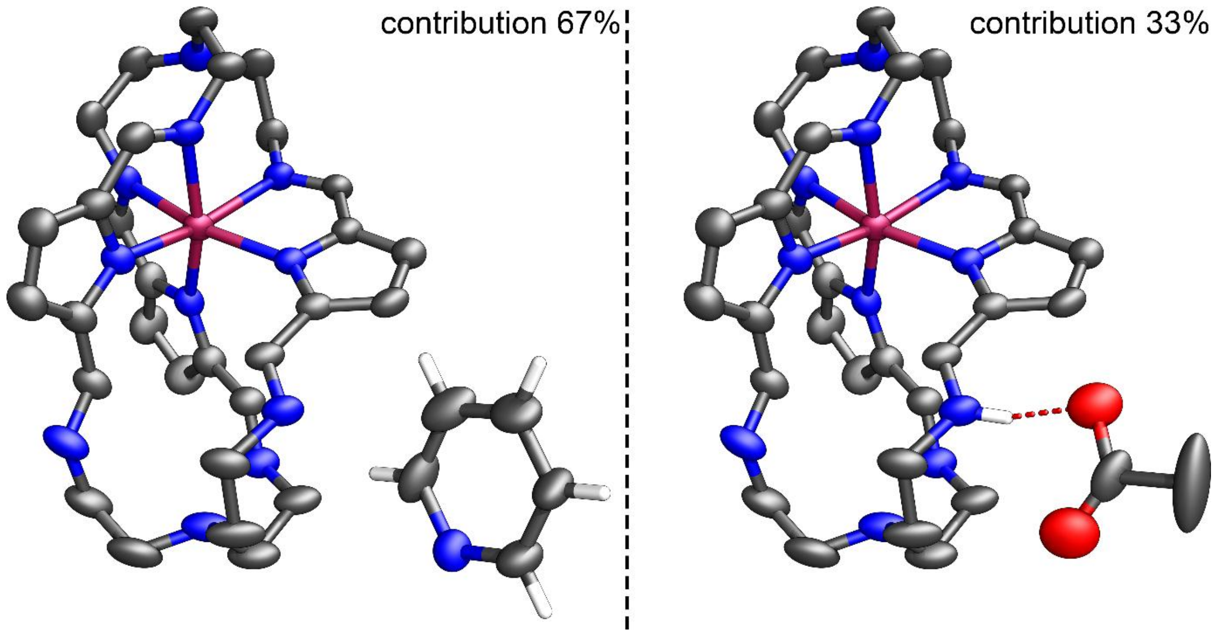

Figure S61. The disorder in the structure of the **[1-Zn$_1$]$^-$**.

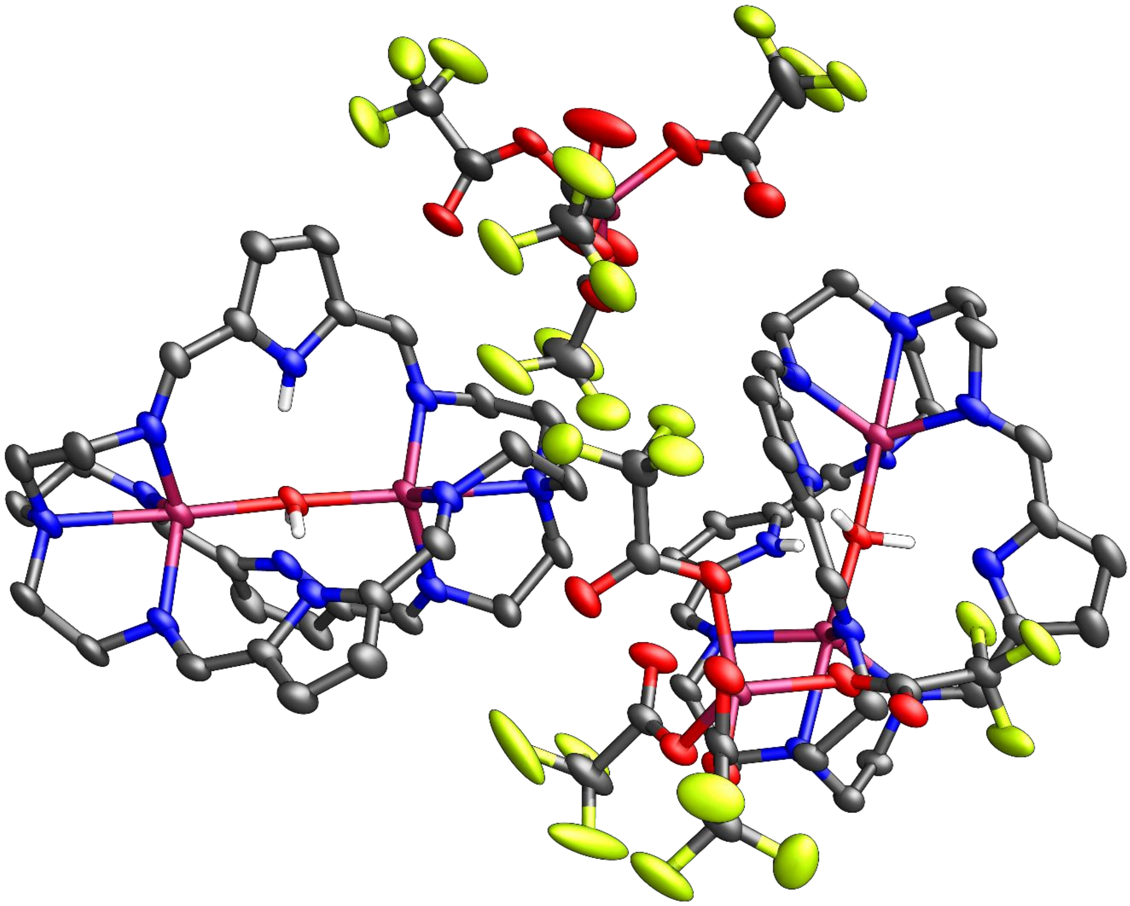

Figure S62. The crystal structure (asymmetric unit) of **[H$_2$O⊂2-Zn$_2$]$^{2+}$[Zn(OOCF$_3$)$_4$]$^{2-}$**. Thermal ellipsoids were depicted at a 50% probability level. Solvents and apolar protons were omitted for the sake of clarity. Similarly, in the case of disordered atoms, only the higher occupancy part was shown.

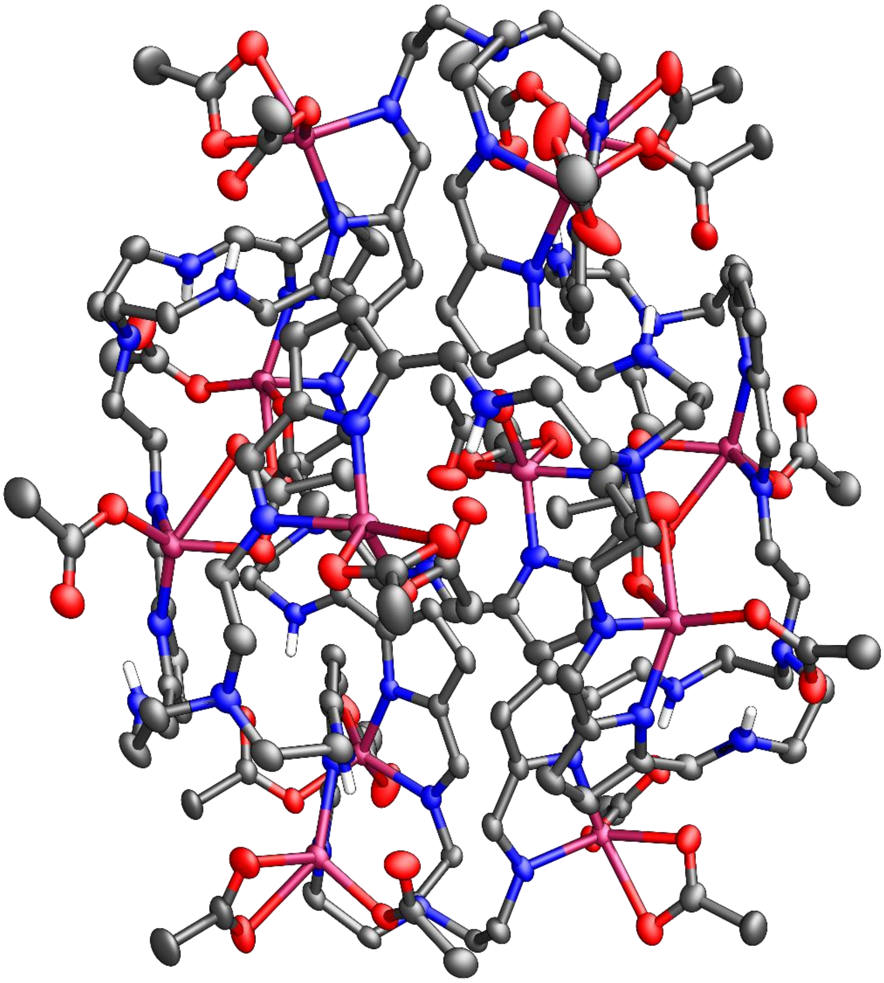

Figure S63. The crystal structure of **3-Zn$_{12}$** (equatorial view). Thermal ellipsoids were depicted at a 50% probability level. Solvents and apolar protons were omitted for the sake of clarity.

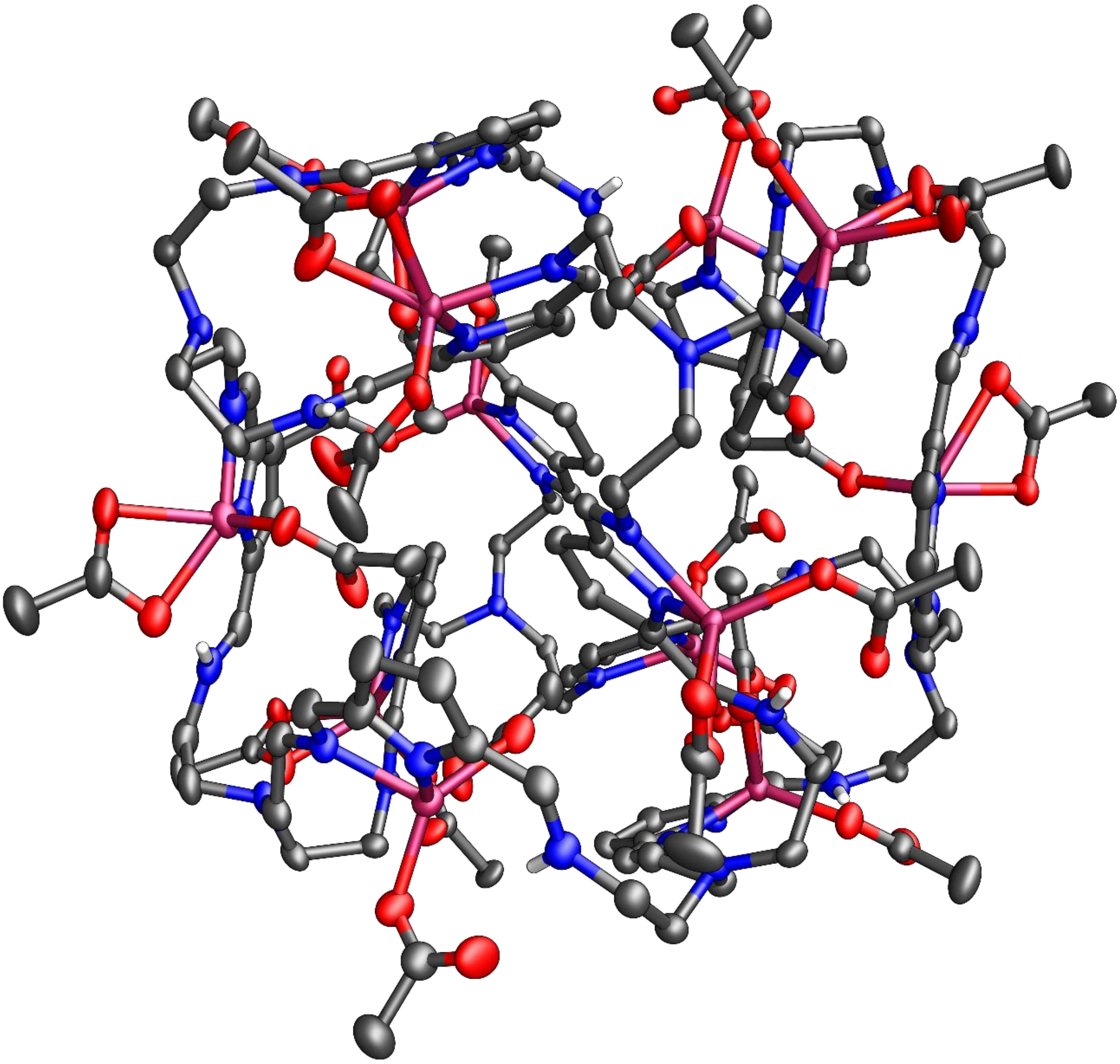

Figure S64. The crystal structure of **3-Zn$_{12}$** (top view). Thermal ellipsoids were depicted at a 50% probability level. Solvents and apolar protons were omitted for the sake of clarity.

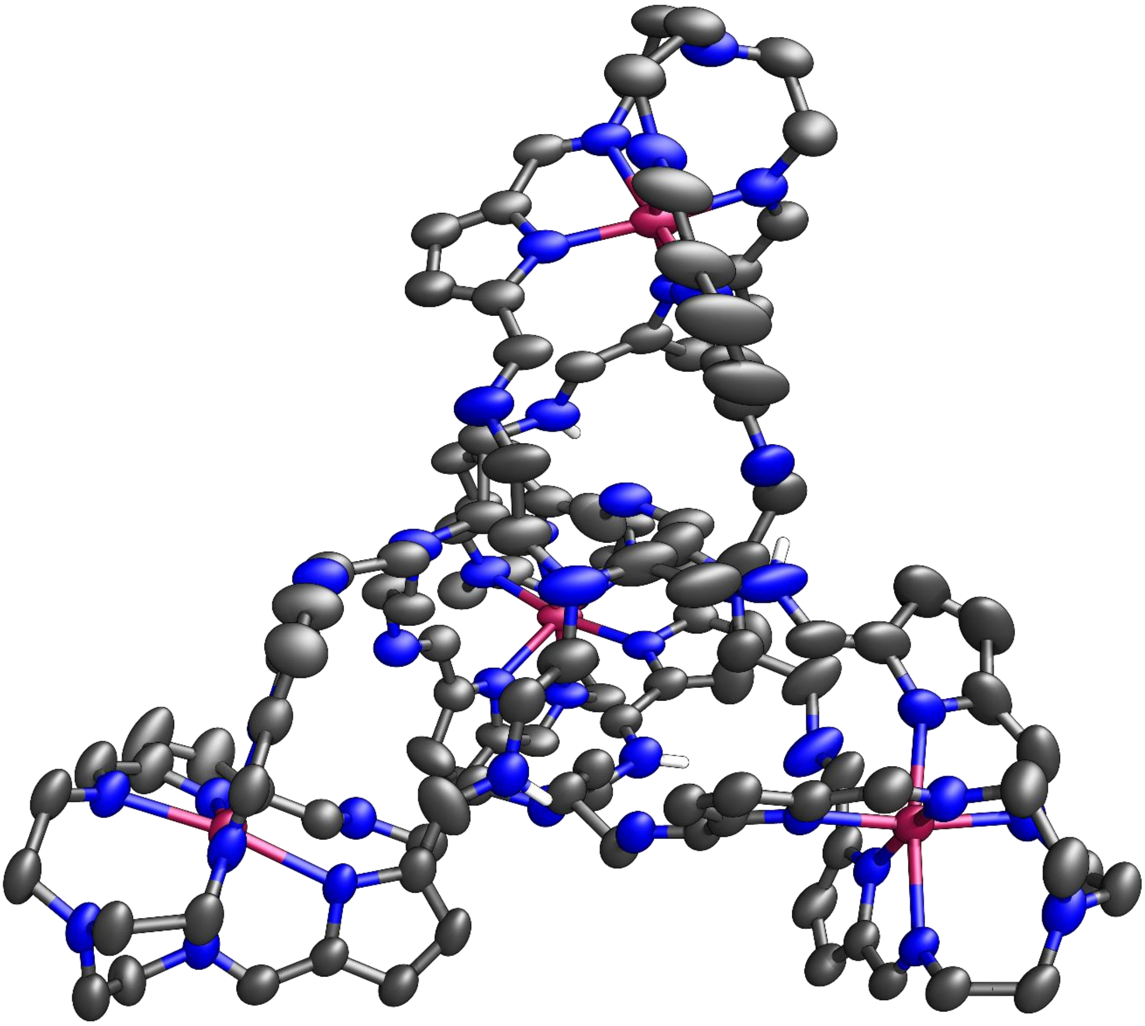

Figure 15. The crystal structure of **4-Zn$_4$** (top view). Thermal ellipsoids were depicted at a 50% probability level. Solvents and apolar protons were omitted for the sake of clarity. Similarly, in the case of disordered atoms, only the higher occupancy part was shown.

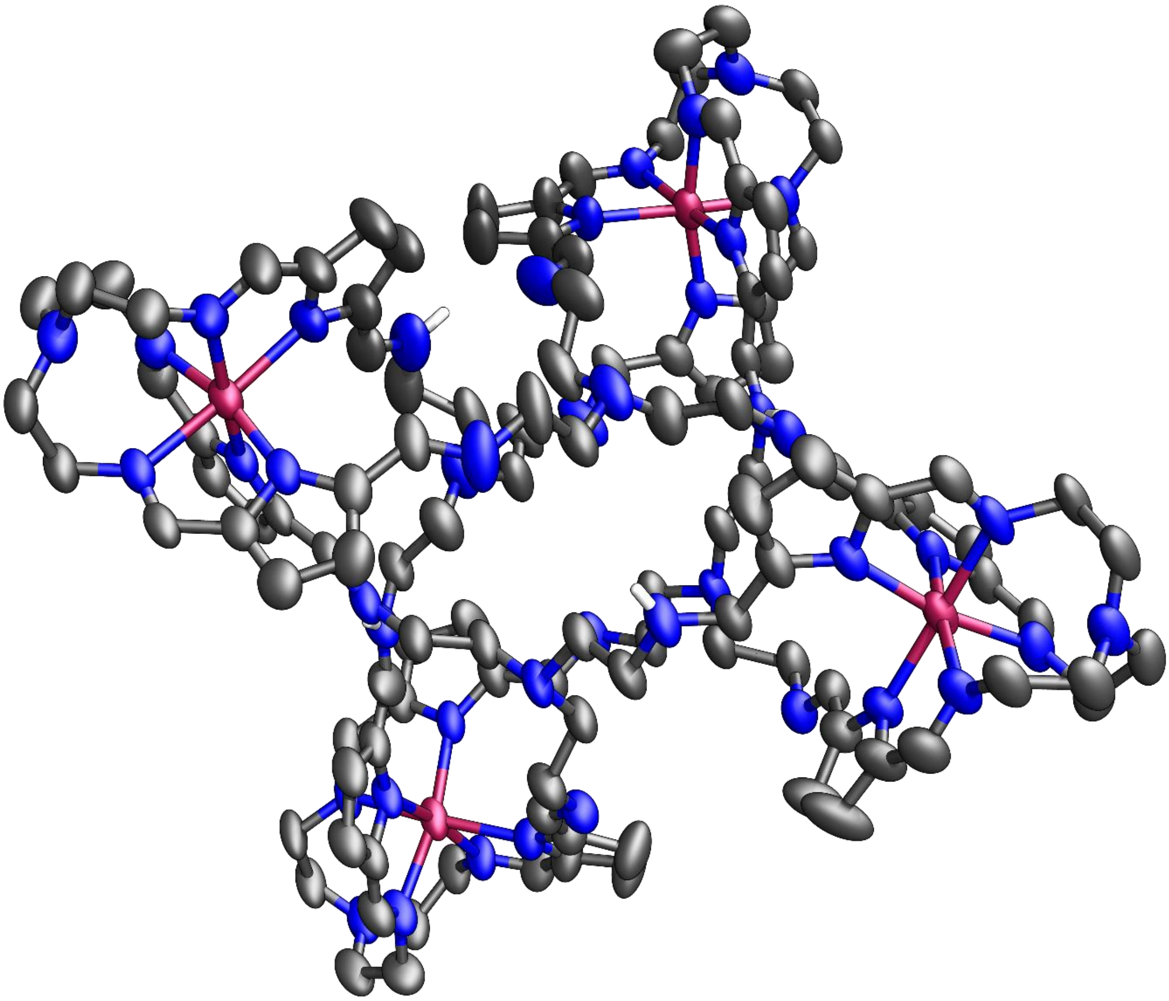

Figure S66. The crystal structure of **4-Zn$_4$** (side view). Thermal ellipsoids were depicted at a 50% probability level. Solvents and apolar protons were omitted for the sake of clarity. Similarly, in the case of disordered atoms, only the higher occupancy part was shown.

Table S1. Selected X-ray diffraction data.

Structure	1-Zn_2	[$H_2O \subset$2-Zn_2]	3-Zn_{12}	4-Zn_4
**Crystal system**	orthorhombic	monoclinic	monoclinic	orthorhombic
**Space group**	$Pna2_1$	$P2_1/c$	$P2_1/c$	$Fdd2$
**Z,Z'**	4, 1	8, 2	4, 1	8, 0.5
**a [Å]**	28.115(7)	30.884(9)	37.691(5)	64.425(9)
**b [Å]**	11.939(3)	11.711(3)	20.472(3)	32.478(4)
**c [Å]**	22.967(6)	31.050(9)	41.463(6)	19.185(3)
**α [°]**	90	90	90	90
**β [°]**	90	117.60(2)	110.52(2)	90
**γ [°]**	90	90	90	90
**Volume [$Å^3$]**	7709(3)	9952(5)	29963(8)	40143(10)
**Reflections collected**	36702	122157	243496	119198
**Reflections independent**	12971	19883	58459	19467
**Reflections observed (I > 2/s(I))**	9737	15113	45536	17970
**Completeness [%]**	99.8	99.9	99.2	100
**R_{int}**	0.0290	0.0486	0.0491	0.0216
**R [$F^2>2\sigma(F^2)$], wR, S**	0.0398, 0.0890, 1.007	0.0557, 0.1635, 1.085	0.0762, 0.2246, 1.069	0.0676, 0.1992, 1.035
**$\Delta\rho_{max}$, $\Delta\rho_{min}$ [$eÅ^{-3}$]**	0.298, −0.385	0.960, −0.853	2.063, −1.381	0.514, −0.429

References

[86] Rigaku Oxford Diffraction, (2023), CrysAlisPro Software system, version 171.43.104a, Rigaku Corporation, Wrocław, Poland

[87] G. M. Sheldrick, *Acta Crystallogr. Sect. A: Found. Adv.* **2015**, *71*, 3–8.

[88] G. M. Sheldrick, *Acta Crystallogr. Sect. C: Struct. Chem.* **2015**, *71*, 3–8.

[89] O. V. Dolomanov, L. J. Bourhis, R. J. Gildea, J. A. K. Howard, H. Puschmann, *J. Appl. Crystallogr.* **2009**, *42*, 339–341.

[90] S. Hammes-Schiffer, A. A. Stuchebrukhov, *Chem. Rev.* **2010**, *110*, 6939–6960.

[91] K. S. Peters, *Acc. Chem. Res.* **2009**, *42*, 89–96.

[92] G. M. Peters, J. B. Winegrad, M. R. Gau, G. H. Imler, B. Xu, S. Ren, B. B. Wayland, M. J. Zdilla, *Inorg. Chem.* **2017**, *56*, 3377–3385.
